# Comprehensive 2D Gas Chromatography with TOF-MS Detection Confirms the Matchless Discriminatory Power of Monoterpenes and Provides In-Depth Volatile Profile Information for Highly Efficient White Wine Varietal Differentiation

**DOI:** 10.3390/foods9121787

**Published:** 2020-12-02

**Authors:** Igor Lukić, Silvia Carlin, Urska Vrhovsek

**Affiliations:** 1 Institute of Agriculture and Tourism, K. Huguesa 8, 52440 Poreč, Croatia; 2 Centre of Excellence for Biodiversity and Molecular Plant Breeding, Svetošimunska 25, 10000 Zagreb, Croatia; 3 Department of Food Quality and Nutrition, Research and Innovation Centre, Fondazione Edmund Mach (FEM), Via E. Mach 1, 38098 San Michele all’Adige, TN, Italy; silvia.carlin@fmach.it (S.C.); urska.vrhovsek@fmach.it (U.V.)

**Keywords:** two-dimensional gas chromatography, one-dimensional, wine, volatile aroma compounds, multivariate analysis, cultivar, Croatia

## Abstract

To differentiate white wines from Croatian indigenous varieties, volatile aroma compounds were isolated by headspace solid-phase microextraction (HS-SPME) and analyzed by comprehensive two-dimensional gas chromatography with time-of-flight mass spectrometry (GC×GC-TOF-MS) and conventional one-dimensional GC-MS. The data obtained were subjected to uni- and multivariate statistical analysis. The extra separation ability of the GC×GC second dimension provided additional in-depth volatile profile information, with more than 1000 compounds detected, while 350 were identified or tentatively identified in total by both techniques, which allowed highly efficient differentiation. A hundred and sixty one compounds in total were significantly different across monovarietal wines. Monoterpenic compounds, especially α-terpineol, followed by limonene and linalool, emerged as the most powerful differentiators, although particular compounds from other chemical classes were also shown to have notable discriminating ability. In general, Škrlet wine was the most abundant in monoterpenes, Malvazija istarska was dominant in terms of fermentation esters concentration, Pošip contained the highest levels of particular C_13_-norisoprenoids, benzenoids, acetates, and sulfur containing compounds, Kraljevina was characterized by the highest concentration of a tentatively identified terpene γ-dehydro-ar-himachalene, while Maraština wine did not have specific unambiguous markers. The presented approach could be practically applied to improve defining, understanding, managing, and marketing varietal typicity of monovarietal wines.

## 1. Introduction

Aroma is among the most important attributes that drive the perception of wine sensory quality and varietal typicity by consumers. It results from the occurrence of many diverse odoriferous volatile compounds of different origin. Primary or varietal aroma compounds originate from grapes, secondary or fermentation aroma compounds are produced in fermentation, while tertiary aromas are formed during maturation [1,2,3]. The three groups mentioned are not so clearly divided: most of the precursors of volatile aroma compounds originate from grapes and are in one way or another affected by fermentation and/or aging [4]. The final wine aroma profile is a result of complex interactive effects between many sources of variability, such as variety [5], geographical position characterized by specific agroecological conditions [6,7], viticultural practices [8], harvest date [9], harvest year [10,11], grape processing, and fermentation parameters [12,13], etc. 

Varietal characterization (description) and differentiation (contradistinction from other varieties) is an ever-important field of wine research. Many studies have aimed to identify volatile compounds characteristic for various grape varieties, since they are crucial for the typical varietal attributes of their wines. The knowledge on the volatile aroma compound composition of monovarietal wines is important since it may enable producers to better cope with the phenomena encountered in production and to manage vinification with greater efficiency, all in order to produce high quality wines of accentuated varietal typicity. It may enable detailed and precise description of the aroma of monovarietal wines, which could be used in their marketing, especially towards informed consumers interested in wines of high quality with marked diversity and identity. In addition to often being linked to a given geographical provenance with a corresponding protected designation of origin (PDO), particular monovarietal wines are especially appreciated and demanded because of their typical sensory properties. Such wines often fall within a higher price range and are a target of counterfeiting by mislabeling their varietal origin. Therefore, control in terms of varietal origin authentication is needed: the general strategy used by many research groups includes the (semi)quantification of a large number of volatile compounds in large sets of wines and use of the generated data for the production of multivariate statistical models able to classify wines, as well as to predict and confirm their varietal origin [5].

The analysis of volatile aroma compounds in wine varietal characterization and differentiation studies is commonly performed by conventional one-dimensional gas chromatography mass spectrometry (GC-MS) [14,15,16,17,18,19]. Although the information obtained by this approach is often sufficient to obtain more or less efficient varietal differentiation, a large amount of information is lost due to frequent co-elutions, even when using long GC run times on high-efficiency capillary columns with selective stationary phases and programmed oven temperature conditions [20,21]. In the last few decades, comprehensive two-dimensional gas chromatography-mass spectrometry (2D-GC-MS or GC×GC-MS) stood out as a highly potent technique for in-depth characterization of complex samples [22], where the number of compounds of interest is large and many are present at trace levels, as in wine. This technique utilizes two GC columns of different stationary phases serially connected by a modulator, where the compounds co-eluting in the first column are in most cases separated in the second. GC×GC-MS is therefore characterized by higher efficiency and sensitivity, since the additional separation by a second stationary phase produces clearer mass spectra and much less chromatographic peaks remain unannotated. In this way, GC×GC-MS allows detection and identification of a much larger number of volatile compounds compared to conventional GC-MS [23].

Regardless of the existing great potential, only a few studies have utilized GC×GC to investigate wine volatile aroma profiles, while studies which used GC×GC for varietal characterization and differentiation were extremely rare. Several authors reported more or less detailed GC×GC volatile aroma profiles of particular monovarietal wines, such as Cabernet Sauvignon [24], Sauvignon Blanc [25], Shiraz [9,26] or Syrah [12], Pinotage [21], Chardonnay [27], and Verdicchio [28], but none of them directly compared them to or differentiated them from other monovarietal wines of similar typology. In this way, despite detailed profiles determined in some cases, it still remained unknown which compounds and in which amounts are typical for a given variety and whether they could differentiate it from other monovarietal wines. The only two studies which utilized GC×GC and succeeded in differentiating several monovarietal wines did not report actual concentrations of all the identified volatile compounds [20,29].

The aim of this study was to utilize the potential of two-dimensional gas chromatography with time-of-flight mass spectrometry (GC×GC-TOF-MS) technique, in combination with headspace solid-phase microextraction (HS-SPME) and multivariate statistical tools, as a more efficient approach to characterize and differentiate monovarietal white wines based on their volatile aroma compound composition. Profiling by GC×GC was combined with conventional GC-MS analysis of major wine volatile compounds to obtain more comprehensive aroma profiles. Special attention was devoted to terpenes, often highlighted as key varietal markers in wine. The approach was applied to characterize and differentiate Croatian wines made from indigenous grape varieties, with each variety represented by a rather heterogeneous group of wines with respect to geographical microlocation and agroecological conditions, viticultural practices, harvest date, and grape processing and wine production parameters. It was expected that GC×GC-TOF-MS would be extremely effective in providing novel in-depth information for efficient white wine varietal differentiation.

## 2. Materials and Methods

### 2.1. Wine Samples 

A total of 32 wines made from Croatian indigenous white grape varieties (*Vitis vinifera* L.) Malvazija istarska (MI, 8 samples) Pošip (PO, 7), Maraština (MA, 7), Kraljevina (KR, 7), and Škrlet (SK, 3) were donated by producers from Croatia (EU), more specifically Istria (MI) and Dalmatia (PO and MA) as the coastal regions and continental Croatia (KR and SK). Wines from the same variety were donated by different producers. The selection was representative for Croatian wine production and comprised the majority of the most important Croatian indigenous varieties. Only young wines from harvest 2015 were collected, labelled with a protected designation of origin (PDO) and with a traditional term “Quality or Top quality” wine. Wines were of the same typology and produced by standard white winemaking technology, which included grape harvest at technological maturity, destemming, crushing and mashing of the grapes, no or short pre-fermentative skin-contact (up to 48 h), use of selected commercial yeasts, fermentation at relatively low temperatures (up to 18 °C), and other standard procedures (sulfiting, racking, fining, and stabilization, etc.). Wines were not in contact with wood. During the period from harvest and vinification in September 2015 until the collection and analyses in April and May 2016 the wines were stored in stainless steel tanks and 0.75 L glass bottles with cork stoppers in wine cellars of the producers. The wine samples were selected from a larger set as typical representatives of a given variety by the panel for wine sensory analysis of the Institute of Agriculture and Tourism in Poreč (Croatia), which consisted of highly trained and experienced tasters. Standard physico-chemical parameters of the collected wines determined by OIV methods are reported in Table S1.

### 2.2. Standards, Chemicals, and Consumables

Chemical standards of volatile aroma compounds were procured from AccuStandard Inc. (New Haven, CT, USA), Fluka (Buchs, Switzerland), Honeywell International Inc. (Morris Plains, NJ, USA), Merck (Darmstadt, Germany), and Sigma-Aldrich (Sigma-Aldrich, St. Louis, MO, USA). A stock solution of major volatile compounds commonly present in wine was prepared in methanol, while standard solutions were prepared in model wine (13 vol.% of ethanol, pH 3.3). Ammonium sulfate and sodium chloride were purchased from Kemika d.d (Zagreb, Croatia). 

Divinylbenzene/carboxen/polydimethylsiloxane (DVB-CAR-PDMS, StableFlex, 50/30 μm, 1 cm) SPME fiber used for GC-MS analysis was procured from Supelco, Sigma Aldrich (Bellafonte, PA, USA) and DVB-CAR-PDMS SPME fiber (StableFlex, 50/30 μm, 2 cm) used for GC×GC-TOF-MS analysis was procured from Supelco, Sigma Aldrich (Milan, Italy).

### 2.3. Analysis of Volatile Aroma Compounds by Conventional One-Dimensional GC-MS

Volatile aroma compounds for GC-MS analysis were isolated by headspace solid-phase microextraction (HS-SPME) according to the modified method proposed by Bubola et al. [30]. Four milliliters of a solution obtained by diluting wine four times with deionized water were pipetted in a 10 mL glass vial. Ammonium sulfate (1 g) and 50 μL of internal standards solution (2-octanol (0.84 mg/L), 1-nonanol (0.82 mg/L), and heptanoic acid (2.57 mg/L)) were added. After 15 min preconditioning at 40 °C, microextraction using a DVB-CAR-PDMS SPME fiber took place for 40 min at 40 °C with stirring (800 rpm). Volatile compounds were desorbed after the insertion of the fiber for 10 min into a GC/MS injector heated at 248 °C, with the first 3 min in splitless mode. Volatile aroma compounds were identified and quantified using a Varian 3900 gas chromatograph (GC) connected to a Varian Saturn 2100T mass spectrometer with an ion trap analyzer (Varian Inc., Harbour City, CA, USA). The column used was a 60 m × 0.25 mm i.d. × 0.25 μm d.f. Rtx-WAX (Restek, Belafonte, PA, USA). Initial temperature of the GC oven was 40 °C, ramped up at 2 °C/min to reach 240 °C, and then kept at this temperature for additional 10 min. Helium was used as a carrier gas at a flow rate of 1.2 mL/min. Mass spectra were acquired in EI mode (70 eV), at 30–350 *m/z*. 

Identification of volatile compounds was conducted by comparison of retention times and mass spectra of the analytes with those of pure standards, and with mass spectra from NIST05 library. Identification by comparison with mass spectra was considered satisfactory if spectra reverse match numbers (RM) higher than 800 were obtained. In the case of less clear spectra (RM < 800) identification was considered satisfactory if the ratios of the relative intensities of a quantifier ion and three characteristic ions with the highest intensity reasonably matched those in the reference spectra of a given compound. Linear retention indices were calculated with respect to the retention times of C_10_ to C_28_ n-alkanes and compared to those reported in literature for columns of equal or equivalent polarity. Calibration curves were constructed based on the analysis of standard solutions containing known concentrations of standards at six concentration levels and were used for quantification. Quantification of major volatile compounds was based on total ion current peak area, while quantification of minor compounds was based on quantifier ion peak area. The peak areas and concentrations in standard solutions and in wine samples were normalized with respect to those of the internal standards. Linearity was satisfactory with coefficient of determination higher than 0.99 for all the standards. Relative standard deviation of repeatability (RSD) was determined after repeated analysis (*n* = 5) of a Malvazija istarska wine sample and was satisfactory, with RSD lower than 13.05% for monoterpenes, 7.38 for β-damasenone, lower than 9.23% for alcohols, 7.34 for ethyl esters, 12.34% for acetate esters, and 11.78% for fatty acids. Method validation parameters were previously published in the study of Bubola et al. [30]. In the cases when pure chemical standards were not available, semi-quantitative analysis was carried out. The concentrations of such compounds were expressed as equivalents of compounds with similar chemical structure which were quantified using calibration curves, assuming a response factor equal to one.

### 2.4. Analysis of Volatile Aroma Compounds by GC×GC-TOF-MS

A volume of 2.5 mL of wine was transferred to a 20 mL headspace vial and 1.5 g of sodium chloride was added. Wine sample was spiked with 50 μL of internal standard (2-octanol, 1 mg/L). Quality control samples (QC) were prepared by mixing equal proportion of each sample and were analyzed before the samples sequence (*n* = 5) and after every five samples (*n* = 1). GC×GC-TOF-MS analysis of wines was performed using a GC Agilent 7890N (Agilent Technologies, Palo Alto, CA, USA) coupled to a LECO Pegasus IV time-of-flight mass spectrometer (TOF-MS) (Leco Corporation, St. Joseph, MI, USA) equipped with a Gerstel MPS autosampler (GERSTEL GmbH & Co. KG, Mülheim an der Ruhr, Germany), as described in previous studies with minor modifications [9,31,32]. Briefly, samples were preconditioned at 35 °C for 5 min and volatile compounds were extracted using a DVB/CAR/PDMS SPME fiber for 20 min. Volatile compounds were desorbed for 3 min at 250 °C in splitless mode. The fiber was reconditioned for 7 min at 270 °C between each extraction. Helium was used as a carrier gas at a flow rate of 1.2 mL/min. The oven was equipped with a 30 m × 0.25 mm × 0.25 μm film thickness VF-WAXms column (Agilent Technologies) in the first dimension (1D) and a 1.5 m × 0.15 mm × 0.15 μm film thickness Rxi 17Sil MS column (Restek) in the second dimension (2D). Initial oven temperature was maintained at 40 °C for 4 min, then raised at 6 °C/min to 250 °C, and then finally maintained at this temperature for additional 5 min. The second oven was maintained at 5 °C above the temperature of the first one throughout the analysis. The modulator was offset by +15 °C in relation to the secondary oven, the modulation time was 7 s with 1.4 s of hot pulse duration, as described previously [31]. Electron ionization at 70 eV was applied, the temperature of ion source was 230 °C, detector voltage was 1317 V, mass range (*m/z*) was 40–350, acquisition rate was 200 spectra/s, and acquisition delay was 120 s.

Baseline correction, chromatogram deconvolution and peak alignment were performed using LECO ChromaTOF software version 4.32 (Leco Corporation, St. Joseph, MI, USA). The baseline offset was set to 0.8 and signal to noise (S/N) ratio was set at 100. Peak width limits were set to 42 s and 0.1 s in the first and the second dimension, respectively. Traditional, not adaptive integration was used. The required match (similarity) to combine peaks was set to 650. Under these conditions 1025 putative compounds were detected. Volatile compounds were identified by comparing their retention times and mass spectra with those of pure standards and with mass spectra from NIST 2.0, Wiley 8, and FFNSC 2 (Chromaleont, Messina, Italy) mass spectral libraries, with a minimum library similarity match factor of 750 out of 999. For identification of compounds by comparison with pure standards, a mix of 122 compounds was injected under identical GC×GC-TOF-MS conditions. For tentative identification of compounds and/or confirmation of their identities determined as described above, linear retention indices were calculated with respect to the retention times of C_10_ to C_30_ n-alkanes and compared to those from literature for conventional one-dimensional GC obtained using columns of equal or equivalent polarity (NIST 2.0, Wiley 8, FFNSC 2, VCF, ChemSpider). Three hundred and seventeen (317) volatile aroma compounds were (tentatively) identified in total. Volatile compounds were semi-quantified and their concentrations in μg/L were calculated relative to the internal standard 2-octanol, assuming a response factor equal to one.

In preliminary tests by principal component analysis (PCA), QC samples were clustered very close and were very well separated from the wine samples, suggesting the repeatability of the method was very good. Relative standard deviation of the internal standard 2-octanol in QC samples was 10.4% which was considered satisfactory for HS-SPME/GC×GC-TOF-MS analysis.

### 2.5. Statistical Data Elaboration

Data obtained by GC-MS and GC×GC-TOF-MS were processed by analysis of variance (one-way ANOVA). Least significant difference (LSD) post-hoc test was used to compare the mean values of concentrations at *p* < 0.05. Multivariate analysis of data was performed by PCA and forward stepwise linear discriminant analysis (SLDA). The original dataset which included 32 wines and 350 volatile aroma compounds (33 determined by GC-MS + 317 determined by GC×GC-TOF-MS analysis; in the case of compounds determined by both techniques GC×GC-TOF-MS data were used), was reduced based on Fisher ratios (*F*-ratios). Multivariate techniques were applied on the variables (mean-centered concentrations of volatile compounds) with the highest *F*-ratios. PCA was performed with 40 variables with the highest *F*-ratio, while SLDA and hierarchical clustering were performed with 60 variables with the highest *F*-ratio, in both cases with GC-MS and GC×GC-TOF-MS data combined. Two additional SLDA models were built with the concentrations of terpenes which were significantly different between wines, using GC-MS and GC×GC-TOF-MS data separately. In SLDA, variables were selected based on Wilk’s lambda, with *F* to enter = 1 and *F* to remove = 0.5. Cross-validation was applied to check the prediction capacity of the developed SLDA models. ANOVA, PCA, and SLDA were performed by Statistica v. 13.2 software (StatSoft Inc., Tulsa, OK, USA). Hierarchical clustering was conducted and a heatmap was generated by Ward algorithm and Euclidean distance analysis using MetaboAnalyst v. 4.0 (http://www.metaboanalyst.ca), created at the University of Alberta, Canada [33].

## 3. Results and Discussion

### 3.1. GC-MS

Major volatile aroma compounds are highly abundant in wines and for this reason GC-MS was considered appropriate for their analysis. It was considered that their quantitation by GC-MS was not significantly affected by co-eluting compounds. As well, the analysis of major volatiles by GC×GC-TOF-MS would require a rather different setup than that applied in this study, with much larger modulation time and hot pulse duration, not applicable for minor and trace compounds. Major volatile aroma compounds determined by GC-MS are listed in Table 1, grouped according to chemical class, and sorted within each class in order of decreasing *F*-ratio obtained by one-way ANOVA. Twenty-one monoterpenoids and a sesquiterpenoid *trans*-nerolidol, eight C_13_-norisoprenoids, two benzenoids, four alcohols, four acids, and 11 esters were quantified. Table S2 reports the concentrations of the identified volatile compounds in each of the investigated wines.

Among terpenes, major monoterpenols such as linalool, geraniol, α-terpineol, and nerol were found in the highest concentration, which was generally in agreement with previous findings on white wines [34,35,36]. The mentioned are among the most influential monoterpenoids to wine aroma, to which they significantly contribute with specific floral and fruity nuances due to their relatively low odor perception thresholds, such as, for example, 15 μg/L for linalool [35,37]. The highest *F*-ratio among all the compounds identified by GC-MS was determined for α-terpineol, followed by an unidentified monoterpene and linalool, confirming the importance of terpenes for wine varietal differentiation [35]. Many other (mono)terpenes also turned out to be important in this sense, while other compound classes exhibited lower *F*-ratios, with the exception of 1-hexanol. Such an outcome was expected to some extent, since terpenes are primary aroma compounds originating from grapes, both as free volatile molecules or released from glycosidic precursors. Their composition and amounts are genetically pre-determined: genetic variation in aroma biosynthesis genes cause differences in terpene concentrations between grapevine varieties. For example, a variant of 1-deoxy-D-xylulose-5-phosphate synthase, a gene responsible for the biosynthesis of terpenoids, causes pronounced increase in terpene concentration in Muscat and Gewürztraminer grapes, which gives wines of these varieties a recognizable floral aroma [4,38,39]. Monoterpenes are generally known to be responsible for varietal aroma of muscats and non-muscat aromatic varieties, such as Gewürtztraminer, Riesling, Müller-Thurgau, etc. [36,40,41], but were also found useful for the differentiation of wines of other, so-called semi-aromatic and neutral grape varieties [41,42,43,44,45]. Márquez, Castro, Natera, and García-Barroso [46] characterized the volatile fraction of Andalusian sweet wines made from Muscat and Pedro Ximenez varieties and, interestingly, also found that α-terpineol was the most powerful differentiator with the highest *F*-ratio, followed closely by linalool and limonene, similar as in this case. 

In this study, the ratios of terpene concentrations in different monovarietal wines varied from compound to compound, but it was generally observed that wines from Škrlet, a relatively unexplored Croatian grape variety, were characterized by the highest concentrations of many important monoterpenes (Table 1), while the concentrations of other monoterpenes were also among the highest in the investigated wines. The concentrations of monoterpenes in Malvazĳa istarska wines were notable and generally in fair agreement with those reported previously for this variety, with linalool followed by geraniol as the most abundant [43,47,48,49]. Malvazija was followed by Pošip wine with intermediate concentrations, while Maraština and especially Kraljevina wines had the lowest terpene concentrations. 

Although the content and composition of terpenes in grapes and wines is principally pre-determined by variety, they are susceptible to modulation in response to many factors, such as viticultural parameters including soil characteristics, exposure to sunlight, water status, defoliation, crop thinning, etc. [34,50], as well as pre-fermentation and fermentation practices and conditions [35,36]. Except the effect of variety, the differences between the investigated monovarietal wines were probably partly caused by different geographical origin (Istria, Dalmatia, continental Croatia), so the effects of variety and location probably acted in synergy. It is indeed known that low temperatures favor the production of aroma compounds in grapes [51], so it is possible that the highest concentration of monoterpenes in Škrlet wines from continental Croatia characterized by lower temperatures was at least partly due to the effect of climate. The same could be deduced for Malvazija wines coming from the northern, somewhat colder part of the Adriatic coast. Conversely, elevated temperatures have potential to reduce the aromatic potential of grapes [52], which is possibly a reason for somewhat lower concentrations of monoterpenes in Dalmatian Pošip and Maraština wines. Kraljevina wines, which had the lowest concentrations of terpenes despite originating from the continental part, could be an exception that confirms the rule. 

C_13_-Norisoprenoids are also secondary metabolites in grapes, present in both aromatic and neutral varieties. They are formed as biodegradation products of carotenoid molecules, such as lutein, β-carotene, violaxanthin, and neoxanthin, via numerous formation mechanisms and intermediates during pre-fermentative steps, fermentation, and aging [53,54]. Four of them, β-damascenone, β-ionone, 1,1,6-trimethyl-1,2-dihydronaphthalene (TDN), and *trans*-1-(2,3,6-trimethylphenyl)buta-1,3-diene (TPB), were commonly found in wine at concentrations surpassing their odor perception thresholds, meaning they can have a direct impact on wine aroma [34]. Especially important is β-damascenone with its pleasant odor reminiscent of honey, dried plum and stewed apple, and a very low perception threshold, which ranks it among the most important wine odorants [37]. β-Ionone, characterized by a threshold of the similar order of magnitude, also significantly contributes to wine aroma with an odor reminiscent of violets, while the contribution of TDN and TPB becomes relevant mostly in aged wines [34]. The concentrations of the majority of C_13_-norisoprenoids were generally higher in Dalmatian Pošip and Maraština, and the lowest in Kraljevina wines, although in particular cases with no statistical significance (Table 1). According to Marais and van Wyk [54] the concentration of β-damascenone is principally dependent on viticultural and winemaking conditions, while variety has less influence. Nevertheless, particular differences were observed: Malvazija wines were found to contain the highest concentration, although not different from that found in Pošip, while Škrlet had the lowest, not different from that found in Maraština wine. Malvazija was also characterized by the lowest concentration of vitispiranes together with Kraljevina wine. Among benzenoids, ethyl cinnamate emerged as a prominent marker of Pošip varietal origin, since it was found in the highest concentration in this wine.

C_6_-alcohols are formed mainly in pre-fermentation vinification steps by degradation of unsaturated fatty acids by the action of enzymes, as well as by liberation from glycosidic precursors. They may have an effect on wine aroma with their so-called green and herbal odors, but luckily have relatively high odor perception thresholds, such as 8000 μg/L for 1-hexanol [37], so only very high concentration can produce negative effects. Certain authors include C_6_-compounds among varietal aromas [16] and their concentrations were found useful in differentiation of particular wines based on variety [43,55]. The highest concentration of 1-hexanol was found in Škrlet, while Kraljevina contained the lowest amount (Table 1). Maraština, and especially Pošip wines were characterized by the highest concentration of unsaturated C_6_-alcohols. It is possible that the mentioned differences were a consequence of different enzymatic potentials and fatty acid precursor loads in grapes of these varieties [55].

Concentrations and the composition of fermentation aroma compounds are mainly affected by fermentation conditions, but may also be influenced by grape composition [56]. Many studies proved that the composition of volatile compounds formed in fermentation can be useful in differentiating wines of mostly neutral varieties equally or even more successful than by using, e.g., monoterpene concentrations [11,14,20,29]. This is more characteristic for C_6_–C_10_ fatty acids and the corresponding ethyl esters which, in contrast to acetates, are more dependent on the concentration of precursors and therefore on variety and conditions in vineyard, and less on the activity of yeast [57]. The average concentration of 2-phenylethanol was higher than the corresponding odor perception threshold of 10,000 μg/L in all the studied monovarietal wines, meaning this alcohol contributed significantly with its odor reminiscent of roses [37]. Pošip and Maraština had approximately 50% higher concentration of 2-phenylethanol in relation to the other investigated wines (Table 1). The concentrations of major volatile fatty acids (C_6_–C_10_) surpassed the corresponding odor perception thresholds of 420, 500, and 1000 μg/L, respectively [58], in all the investigated wines. Fatty acid production is determined in part by the initial composition of must [59] and therefore possibly by varietal origin. Malvazija istarska wines stood out with low concentrations of decanoic and octanoic acid. Among esters, Pošip was clearly differentiated from the other monovarietal wines by the highest concentration of 2-phenethyl acetate, which could have been related to the higher concentration of its precursor 2-phenylethanol found in this wine. However, it was stated previously that precursor concentrations do not significantly determine the concentrations of acetate esters formed by *Saccharomyces cerevisiae*, with the expression of alcohol acetyl transferase gene in yeast as a limiting factor [60]. Concentration of 2-phenethyl acetate in all the investigated wines was higher than the corresponding threshold of 250 μg/L [37], suggesting its floral odor participated in the aroma of all the wines. The major ethyl and acetate esters are among the most important volatile compounds for the fresh fruity aroma of young white wines to which they significantly contribute by commonly multiply surpassing their rather low odor perception thresholds, such as 30 μg/L for isoamyl acetate, 20 μg/L for ethyl butyrate, 5 μg/L for ethyl hexanoate, and 2 μg/L for ethyl octanoate [37]. The highest concentration of linear middle-chain ethyl esters and acetates other than 2-phenylethyl acetate, although in some cases without statistical significance, was noted in Malvazija istarska wines. Pošip was also relatively abundant in these esters, except for ethyl hexanoate which was found in the lowest concentration in this and in Maraština wines. Although hexanoic acid is mainly formed in fermentation, grapes also contain non-negligible concentration. This means that the concentration of ethyl hexanoate in wine is probably partly influenced by the concentration of its precursor, hexanoic acid, in grapes [4], so the lower concentration of ethyl hexanoate in Pošip and Maraština could have been influenced by a genotype.

### 3.2. GC×GC-TOF-MS

A characteristic HS-SPME/GC×GC-TOF-MS analysis 2D chromatogram of volatile compounds in Malvazija istarska wine is shown in Figure S1. It can be seen that many compounds which were separated by the second dimension column had the same retention times on the first, meaning these compounds would not be adequately separated by the conventional GC-MS. The average concentrations of volatile compounds (tentatively) identified in the investigated wines after GC×GC-TOF-MS analysis are reported in Table 2, while the concentrations found in each of the investigated wines are reported in Table S3. Compounds were grouped according to chemical class, and sorted within each class in order of decreasing *F*-ratio determined by one-way ANOVA. Three hundred and seventeen (317) volatile aroma compounds were identified, including 53 terpenes, 10 norisoprenoids, 50 benzenoids, 5 hydrocarbons, 7 aldehydes, 24 ketones, 32 alcohols, 16 acids, 73 esters, 5 volatile phenols, 17 furanoids and lactones, 19 sulfur containing compounds, and 6 other compounds. GC×GC-TOF-MS exhibited superior peak annotation ability than GC-MS which enabled the identification of a much larger number of compounds, as a consequence of higher separation efficiency, enhanced sensitivity, and clearer mass spectra allowed by separation on two different phases [23]. Other factors which could have affected the differences between the results obtained by the two techniques/methods were the absolute sensitivity of the analyzers, SPME conditions (sample volume and dilution, duration and temperature of extraction, fiber length, etc.), and others. To our knowledge, with 350 compounds identified by GC-MS and GC×GC-TOF-MS combined, this study reported one of the most detailed volatile aroma profiles in wine to date. It has to be noted that for particular compounds which were analyzed and reported by both the techniques applied the obtained absolute concentrations differed due to different quantification methods used: quantitative analysis with the use of standards solutions and calibration curves in GC-MS, and semi-quantification relative to internal standard 1-octanol concentration, assuming a response factor equal to one, in GC×GC-TOF-MS analysis, respectively. The concentrations of many volatile compounds were found to be significantly different between wines (161), but relatively few were found to be exclusive markers of particular variety.

In order to compare the techniques applied, the GC×GC-TOF-MS results for the major monoterpenols and some other compounds already quantified by GC-MS and reported in Table 1 were also reported in Table 2. It was observed that the results, in relative terms, were mostly in fair agreement. α-Terpineol was confirmed as a monoterpene and a volatile aroma compound in general with the highest discriminative power, with an *F*-ratio even higher than that obtained after GC-MS data elaboration. α-Terpineol was followed by limonene and linalool, as well as some other monoterpenes which were also among the most potent volatiles according to this criterion as determined by GC-MS, such as nerol, ho-trienol, 4-terpineol, and *trans*-β-ocimene. On the other hand, some discrepancies were observed; for example, in the case of geraniol, α-terpinolene, and geranyl ethyl ether, with a high *F*-ratio obtained by GC×GC-TOF-MS and a relatively low *F*-ratio obtained by GC-MS data elaboration. The opposite was observed for citronellol. It is possible that the discrepancies observed derived from the co-elution of the mentioned monoterpenes with particular unidentified compounds having mass spectra with ions of equal mass to those used for quantification of terpenes during GC-MS analysis, although strict measures have been taken to ensure the quality of the results. 

Similar as in the case of GC-MS results (Table 1), Škrlet wines were the most abundant in monoterpenes, followed by Malvazija istarska, then Pošip, and finally Maraština and Kraljevina wines with the lowest concentrations (Table 2). Only a few exceptions were noted: Škrlet wines contained the lowest concentration of β-calacorene, while Malvazija wine was deficient in *cis*-Z-α-bisabolene epoxide. Although Kraljevina wine was generally poor in terpenes, several sesquiterpenes, such as cadalene, β-calacorene, and especially tentatively identified γ-dehydro-ar-himachalene, emerged as potential markers of the varietal origin of this wine. 

All the other classes of compounds were confirmed to be far less efficient in differentiating the investigated monovarietal wines than terpenes, with few exceptions. The number of C_13_-norisoprenoids identified by the two techniques applied was similar, but their identities differed in most cases. The relative results for β-damascenone obtained by GC-MS and GC×GC-TOF-MS were in a fair agreement, with the highest concentration found in Malvazija istarska and the lowest in Škrlet wines (Table 2). A similar degree of correspondence between GC-MS and GC×GC-TOF-MS results and the corresponding *F*-ratios was observed for a vitispirane isomer. Kraljevina wines contained the highest concentration of tentatively identified 1,2-dihydro-1,4,6-trimethylnaphthalene.

Superiority of GC×GC-TOF-MS over GC-MS in terms of compound separation and identification was demonstrated well in the analysis of benzenoids, with a much larger number of compounds identified by the former technique. Several benzenoids were found to be relatively efficient discriminators between monovarietal wines, and some of them were exclusive differentiators for particular varieties. High ethyl benzene concentration was specific for Pošip, while 1,1′-oxybisbenzene was most abundant in Malvazija istarska wines, in both cases supported by rather high *F*-ratios. In addition to the highest concentration of 1,1′-oxybisbenzene, Malvazija istarska wine was characterized by most varietal markers among benzenoids, including octylbenzene, a non-identified benzenoid, azulene, 2-methylnaphthalene, and methyl 2-(benzyloxy)propanoate. Pošip was characterized by the highest ethyl benzene and *trans*-edulan concentration, Kraljevina was the most abundant in 6-[1-(hydroxymethyl)vinyl]-4,8a-dimethyl-1,2,4a,5,6,7,8,8a-octahydro-2-naphthalenol, while Škrlet wine was the richest in *m*-methoxyanisole and α,α-dimethylbenzenemethanol (Table 2). 

No significant differences were found between the concentrations of hydrocarbons, while aldehydes also turned out to be poor varietal differentiators, with significant differences found only for decanal (Table 2). On the other hand, several ketones were found useful for this purpose: the highest concentration of 2-undecanone and 3-undecanone was specific for Malvazija istarska, 1,4,7,10,13-pentaoxacyclononadecane-14,19-dione and cyclohexylideneacetone were characteristic for Škrlet, while the lowest concentration of isophorone was found in Maraština wines. 

4-Methyl-1-heptanol was the most useful among alcohols in differentiating monovarietal wines with a rather high *F*-ratio (Table 2). It was found in the highest concentration in Škrlet, followed by Malvazija istarska wines, while the other wines contained lower concentrations. The results for *cis*-3-hexen-1-ol were in accordance with those obtained by GC-MS, with the highest concentration found in Dalmatian Pošip and Maraština wines. 3-Octanol and 1-octen-3-ol were exclusive markers for Pošip, 2-decanol for Škrlet, while the lowest concentration of an isomer of 2-penten-1-ol was characteristic for Kraljevina wine. *F*-ratios determined for fatty acids were relatively low and significant differences were found only for five of them. 

A very large number of minor esters was identified by GC×GC-TOF-MS analysis (Table 2). In accordance with the GC-MS data, the concentrations of the majority of esters of aliphatic higher alcohols and fatty acids were the highest in Malvazija istarska wines. Despite the thesis that precursor concentrations do not significantly determine the concentrations of acetate esters formed by *Saccharomyces cerevisiae* [60], the highest concentration of *cis*-3-hexen-1-yl acetate corresponded to the highest concentration of its precursor, *cis*-3-hexen-1-ol, found in Pošip wine. Pošip wine was the most abundant in particular esters of ethanol and hydroxyl keto acids, such as diethyl glutarate and ethyl pyruvate. Although without a statistically significant difference, the concentrations of the related esters, such as ethyl lactate and diethyl succinate, determined by GC-MS, also had a tendency to be higher in Pošip wines. 

Pošip wines contained the highest concentration of volatile phenols, such as 2-methoxyphenol and 4-vinylguaiacol. Significant differences were found for particular furanoids and lactones. A number of sulfur containing compounds was identified, with many of them found in the highest concentration in Pošip wines, some with relatively high *F*-ratios, such as methional. Kraljevina and Škrlet wines were generally the least abundant in these compounds (Table 2).

### 3.3. Multivariate Statistical Analysis

**Figure 1:**
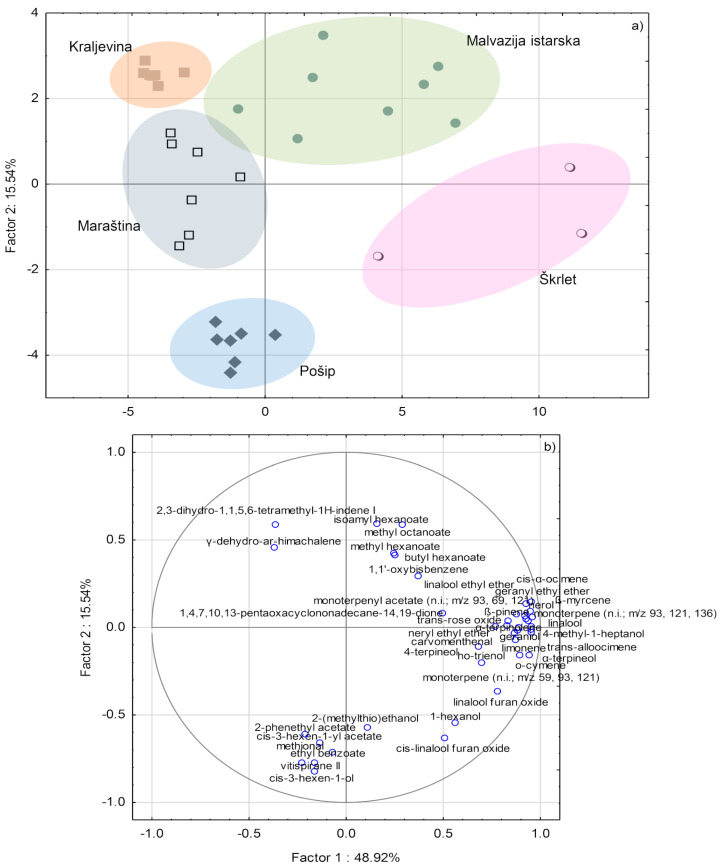
(**a**) Separation of Croatian monovarietal wines according to variety in two-dimensional space defined by the first two principal components, PC1 and PC2; (**b**) Factor loadings of selected variables (40 volatile aroma compounds with the highest *F*-ratios), as determined by gas chromatography mass spectrometry (GC-MS) and two-dimensional gas chromatography with time-of-flight mass spectrometry (GC×GC-TOF-MS) analysis, on PC1 and PC2.

PCA allowed a good separation of the investigated monovarietal wines according to variety when applied on a dataset reduced to 40 variables with the highest *F*-values, obtained by both GC-MS and GC×GC-TOF-MS analysis. Monovarietal wines were clearly separated from each other in two-dimensional space despite a relatively high number of varieties (Figure 1). Škrlet wine was clearly differentiated from the others along the direction of PC1 and was characterized by higher amounts of terpenes. A part of Malvazija istarska wines also gravitated towards higher positive PC1 values, but the wines of this variety were also separated from the others along the direction of PC2, mostly due to higher concentrations of particular esters with positive PC2 values. Volatile aroma compounds located in the second quadrant of Cartesian system with negative PC1 and positive PC2 coordinates, 2,3-dihydro-1,1,5,6-tetramethyl-1H-indene and γ-dehydro-ar-himachalene, contributed most to the separation of Kraljevina wines, while the location of Pošip wines was obviously conditioned by the loadings of *cis*-3-hexen-1-ol, vitispirane II, ethyl benzoate, methional, *cis*-3-hexen-1-yl acetate, 2-phenethyl acetate, and 2-(methylthio)ethanol. Maraština wines were apparently not linked to any particular compound class, probably due to lower concentrations of the 40 volatile compounds used for PCA.

**Figure 2:**
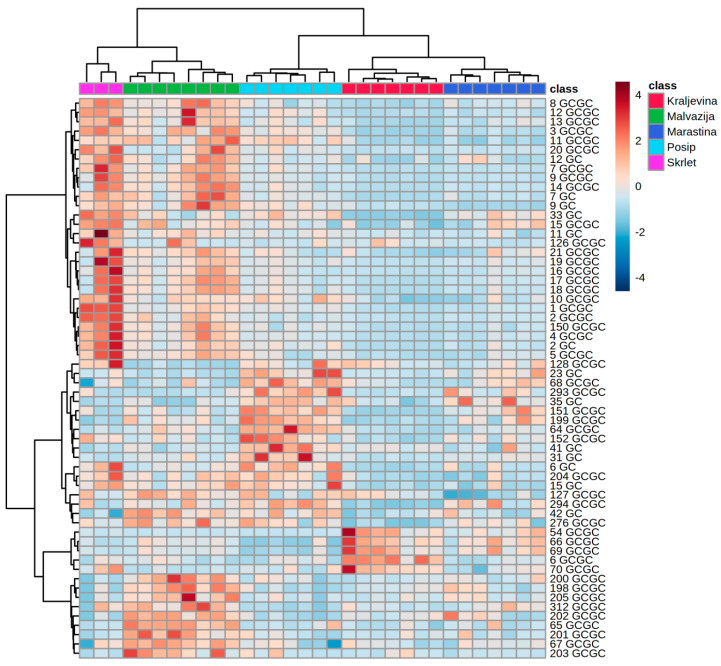
Hierarchical clustering analysis performed using volatile aroma compound profiles of Croatian monovarietal wines obtained by GC-MS and GC×GC-TOF-MS analysis. The heatmap was generated using 60 most significant compounds (the highest *F*-ratios). The rows in the heatmap represent compounds and the columns indicate samples. Compounds are designated by numbers which correspond to those in Table 1 (GC, i.e., GC-MS) or in Table 2 (GCGC, i.e., GC×GC-TOF-MS). The colors of heatmap cells indicate the abundance of compounds across different samples. The color gradient, ranging from dark blue through white to dark red, represents low, middle, and high abundance of a compound.

Hierarchical clustering analysis according to variety, performed using the amounts of the 60 volatile aroma compounds with the highest *F*-ratio, confirmed that each monovarietal wine had a distinct volatile profile (Figure 2). Most of the conclusions were similar to those obtained by the PCA. Škrlet and Malvazija Istarska wines were clearly separated from each other mostly due to higher concentrations of particular esters in the latter, but were clustered together by high terpene concentrations. The generated heatmap probably offered the clearest insight into the intra-varietal diversity of particular wines, especially Malvazija with two evident clusters with different terpene content. Pošip formed a distinct cluster mostly due to high concentrations of particular compounds from several classes, some of them already mentioned in the PCA, including vitispirane II, *trans*-edulan, methional, 2-phenyletahnol, *cis*-3-hexen-1-ol and its acetate, ethyl benzoate, 2-heptanol, 2-phenethyl acetate, ethyl cinnamate, and others. Kraljevina wines were clearly the least abundant in the majority of the 60 pre-selected compounds, except for γ-dehydro-ar-himachalene, 1,2-dihydro-1,4,6-trimethylnaphthalene and particular benzenoids, which were confirmed as its markers.

**Figure 3:**
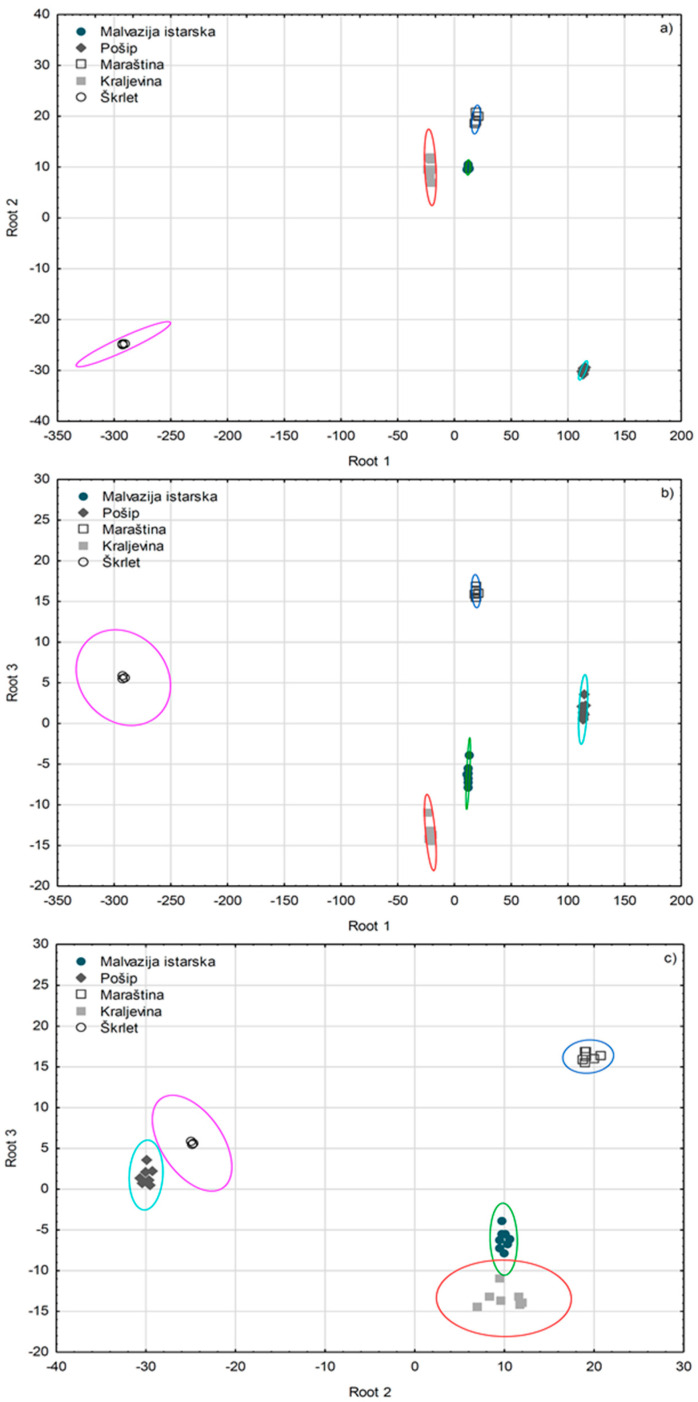
Separation of Croatian monovarietal wines according to variety defined by the first three discriminant functions (roots) obtained by forward stepwise discriminant analysis (SLDA) on the basis of volatile aroma compound composition determined by GC-MS and GC×GC-TOF-MS analysis. (**a**) root 1 vs root 2; (**b**) root 1 vs root 3; (**c**) root 2 vs root 3.

SLDA was applied on a dataset reduced to 60 most significant volatile aroma compounds according to *F*-ratio from both GC-MS and GC×GC-TOF-MS original datasets. All the monovarietal wines were classified correctly according to variety by this model, and 24 most significant variables were extracted (Figure 3), with rather high squared Mahalanobis distances from group centroids. A 100% correct classification was obtained after including only seven variables. α-Terpineol was confirmed once again as the most powerful varietal marker, since the SLDA model classified correctly 68.75% of all the wines and 100.00% of Škrlet wines by using only this variable. After including β-pinene and ethyl benzoate the total percentage of correctly classified wines increased to 93.75%. For achieving a 100.00% correct classification, 1,1′-oxybisbenzene, γ-dehydro-ar-himachalene, vitispirane II, and 2,6,10,10-tetramethyl-1-oxaspiro[4.5]deca-3,6-diene were included in the SLDA model. The following 17 volatile aroma compounds were also included: 2-phenethyl acetate, isophorone, monoterpenyl acetate (n.i.; *m/z* 93, 69, 121), 2,3-dihydro-1,1,5,6-tetramethyl-1H-indene II, *cis*-3-hexen-1-ol, methyl hexanoate, *trans*-rose oxide, methyl decanoate, *cis*-3-hexen-1-yl acetate, monoterpene (n.i.; *m/z* 93, 69, 41), β-myrcene, limonene, 3-methyl-2(5H)-furanone, 2-phenylethanol, 1,2-dihydro-1,4,6-trimethylnaphthalene, nerol, and nerol oxide.

Apparently, SLDA has extracted volatile aroma compounds which were most useful for the differentiation of the five investigated monovarietal wines between each other, which only partly coincided with the compounds with the highest *F*-ratios obtained by ANOVA. Monoterpenes had a key role again, especially α-terpineol. The ability of the SLDA model to predict a correct variety was checked by “leave-one-out” cross-validation, where each wine sample was excluded and classified by the functions derived from all the other wine samples. The correct prediction rate achieved was 100.00%.

**Figure 4:**
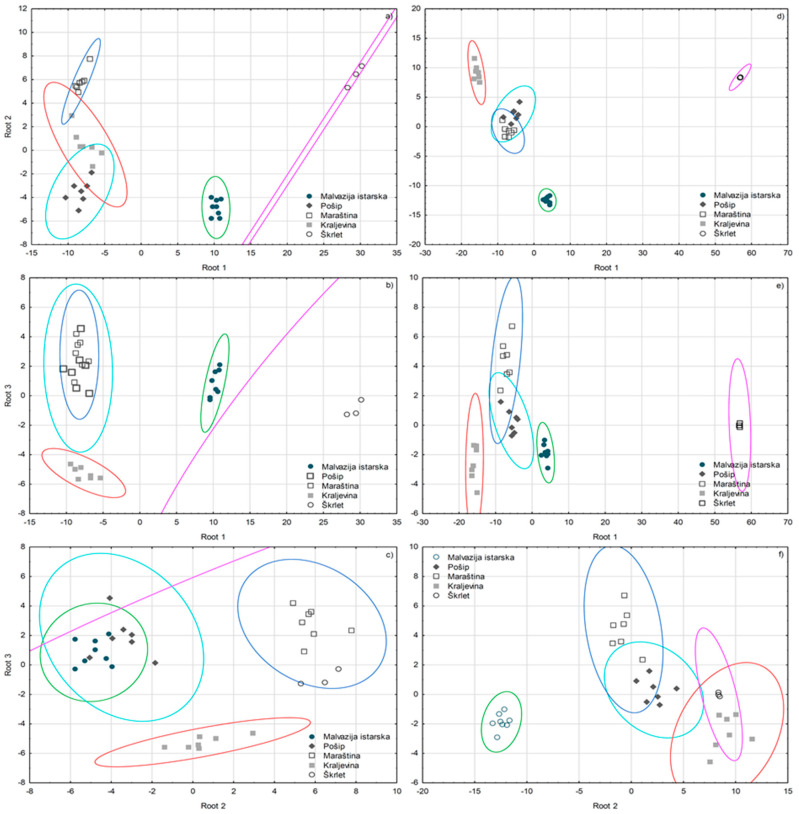
Separation of Croatian monovarietal wines according to variety defined by the first three discriminant functions (roots) obtained by forward stepwise discriminant analysis (SLDA) on the basis of volatile terpene composition determined by GC-MS (**a**–**c**) and GC×GC-TOF-MS (**d**–**f**) analysis.

To compare the usefulness of the information contained in the composition of terpenes alone obtained by GC-MS and GC×GC-TOF-MS analysis for differentiating monovarietal wines, SLDA was applied separately on the two datasets containing 20 and 31 terpenes, respectively, found significant by ANOVA. Both GC-MS and GC×GC-TOF-MS dataset based models succeeded in achieving 100.00% correct classification (Figure 4). α-Terpineol was again confirmed as a key differentiator, since both models included it as the first, which classified correctly 59.38% and 68.75% monovarietal wines, respectively. For achieving 100.00% correct classification, the GC-MS model further included *trans*-ocimene, *cis*-linalool furan oxide, β-pinene, citronellol, *trans*-nerolidol, ho-trienol, *trans*-rose oxide, and limonene, while the GC×GC-TOF-MS model extracted γ-dehydro-ar-himachalene, ho-trienol, nerol, *o*-cymene, isogeraniol, a non-identified sesquiterpene (n.i.; *m/z* 119, 93, 69), neryl ethyl ether, and *cis*-α-ocimene. The classification efficacy of the models was improved by including further eight and nine terpenes, respectively. The GC×GC-TOF-MS model exhibited a superior efficacy judging from the degree of the overlapping of the corresponding 95% confidence areas, as well as higher squared Mahalanobis distances on the average, especially for Škrlet wines.

The volatile aroma compounds which were found to be most useful for the differentiation of the investigated wines in this study were only partly in accordance with the ones highlighted in previous studies which applied a similar multivariate statistical approach. For example, Welke et al. [29] characterized and differentiated wines from Chardonnay, Sauvignon Blanc, Pinot Noir, Merlot, and Cabernet Sauvignon based on volatile aroma composition obtained by GC×GC-TOF-MS analysis and extracted the following 12 volatile compounds as the most useful for their differentiation: diethyl succinate, 2,3-butanediol, nerol, 3-penten-2-one, diethyl malonate, β-santalol, ethyl 9-decenoate, alcohol-C_9_, 4-carene, tetrahydro-2(2H)-pyranone, dihydro-2(3H)-thiophenone, and 3-methyl-2(5H)-furanone. It is probable that the main reason for such discrepancy between this and the study from Welke et al. [29] was the fact that the mentioned authors mutually compared wines from white and red varieties, which greatly differ with respect to the production technology, which, besides variety, certainly greatly contributed to the differences between wines. Welke et al. [29] also obtained a SLDA model that differentiated wines according to variety with a 100% correct recognition ability, while some other authors who applied conventional GC-MS for the same purpose, such as Zhang et al. [61] and Câmara, Alves and Marques [14], did not succeed completely. Fabani, Ravera, and Wunderlin [15] obtained a 100% correct discrimination among Syrah, Malbec, and Bonarda red wines by the application of SLDA on GC-MS data with ethyl hexanoate, ethyl octanoate, 1-hexanol, benzyl alcohol, and isoamyl acetate as the most useful differentiators. Terpenes were not analyzed. Ziółkowska, Wąsowicz, and Jeleń [19] obtained a relatively good differentiation of red wines, with the ability of the LDA model to correctly classify and predict their varietal origin based on HS-SPME/GC-MS data of 95%, while the model built for white wines was not that successful. The compounds most useful for the differentiation of white wines (Chardonnay, Sauvignon Blanc, and Muscat) were isoamyl acetate, furfural, ethyl octanoate, ethyl decanoate, and ethyl dodecanoate, while red wines (Cabernet Sauvignon and Merlot) were differentiated mainly by 1-hexanol, ethyl decanoate, and 2-phenylethanol. It should be noted that the samples of the same variety were collected across several countries, which was certainly a factor that introduced large variability.

## 4. Conclusions

HS-SPME/GC×GC-TOF-MS analysis, alone or combined with conventional HS-SPME/GC-MS, was shown to be an excellent analytical tool for differentiation of wines according to variety based on volatile aroma compound composition. It has also been proven that the additional separation efficiency enabled by the second chromatographic column in GC×GC-TOF-MS analysis was crucial for the separation and identification of a very large number of volatile compounds, which would otherwise remain undetected by conventional GC-MS. This feature provided additional in-depth volatile profile information which was exploited for highly efficient white wine varietal differentiation. Such an outcome can be considered even more successful knowing that the number of varieties was relatively high while that of wine samples of each variety was relatively small, and that the investigated wines were characterized by high intra-varietal heterogeneity in terms of micro-locations and grape cultivation and winemaking parameters. The results of this study confirmed the unmatched power of monoterpenes to discriminate wines according to variety, which was robust enough to be captured by uni- and multivariate statistics based on both GC-MS and GC×GC-TOF-MS analysis data separately.

## Figures and Tables

**Table 1:** Concentrations (μg/L) of volatile aroma compounds found in Croatian monovarietal wines after headspace solid-phase microextraction followed by gas chromatography-mass spectrometry (HS-SPME/GC–MS) sorted by compound class and descending Fisher *F*-ratio.

No.	Volatile Compounds	t_R_	ID	LRI_exp_	LRI_lit_	*F*-Ratio	Variety				
		(min:s)					MI	PO	MA	KR	SK
	*Terpenes*										
1	α-Terpineol	39:59	S, MS, LRI	1684	1684	35.07	15.65 ± 7.04 ^b^	10.92 ± 3.16 ^bc^	5.50 ± 2.10 ^cd^	1.98 ± 0.89 ^d^	40.49 ± 11.05 ^a^
2	Monoterpene (n.i.; *m/z* 59, 93, 121)	28:29	MS	1441	-	27.68	1.03 ± 0.50 ^b^	0.64 ± 0.24 ^bc^	0.27 ± 0.24 ^cd^	0.02 ± 0.04 ^d^	2.79 ± 1.01 ^a^
3	Linalool	33:10	S, MS, LRI	1542	1542	24.71	68.00 ± 27.76 ^b^	38.17 ± 8.05 ^c^	18.52 ± 8.28 ^d^	7.64 ± 2.56 ^d^	90.75 ± 15.48 ^a^
4	Limonene	15:17	MS, LRI	1191	1196	18.91	1.33 ± 0.68 ^b^	0.98 ± 0.28 ^b^	0.19 ± 0.05 ^c^	0.36 ± 0.11 ^c^	2.66 ± 1.01 ^a^
5	Nerol	44:35	S, MS, LRI	1791	1791	16.31	13.38 ± 6.72 ^a^	6.49 ± 1.96 ^b^	4.55 ± 1.58 ^bc^	1.06 ± 0.46 ^c^	17.36 ± 3.74 ^a^
6	*cis*-Linalool furan oxide	29:24	MS, LRI	1464	1464	12.57	0.08 ± 0.03 ^b^	0.18 ± 0.06 ^a^	0.06 ± 0.05 ^b^	0.02 ± 0.01 ^b^	0.20 ± 0.11 ^a^
7	Monoterpenyl acetate (n.i.; *m/z* 93, 69, 121)	21:55	MS	1302	-	12.50	3.12 ± 1.85 ^a^	1.11 ± 0.27 ^b^	0.59 ± 0.36 ^b^	0.12 ± 0.06 ^b^	3.16 ± 0.84 ^a^
8	4-Terpineol	35:37	MS, LRI	1594	1596	11.60	0.24 ± 0.10 ^b^	0.24 ± 0.06 ^b^	0.23 ± 0.11 ^b^	0.11 ± 0.03 ^c^	0.51 ± 0.08 ^a^
9	β-Pinene	13:45	MS, LRI	1146	1145	11.46	4.40 ± 2.41 ^a^	2.43 ± 0.75 ^bc^	0.40 ± 0.15 ^d^	1.16 ± 0.37 ^cd^	4.17 ± 1.06 ^ab^
10	Ho-Trienol	36:02	MS, LRI	1601	1601	10.44	7.45 ± 3.95 ^a^	6.95 ± 2.01 ^ab^	1.71 ± 1.14 ^c^	1.60 ± 0.76 ^c^	4.18 ± 0.79 ^bc^
11	*trans*-Rose oxide	23:23	MS, LRI	1352	1341	10.32	0.27 ± 0.08 ^b^	0.21 ± 0.04 ^bc^	0.15 ± 0.07 ^c^	0.16 ± 0.03 bc	0.59 ± 0.34 ^a^
12	Monoterpene (n.i.; *m/z* 93, 69, 41)	29:56	MS	1476	-	8.51	0.49 ± 0.28 ^b^	0.25 ± 0.07 ^c^	0.24 ± 0.17 ^c^	0.12 ± 0.13 c	0.77 ± 0.24 ^a^
13	*trans*-Ocimene	18:03	MS, LRI	1252	1250	8.45	1.58 ± 0.92 ^a^	1.27 ± 0.41 ^ab^	0.17 ± 0.07 ^c^	0.55 ± 0.18 ^bc^	1.30 ± 0.50 ^ab^
14	Citronellol	43:11	S, MS, LRI	1758	1758	8.34	5.02 ± 0.61 ^a^	5.09 ± 0.69 ^a^	5.30 ± 1.78 ^a^	2.56 ± 0.30 ^b^	5.60 ± 1.75 ^a^
15	Nerol oxide	29:18	MS, LRI	1459	1464	7.01	3.04 ± 1.12 ^a^	3.74 ± 1.82 ^a^	1.35 ± 1.17 ^b^	1.11 ± 0.40 ^b^	4.11 ± 1.44 ^a^
16	Geranyl acetone	47:01	MS, LRI	1845	1845	5.38	2.93 ± 0.58 ^b^	3.58 ± 0.99 ^b^	7.58 ± 4.80 ^a^	2.64 ± 0.39 ^b^	2.55 ± 1.14 ^b^
17	*trans*-Linalool pyran oxide	41:49	MS, LRI	1726	1752	4.85	0.08 ± 0.02 ^b^	0.13 ± 0.05 ^a^	0.07 ± 0.05 ^b^	0.04 ± 0.03 ^b^	0.06 ± 0.02 ^b^
18	*trans*-Nerolidol	54:39	MS, LRI	2031	2031	4.61	2.89 ± 0.50 ^a^	3.17 ± 0.59 ^a^	2.66 ± 1.58 ^ab^	1.59 ± 0.22 ^b^	1.53 ± 0.35 ^b^
19	Monoterpene (n.i.; *m/z* 121, 93, 136)	31:30	MS	1509	-	3.09	2.45 ± 0.49 ^a^	2.41 ± 0.56 ^a^	2.11 ± 0.73 ^a^	1.11 ± 0.16 ^b^	2.88 ± 2.79 ^a^
20	Geraniol	46:35	S, MS, LRI	1838	1838	2.93	40.64 ± 21.59 ^ab^	24.23 ± 8.96 ^ab^	39.96 ± 48.27 ^ab^	2.73 ± 1.56 ^b^	46.19 ± 10.53 ^ab^
21	Geranyl ethyl ether	31:54	MS, LRI	1511	1499	2.69	0.53 ± 0.33	0.86 ± 0.97	1.08 ± 0.84	0.05 ± 0.02	0.82 ± 0.25
22	α-Terpinolene	19:34	MS, LRI	1287	1281	2.32	0.49 ± 0.29	0.73 ± 0.92	0.07 ± 0.04	0.14 ± 0.07	0.33 ± 0.26
	*C_13_-norisoprenoids*										
23	Vitispirane II	31:16	MS, LRI	1523	1529	9.85	0.07 ± 0.02 ^c^	0.34 ± 0.16 ^a^	0.20 ± 0.10 ^b^	0.09 ± 0.01 ^c^	0.14 ± 0.06 ^bc^
24	β-Damascenone	45:26	MS, LRI	1809	1809	7.09	3.52 ± 0.69 ^a^	2.81 ± 1.42 ^ab^	1.99 ± 0.58 ^bc^	2.28 ± 0.25 ^b^	0.89 ± 0.29 ^c^
25	Actinidol I	49:55	MS, LRI	1914	1914	5.59	0.12 ± 0.05 ^a^	0.16 ± 0.06 ^a^	0.13 ± 0.07 ^a^	0.04 ± 0.01 ^b^	0.09 ± 0.03 ^ab^
26	Actinidol II	50:27	MS, LRI	1927	1927	5.10	0.20 ± 0.08 ^a^	0.23 ± 0.07 ^a^	0.23 ± 0.10 ^a^	0.08 ± 0.01 ^b^	0.16 ± 0.04 ^ab^
27	Vitispirane I	32:08	MS, LRI	1521	1526	5.03	0.09 ± 0.04 ^c^	0.46 ± 0.24 ^a^	0.33 ± 0.24 ^ab^	0.19 ± 0.05 ^bc^	0.32 ± 0.18 ^abc^
28	β-Ionone	50:17	S, MS, LRI	1923	1923	3.89	0.06 ± 0.01 ^ab^	0.05 ± 0.01 ^b^	0.07 ± 0.01 ^a^	0.05 ± 0.01 ^b^	0.07 ± 0.01 ^a^
29	Actinidol ethyl ether I	40:25	MS, LRI	1690	1690	3.37	0.25 ± 0.12 ^bc^	0.43 ± 0.24 ^a^	0.34 ± 0.25 ^ab^	0.11 ± 0.02 ^c^	0.24 ± 0.06 ^bc^
30	Actinidol ethyl ether II	41:49	MS, LRI	1723	1723	2.76	0.15 ± 0.07 ^ab^	0.25 ± 0.16 ^a^	0.20 ± 0.15 ^a^	0.06 ± 0.01 ^b^	0.15 ± 0.04 ^ab^
	*Benzenoids*										
31	Ethyl cinnamate	57:33	S, MS, LRI	2111	2122	6.96	0.41 ± 0.19 ^b^	1.16 ± 0.78 ^a^	0.39 ± 0.08 ^b^	0.21 ± 0.10 ^b^	0.16 ± 0.10 ^b^
32	Benzaldehyde	31:26	S, MS, LRI	1508	1509	0.84	1.66 ± 1.25	3.48 ± 5.40	1.17 ± 0.54	2.56 ± 0.81	3.11 ± 1.57
	*Alcohols*										
33	1-Hexanol	23:35	S, MS, LRI	1356	1357	25.56	792.14 ± 264.44 ^b^	949.93 ± 179.86 ^b^	859.15 ± 171.18 ^b^	321.89 ± 32.90 ^c^	1544.09 ± 146.31 ^a^
34	*cis*-3-Hexen-1-ol	25:03	S, MS, LRI	1379	1379	12.73	77.49 ± 40.64 ^c^	299.33 ± 113.23 ^a^	193.20 ± 123.23 ^b^	26.16 ± 4.63 ^c^	54.67 ± 23.77 ^c^
35	2-Phenylethanol	48:52	S, MS, LRI	1891	1893	7.16	20,047.0 ± 4767.1 ^b^	33,176.1 ± 4679.3 ^a^	32,117.2 ± 10,870.7 ^a^	20,712.5 ± 6134.8 ^b^	17,665.9 ± 1061.0 ^b^
36	*trans*-3-Hexen-1-ol	24:03	S, MS, LRI	1361	1361	1.73	61.38 ± 24.09	45.64 ± 17.99	46.57 ± 10.28	43.09 ± 10.26	63.87 ± 22.80
	*Acids*										
37	Decanoic acid	62:49	S, MS, LRI	2257	2258	5.05	646.02 ± 179.70 ^b^	1627.60 ± 659.33 ^a^	1062.71 ± 505.33 ^b^	994.33 ± 67.19 ^b^	1090.36 ± 494.95 ^ab^
38	Octanoic acid	54:56	S, MS, LRI	2043	2042	4.03	4294.07 ± 796.78 ^b^	6239.74 ± 1532.91 ^a^	5147.23 ± 1562.12 ^ab^	6219.42 ± 455.69 ^a^	6359.73 ± 1152.33 ^a^
39	Hexanoic acid	46:10	S, MS, LRI	1830	1828	3.05	5715.09 ± 552.13 ^ab^	5184.65 ± 722.46 ^b^	5284.54 ± 1710.50 ^b^	6487.89 ± 603.01 ^a^	7025.45 ± 1103.35 ^a^
40	Butyric acid	36:28	S, MS, LRI	1612	1612	0.54	1766.10 ± 323.75	1607.09 ± 231.34	1685.41 ± 407.86	1581.53 ± 184.63	1788.32 ± 346.09
	*Esters*										
41	2-Phenethyl acetate	45:03	S, MS, LRI	1803	1801	9.02	2230.06 ± 481.79 ^b^	4731.20 ± 1467.85 ^a^	2359.08 ± 1289.62 ^b^	2579.92 ± 287.25 ^b^	1750.70 ± 284.91 ^b^
42	Ethyl octanoate	28:06	S, MS, LRI	1435	1435	8.88	1211.04 ± 239.22 ^a^	1086.51 ± 223.88 ^a^	817.08 ± 231.10 ^b^	701.64 ± 160.66 ^b^	544.02 ± 243.59 ^b^
43	Ethyl hexanoate	17:35	S, MS, LRI	1236	1236	6.80	721.60 ± 172.38 ^a^	379.34 ± 86.89 ^c^	463.42 ± 153.50 ^bc^	580.60 ± 120.60 ^ab^	474.95 ± 108.08 ^bc^
44	Hexyl acetate	19:26	S, MS, LRI	1272	1272	6.10	216.64 ± 52.04 ^a^	204.45 ± 73.60 ^a^	123.25 ± 54.35 ^b^	107.91 ± 34.65 ^b^	207.09 ± 40.86 ^a^
45	Ethyl decanoate	37:43	S, MS, LRI	1637	1638	5.61	302.58 ± 46.92 ^a^	279.95 ± 69.24 ab	179.10 ± 81.94 ^c^	220.08 ± 29.45 ^bc^	199.26 ± 29.89 ^bc^
46	Isoamyl acetate	12:29	S, MS, LRI	1120	1122	3.97	3299.12 ± 1092.74 ^a^	3321.37 ± 1674.71 ^a^	1460.92 ± 566.57 ^b^	2397.45 ± 774.95 ^ab^	1879.81 ± 562.20 ^ab^
47	Ethyl butyrate	09:27	S, MS, LRI	1030	1030	3.09	456.83 ± 69.21 ^a^	415.44 ± 50.58 ^ab^	363.29 ± 80.70 ^b^	367.30 ± 47.60 ^b^	350.99 ± 74.33 ^b^
48	Ethyl 3-methylbutyrate	10:31	S, MS, LRI	1065	1065	2.58	8.51 ± 1.98	14.79 ± 5.57	12.86 ± 5.26	11.34 ± 4.39	8.39 ± 0.88
49	Ethyl 2-methylbutyrate	10:00	S, MS, LRI	1049	1049	2.17	4.19 ± 1.13	6.57 ± 2.31	6.57 ± 2.70	6.25 ± 2.48	4.01 ± 0.62
50	Diethyl succinate	39:04	S, MS, LRI	1667	1669	1.53	1634.59 ± 398.33	1917.40 ± 1362.67	1665.03 ± 858.64	997.49 ± 290.90	1064.55 ± 106.44
51	Ethyl lactate	22:56	S, MS, LRI	1341	1341	0.58	25,943.4 ± 13,586.8	45,815.9 ± 55,981.7	34,462.1 ± 16,552.6	25,359.5 ± 12,701.8	32654.3 ± 7282.3

ID—identification of compounds; S—retention time and mass spectrum consistent with that of the pure standard and with NIST05 mass spectra electronic library; LRI—linear retention index consistent with that found in literature; MS—mass spectra consistent with that from NIST05 mass spectra electronic library or literature; n.i.—not identified. The compounds with only MS symbol in ID column were tentatively identified. The compounds for which pure standards were not available (without symbol S in the ID column) were quantified semi-quantitatively and their concentrations were expressed as equivalents of compounds with similar chemical structure assuming a response factor = 1. LRI_exp_—linear retention index obtained experimentally. Varieties: MI—Malvazija istarska, PO—Pošip, MA—Maraština, KR—Kraljevina, SK—Škrlet. Different superscript lowercase letters in a row represent statistically significant differences between mean values at *p* < 0.05 obtained by one-way ANOVA and least significant difference (LSD) test.

**Table 2:** Concentrations (μg/L relative to internal standard 2-octanol) of volatile aroma compounds found in Croatian monovarietal wines obtained by headspace solid-phase microextraction followed by comprehensive two-dimensional gas chromatography-mass spectrometry with time-of-flight mass spectrometric detection (HS-SPME/GC×GC-TOF-MS) sorted by compound class and descending Fisher *F*-ratio.

No.	Volatile Compounds	t_R_ (1D)	t_R_ (2D)	ID	LRI_exp_	LRI_lit_	*F*-Ratio	Variety				
		(min:s)	(min:s)					MI	PO	MA	KR	SK
	*Terpenes*											
1	α-Terpineol	18:47.2	00:01.2	MS, LRI	1710	1709	112.904	3.683 ± 1.391 ^b^	2.440 ± 0.424 ^c^	1.256 ± 0.455 ^d^	0.712 ± 0.369 ^d^	11.628 ± 0.495 ^a^
2	Limonene	08:01.0	00:01.8	S, MS, LRI	1191	1194	54.231	1.401 ± 0.746 ^b^	0.429 ± 0.261 ^c^	0.360 ± 0.105 ^c^	0.191 ± 0.114 ^c^	3.956 ± 0.291 ^a^
3	Linalool	15:50.0	00:01.1	S, MS, LRI	1541	1541	23.272	16.664 ± 7.072 ^b^	10.757 ± 1.712 ^c^	5.318 ± 1.567 ^d^	2.857 ± 0.970 ^d^	23.674 ± 3.494 ^a^
4	*trans*-Alloocimene	12:13.0	00:01.6	MS, LRI	1384	1388	22.080	0.362 ± 0.189 ^b^	0.137 ± 0.032 ^c^	0.088 ± 0.044 ^c^	0.040 ± 0.022 ^c^	0.753 ± 0.282 ^a^
5	*o*-Cymene	09:49.9	00:01.6	S, MS, LRI	1273	1268	20.745	1.305 ± 0.436 ^b^	0.631 ± 0.353 ^c^	0.441 ± 0.268 ^c^	0.278 ± 0.142 ^c^	2.613 ± 1.042 ^a^
6	γ-Dehydro-ar-himachalene	25:10.0	00:01.7	MS	2046	-	18.720	0.003 ± 0.003 ^b^	0.003 ± 0.003 ^b^	0.003 ± 0.002 ^b^	0.016 ± 0.006 ^a^	0.004 ± 0.004 ^b^
7	β-Myrcene	07:18.8	00:01.6	S, MS, LRI	1159	1159	17.244	2.351 ± 1.293 ^b^	0.885 ± 0.539 ^c^	0.331 ± 0.070 ^c^	0.183 ± 0.094 ^c^	4.353 ± 1.916 ^a^
8	Nerol	20:43.5	00:01.0	S, MS, LRI	1812	1811	16.944	0.568 ± 0.277 ^b^	0.220 ± 0.118 ^c^	0.225 ± 0.070 ^c^	0.077 ± 0.038 ^c^	0.810 ± 0.139 ^a^
9	*cis*-α-Ocimene	09:24.8	00:01.6	MS, LRI	1254	1255	14.724	2.278 ± 0.937 ^a^	0.754 ± 0.205 ^b^	0.459 ± 0.087 ^b^	0.337 ± 0.170 ^b^	3.070 ± 1.903 ^a^
10	*trans*-Linalool furan oxide	13:39.9	00:01.2	S, MS, LRI	1445	1450	14.601	0.444 ± 0.117 ^b^	0.480 ± 0.160 ^b^	0.221 ± 0.144 ^c^	0.157 ± 0.138 ^c^	0.872 ± 0.264 ^a^
11	Ho-trienol	17:07.0	00:01.0	MS, LRI	1607	1612	13.945	2.843 ± 1.206 ^a^	2.307 ± 0.568 ^a^	0.669 ± 0.405 ^b^	0.712 ± 0.395 ^b^	2.510 ± 0.219 ^a^
12	Geraniol	21:33.0	00:01.0	S, MS, LRI	1856	1857	11.761	0.432 ± 0.254 ^a^	0.225 ± 0.091 ^b^	0.124 ± 0.041 ^b^	0.084 ± 0.020 ^b^	0.589 ± 0.079 ^a^
13	Carvomenthenal	17:14.0	00:01.5	MS, LRI	1615	1629	11.321	0.381 ± 0.215 ^a^	0.193 ± 0.088 ^b^	0.142 ± 0.052 ^b^	0.057 ± 0.034 ^b^	0.525 ± 0.132 ^a^
14	Linalool ethyl ether	11:02.8	00:01.9	MS, LRI	1329	1331	10.900	5.419 ± 2.787 ^a^	1.744 ± 0.925 ^b^	0.998 ± 0.204 ^b^	0.444 ± 0.219 ^b^	6.365 ± 4.476 ^a^
15	4-Terpineol	17:00.0	00:01.3	S, MS, LRI	1600	1597	10.895	0.464 ± 0.097 ^b^	0.363 ± 0.069 ^cd^	0.418 ± 0.116 ^bc^	0.289 ± 0.083 ^d^	0.676 ± 0.045 ^a^
16	α-Terpinolene	10:03.7	00:01.9	S, MS, LRI	1284	1282	10.846	1.630 ± 0.872 ^b^	0.520 ± 0.288 ^c^	0.387 ± 0.217 ^c^	0.173 ± 0.085 ^c^	3.391 ± 2.459 ^a^
17	Geranyl ethyl ether	15:08.0	00:01.8	MS, LRI	1506	1506	10.391	2.042 ± 0.965 ^a^	0.710 ± 0.328 ^b^	0.500 ± 0.186 ^b^	0.222 ± 0.161 ^b^	2.960 ± 2.199 ^a^
18	Monoterpene (n.i.; *m/z* 93, 121, 136)	13:54.1	00:01.9	MS	1455	-	9.899	10.628 ± 4.686 ^a^	4.420 ± 1.974 ^b^	2.737 ± 1.576 ^b^	1.104 ± 0.702 ^b^	16.227 ± 12.511 ^a^
19	Neryl ethyl ether	14:26.5	00:01.8	MS, LRI	1477	1468	8.918	0.334 ± 0.175 ^b^	0.143 ± 0.065 ^bc^	0.100 ± 0.067 ^bc^	0.023 ± 0.021 ^c^	0.863 ± 0.729 ^a^
20	Monoterpene (n.i.; *m/z* 68, 93, 121)	08:15.2	00:01.8	MS	1202	-	8.901	0.252 ± 0.272 ^b^	0.126 ± 0.077 ^bc^	0.047± 0.021 ^c^	0.020 ± 0.015 ^c^	0.575 ± 0.180 ^a^
21	*p*-Cymenene	13:30.2	00:01.5	MS, LRI	1439	1438	7.409	1.332 ± 0.236 ^a^	0.899 ± 0.190 ^b^	0.918 ± 0.307 ^b^	0.733 ± 0.210 ^b^	1.711 ± 0.786 ^a^
22	*trans*-β-Ocimene	09:01.7	00:01.7	S, MS, LRI	1237	1241	6.833	0.078 ± 0.148 ^b^	0.067 ± 0.087 ^b^	0.099 ± 0.109 ^b^	0.014 ± 0.019 ^b^	0.919 ± 0.926 ^a^
23	β-Calacorene	22:43.0	00:01.8	MS, LRI	1919	1918	5.495	0.108 ± 0.018 ^ab^	0.075 ± 0.011 ^c^	0.078 ± 0.011 ^c^	0.131 ± 0.044 ^a^	0.072 ± 0.052 ^c^
24	*cis*-*Z*-α-Bisabolene epoxide	24:28.0	00:01.3	MS, LRI	2010	2007	5.329	0.002 ± 0.003 ^c^	0.008 ± 0.004 ^bc^	0.010 ± 0.009 ^b^	0.013 ± 0.006 ^ab^	0.023 ± 0.016 ^a^
25	*trans*-Linalool pyran oxide	19:34.0	00:01.1	MS, LRI	1751	1752	4.346	0.059 ± 0.018 ^ab^	0.082 ± 0.049 ^a^	0.029 ± 0.031 ^b^	0.031 ± 0.019 ^b^	0.086 ± 0.025 ^a^
26	Cadalene	27:46.2	00:01.6	MS, LRI	>2100	2191	3.956	0.047 ± 0.010 ^b^	0.039 ± 0.017 ^b^	0.035 ± 0.011 ^b^	0.064 ± 0.022 ^a^	0.032 ± 0.021 ^b^
27	Isogeraniol	20:55.4	00:01.0	MS, LRI	1822	1828	3.457	0.036 ± 0.030 ^a^	0.018 ± 0.014 ^ab^	0.005 ± 0.006 ^b^	0.010 ± 0.011 ^b^	0.015 ± 0.007 ^ab^
28	α-Terpinene	07:33.6	00:01.8	S, MS, LRI	1170	1175	3.225	0.033 ± 0.061 ^b^	0.006 ± 0.010 ^b^	0.006 ± 0.010 ^b^	0.007 ± 0.015 ^b^	0.106 ± 0.120 ^a^
29	Sesquiterpene (n.i.; *m/z* 119, 93, 69)	19:48.0	00:01.8	MS	1763	-	2.809	0.064 ± 0.010 ^a^	0.038 ± 0.020 ^b^	0.040 ± 0.014 ^b^	0.045 ± 0.012 ^b^	0.042 ± 0.038 ^b^
30	Menthol	17:42.0	00:01.1	MS, LRI	1644	1641	2.746	0.129 ± 0.050 ^b^	0.222 ± 0.283 ^b^	0.115 ± 0.032 ^b^	0.099 ± 0.023 ^b^	1.177 ± 1.847 ^a^
31	Citronellol	20:02.9	00:01.1	S, MS, LRI	1776	1777	2.724	0.303 ± 0.037 ^ab^	0.250 ± 0.089 ^ab^	0.260 ± 0.089 ^ab^	0.154 ± 0.074 ^b^	0.309 ± 0.229 ^ab^
32	α-Farnesene II	19:48.0	00:01.9	S, MS, LRI	1763	1762	2.118	0.053 ± 0.018	0.029 ± 0.016	0.026 ± 0.023	0.039 ± 0.021	0.047 ± 0.026
33	Monoterpene (n.i.; *m/z* 93, 121, 94)	20:48.1	00:01.7	MS	1816	-	2.054	0.056 ± 0.034	0.067 ± 0.027	0.041 ± 0.041	0.028 ± 0.015	0.028 ± 0.009
34	γ-Cadinene	19:55.0	00:02.1	MS, LRI	1769	1774	1.895	0.020 ± 0.003	0.013 ± 0.007	0.016 ± 0.004	0.014 ± 0.007	0.012 ± 0.011
35	(+)-Cuparene	21:05.0	00:02.0	MS, LRI	1831	1830	1.847	0.020 ± 0.001	0.014 ± 0.010	0.017 ± 0.004	0.012 ± 0.007	0.012 ± 0.010
36	*trans*-Calamenene	21:19.0	00:01.9	MS, LRI	1844	1837	1.780	0.063 ± 0.019	0.056 ± 0.020	0.049 ± 0.011	0.077 ± 0.028	0.056 ± 0.023
37	Citronellyl acetate	18:10.0	00:01.5	MS, LRI	1673	1668	1.652	0.056 ± 0.031	0.082 ± 0.059	0.041 ± 0.022	0.035 ± 0.017	0.063 ± 0.060
38	α-Calacorene	22:29.0	00:01.8	MS, LRI	1906	1916	1.533	0.020 ± 0.003	0.014 ± 0.008	0.015 ± 0.005	0.019 ± 0.009	0.012 ± 0.010
39	Dehydroaromadendrene	28:20.9	00:01.4	MS	>2100	-	1.306	0.001 ± 0.002	0.014 ± 0.036	0.001 ± 0.002	0.012 ± 0.015	0.028 ± 0.034
40	Terpene (n.i.; *m/z* 121, 93, 136)	15:08.0	00:01.8	MS	1506	-	1.276	0.891 ± 0.202	0.784 ± 0.328	0.788 ± 0.357	0.458 ± 0.172	0.836 ± 1.082
41	β-Cyclocitral	17:21.0	00:01.5	S, MS, LRI	1622	1629	1.195	0.067 ± 0.016	0.071 ± 0.017	0.066 ± 0.030	0.051 ± 0.014	0.052 ± 0.028
42	α-Farnesene I	18:17.0	00:01.9	S, MS, LRI	1681	1697	1.124	0.126 ± 0.043	0.074 ± 0.034	0.102 ± 0.058	0.115 ± 0.061	0.113 ± 0.046
43	2-Acetyl-2-carene	23:11.0	00:01.3	MS	1943	-	1.088	0.030 ± 0.048	0.037 ± 0.039	0.047 ± 0.050	0.010 ± 0.013	0.054 ± 0.013
44	γ-Isogeraniol	20:29.2	00:01.0	MS, LRI	1799	1800	1.071	0.148 ± 0.134	0.123 ± 0.083	0.087 ± 0.064	0.061 ± 0.046	0.087 ± 0.078
45	Cosmene	13:41.6	00:01.4	MS, LRI	1446	1460	0.990	0.109 ± 0.117	0.056 ± 0.056	0.106 ± 0.068	0.080 ± 0.054	0.027 ± 0.011
46	α-Curcumene	20:16.0	00:01.8	MS, LRI	1787	1782	0.736	0.026 ± 0.014	0.019 ± 0.007	0.021 ± 0.007	0.029 ± 0.017	0.022 ± 0.011
47	*trans*-Geranyl acetone	21:40.0	00:01.5	MS, LRI	1863	1868	0.734	0.409 ± 0.437	0.228 ± 0.071	0.206 ± 0.060	0.422 ± 0.568	0.152 ± 0.036
48	α-Bergamotene	16:18.0	00:02.4	MS, LRI	1565	1585	0.675	0.027 ± 0.011	0.037 ± 0.027	0.058 ± 0.071	0.041 ± 0.020	0.043 ± 0.024
49	Sesquiterpene (n.i.; *m/z* 93, 80, 121)	20:16.0	00:02.0	MS	1787	-	0.596	0.021 ± 0.007	0.014 ± 0.008	0.020 ± 0.013	0.022 ± 0.014	0.022 ± 0.021
50	Neryl acetate	19:20.7	00:01.5	MS, LRI	1739	1742	0.576	0.085 ± 0.021	0.060 ± 0.050	0.058 ± 0.038	0.078 ± 0.054	0.092 ± 0.083
51	4-Thujanol	15:20.1	00:01.7	MS	1516	-	0.444	0.055 ± 0.040	0.051 ± 0.029	0.067 ± 0.043	0.068 ± 0.024	0.046 ± 0.012
52	Nerolidol	24:56.2	00:01.3	S, MS, LRI	2034	2034	0.434	0.114 ± 0.049	0.102 ± 0.040	0.124 ± 0.087	0.142 ± 0.058	0.131 ± 0.064
53	Geranyl acetate	19:55.0	00:01.5	S, MS, LRI	1769	1768	0.413	0.037 ± 0.015	0.048 ± 0.038	0.035 ± 0.018	0.034 ± 0.013	0.034 ± 0.033
	*C_13_-norisoprenoids*											
54	1,2-Dihydro-1,4,6-trimethylnaphthalene	25:38.3	00:01.5	MS	2071	-	7.148	0.001 ± 0.001 ^b^	0.000 ± 0.000 ^b^	0.009 ± 0.008 ^b^	0.023 ± 0.018 ^a^	0.002 ± 0.001 ^b^
55	β-Damascenone	21:05.0	00:01.5	S, MS, LRI	1831	1832	6.736	8.245 ± 2.169 ^a^	5.770 ± 2.963 ^bc^	4.338 ± 1.563 ^cd^	6.981 ± 1.067 ^ab^	2.336 ± 0.303 ^d^
56	α-Ionene	14:28.9	00:02.0	MS	1479	-	5.379	0.045 ± 0.017 ^bc^	0.017 ± 0.023 ^c^	0.069 ± 0.047 ^ab^	0.085 ± 0.031 ^a^	0.032 ± 0.017 ^bc^
57	Norisoprenoid (n.i.; *m/z* 69, 121, 105)	20:00.5	00:01.6	MS	1774	-	5.061	0.248 ± 0.076 ^a^	0.163 ± 0.101 ^b^	0.127 ± 0.060 ^bc^	0.202 ± 0.058 ^ab^	0.055 ± 0.025 ^c^
58	3,4-Dehydro-β-ionone	18:38.0	00:01.8	MS	1702	-	4.920	0.032 ± 0.009 ^a^	0.011 ± 0.010 ^b^	0.036 ± 0.017 ^a^	0.040 ± 0.020 ^a^	0.020 ± 0.004 ^ab^
59	Vitispirane I	15:29.0	00:01.9	MS, LRI	1524	1524	3.470	1.155 ± 0.481 ^c^	3.501 ± 1.555 ^ab^	4.058 ± 2.588 ^a^	2.200 ± 0.966 ^bc^	3.007 ± 2.552 ^abc^
60	1,2-Dihydro-1,5,8-trimethylnaphthalene	19:41.0	00:01.6	MS	1757	-	2.579	0.360 ± 0.109	0.363 ± 0.186	0.682 ± 0.399	0.770 ± 0.451	1.085 ± 1.062
61	Actinidol ethyl ether II	18:52.0	00:01.9	MS, LRI	1714	1723	2.179	0.099 ± 0.082	0.181 ± 0.106	0.167 ± 0.130	0.055 ± 0.030	0.133 ± 0.067
62	1,2-Dihydro-1,1,6-trimethylnaphthalene (TDN)	19:06.0	00:01.6	S, MS, LRI	1727	1729	0.885	0.021 ± 0.012	0.019 ± 0.010	0.031 ± 0.021	0.026 ± 0.018	0.015 ± 0.015
63	*trans*-1-(2,3,6-Trimethylphenyl)buta-1,3-diene (TPB)	21:12.0	00:01.5	MS, LRI	1837	1832	0.433	0.065 ± 0.029	0.057 ± 0.036	0.076 ± 0.048	0.057 ± 0.031	0.050 ± 0.025
	*Benzenoids*											
64	Ethyl benzoate	18:17.0	00:01.2	MS, LRI	1681	1678	20.194	0.759 ± 0.232 ^b^	1.493 ± 0.366 ^a^	0.653 ± 0.128 ^b^	0.533 ± 0.118 ^b^	0.570 ± 0.115 ^b^
65	1,1’-Oxybisbenzene	24:20.1	00:01.3	MS, LRI	2003	2017	18.956	0.011 ± 0.002 ^a^	0.004 ± 0.002 ^b^	0.004 ± 0.003 ^b^	0.003 ± 0.001 ^b^	0.003 ± 0.001 ^b^
66	2,3-Dihydro-1,1,5,6-tetramethyl-1H-indene I	18:17.0	00:01.7	MS	1681	-	9.842	0.076 ± 0.019 ^bc^	0.027 ± 0.030 ^d^	0.105 ± 0.039 ^ab^	0.139 ± 0.054 ^a^	0.050 ± 0.013 ^cd^
67	Octylbenzene	19:27.0	00:01.8	MS, LRI	1745	1746	8.638	0.109 ± 0.011 ^a^	0.065 ± 0.022 ^b^	0.074 ± 0.010 ^b^	0.072 ± 0.012 ^b^	0.070 ± 0.032 ^b^
68	*trans*-Edulan	17:07.0	00:01.8	MS, LRI	1607	1602	7.938	0.039 ± 0.016 ^b^	0.084 ± 0.018 ^a^	0.056 ± 0.019 ^b^	0.042 ± 0.010 ^b^	0.035 ± 0.031 ^b^
69	2,3-Dihydro-1,1,5,6-tetramethyl-1H-indene II	17:28.0	00:01.7	MS	1629	-	7.671	0.023 ± 0.006 ^bc^	0.007 ± 0.010 ^c^	0.038 ± 0.020 ^ab^	0.056 ± 0.030 ^a^	0.016 ± 0.004 ^bc^
70	3-Methylphenylacetylene	14:33.0	00:01.3	MS, LRI	1481	1450.9	7.438	0.012 ± 0.002 ^b^	0.016 ± 0.006 ^b^	0.013 ± 0.007 ^b^	0.032 ± 0.013 ^a^	0.028 ± 0.012 ^a^
71	Benzoic acid	30:48.9	00:00.8	S, MS, LRI	>2100	2438	6.952	0.269 ± 0.021 ^bc^	0.412 ± 0.064 ^a^	0.350 ± 0.047 ^a^	0.233 ± 0.107 ^c^	0.334 ± 0.111 ^ab^
72	Azulene	19:34.0	00:01.3	MS, LRI	1751	1746	6.891	0.240 ± 0.058 ^a^	0.181 ± 0.044 ^b^	0.165 ± 0.042 ^bc^	0.130 ± 0.039 ^c^	0.117 ± 0.039 ^c^
73	Trimethyl-tetrahydronaphthalene	13:12.1	00:01.8	MS	1426	-	6.830	0.007 ± 0.012 ^c^	0.002 ± 0.004 ^c^	0.056 ± 0.056 ^ab^	0.102 ± 0.070 ^a^	0.007 ± 0.008 ^c^
74	Benzeneacetaldehyde	17:49.0	00:01.1	S, MS, LRI	1651	1648	6.827	5.920 ± 1.513 ^c^	10.269 ± 2.174 ^a^	8.487 ± 2.065 ^ab^	5.884 ± 2.053 ^c^	6.501 ± 1.630 ^bc^
75	*m*-Methoxyanisole	19:48.0	00:01.2	MS, LRI	1763	1761	6.715	0.009 ± 0.013 ^c^	0.035 ± 0.021 ^b^	0.009 ± 0.011 ^c^	0.013 ± 0.013 ^bc^	0.070 ± 0.052 ^a^
76	Benzenoid (n.i.; *m/z* 115, 130, 129)	16:37.2	00:01.4	MS	1581	-	5.764	0.007 ± 0.003 ^b^	0.005 ± 0.004 ^b^	0.005 ± 0.004 ^b^	0.014 ± 0.005 ^a^	0.009 ± 0.003 ^ab^
77	6-[1-(Hydroxymethyl)vinyl]-4,8a-dimethyl-1,2,4a,5,6,7,8,8a-octahydro-2-naphthalenol	19:54.7	00:02.0	MS	1769	-	5.587	0.010 ± 0.014 ^b^	0.010 ± 0.011 ^b^	0.009 ± 0.010 ^b^	0.041 ± 0.023 ^a^	0.013 ± 0.011 ^b^
78	Prehnitene	14:40.0	00:01.5	MS, LRI	1486	1476	5.516	0.198 ± 0.035 ^ab^	0.160 ± 0.052 ^bc^	0.249 ± 0.080 ^a^	0.124 ± 0.034 ^c^	0.124 ± 0.089 ^c^
79	2-Methylnaphthalene	22:15.0	00:01.3	MS, LRI	1894	1872	4.321	0.021 ± 0.004 ^a^	0.015 ± 0.003 ^b^	0.014 ± 0.004 ^b^	0.015 ± 0.005 ^b^	0.012 ± 0.003 ^b^
80	*meso*-2,3-Diphenylbutane	17:07.0	00:01.3	MS	1607	-	4.291	0.025 ± 0.017 ^b^	0.050 ± 0.016 ^a^	0.037 ± 0.009 ^ab^	0.027 ± 0.010 ^b^	0.032 ± 0.007 ^ab^
81	4-Ethylbenzaldehyde	19:34.0	00:01.2	MS, LRI	1751	1747	4.168	0.059 ± 0.010 ^ab^	0.067 ± 0.014 ^a^	0.060 ± 0.015 ^ab^	0.043 ± 0.010 ^c^	0.048 ± 0.008 ^bc^
82	Styrene	09:27.4	00:05.0	MS, LRI	1256	1257	3.578	2.067 ± 0.516 ^ab^	2.462 ± 0.859 ^a^	2.161 ± 0.275 ^ab^	1.701 ± 0.419 ^bc^	1.223 ± 0.083 ^c^
83	Ethyl *o*-methylbenzoate	19:34.2	00:01.3	MS	1751	-	3.148	0.039 ± 0.005 ^ab^	0.041 ± 0.005 ^a^	0.034 ± 0.005 ^abc^	0.028 ± 0.013 ^c^	0.027 ± 0.015 ^c^
84	2,3-Dihydrobenzofuran	16:46.0	00:01.2	MS	1588	-	3.122	0.025 ± 0.011 ^b^	0.046 ± 0.014 ^a^	0.039 ± 0.013 ^ab^	0.029 ± 0.014 ^b^	0.029 ± 0.001 ^b^
85	Benzofuran	15:01.0	00:01.1	MS, LRI	1500	1496	3.121	0.040 ± 0.013 ^b^	0.061 ± 0.022 ^a^	0.047 ± 0.015 ^ab^	0.040 ± 0.013 ^b^	0.030 ± 0.005 ^b^
86	Benzonitrile	17:00.0	00:01.0	MS, LRI	1600	1591	3.041	0.033 ± 0.015 ^b^	0.064 ± 0.026 ^a^	0.052 ± 0.021 ^ab^	0.038 ± 0.016 ^b^	0.039 ± 0.005 ^ab^
87	α,α-Dimethylbenzenemethanol	19:55.2	00:01.0	MS, LRI	1769	1770	2.981	0.021 ± 0.009 ^b^	0.033 ± 0.018 ^b^	0.024 ± 0.008 ^b^	0.027 ± 0.007 ^b^	0.203 ± 0.309 ^a^
88	Methyl 2-(benzyloxy)propanoate	23:18.0	00:01.3	MS	1949	-	2.958	0.825 ± 0.861 ^a^	0.144 ± 0.151 ^b^	0.107 ± 0.138 ^b^	0.211 ± 0.456 ^b^	0.018 ± 0.017 ^b^
89	α-Methylstyrene	11:10.8	00:01.3	MS, LRI	1336	1325	2.727	0.006 ± 0.007	0.014 ± 0.006	0.015 ± 0.007	0.017 ± 0.008	0.118 ± 0.192
90	3-Ethylbenzaldehyde	18:59.4	00:01.2	MS, LRI	1721	1732	2.631	0.088 ± 0.013	0.092 ± 0.016	0.086 ± 0.023	0.065 ± 0.021	0.075 ± 0.004
91	3-Methylbenzofuran	21:19.0	00:01.1	MS	1844	-	2.594	0.027 ± 0.006	0.046 ± 0.012	0.034 ± 0.016	0.032 ± 0.014	0.034 ± 0.006
92	Styralyl isobutyrate	23:31.8	00:01.4	MS	1961	-	2.462	0.065 ± 0.022	0.191 ± 0.121	0.166 ± 0.115	0.149 ± 0.069	0.097 ± 0.023
93	2’,5’-Dimethylcrotonophenone	24:00.0	00:01.3	MS	1985	-	2.362	0.045 ± 0.017	0.040 ± 0.029	0.026 ± 0.019	0.040 ± 0.011	0.010 ± 0.009
94	Ethyl benzenepropanoate	22:15.0	00:01.3	MS, LRI	1894	1892	2.328	0.404 ± 0.256	0.509 ± 0.353	0.314 ± 0.180	0.206 ± 0.052	0.131 ± 0.079
95	1-Methylnapthalene	21:40.0	00:01.3	MS, LRI	1863	1878	2.319	0.017 ± 0.005	0.016 ± 0.007	0.014 ± 0.003	0.011 ± 0.003	0.010 ± 0.001
96	Ethyl salicylate	21:35.8	00:01.1	S, MS, LRI	1859	1837	2.288	0.037 ± 0.054	0.005 ± 0.008	0.027 ± 0.049	0.011 ± 0.012	0.177 ± 0.296
97	Methyl salicylate	20:16.0	00:01.2	S, MS, LRI	1787	1789	2.151	3.457 ± 1.576	4.548 ± 6.357	2.447 ± 1.435	1.979 ± 0.985	14.629 ± 21.656
98	2-Methylbenzaldehyde	17:27.8	00:01.1	MS, LRI	1629	1622	2.145	0.041 ± 0.014	0.057 ± 0.023	0.034 ± 0.023	0.032 ± 0.012	0.051 ± 0.025
99	Durene	13:16.0	00:01.5	MS, LRI	1429	1435	2.038	0.085 ± 0.033	0.084 ± 0.040	0.080 ± 0.027	0.056 ± 0.034	0.034 ± 0.028
100	Butylated hydroxytoluene	22:43.0	00:01.5	MS, LRI	1919	1920	1.558	0.314 ± 0.073	0.351 ± 0.163	0.295 ± 0.107	0.228 ± 0.067	0.210 ± 0.146
101	Ethyl benzeneacetate	20:30.0	00:01.2	MS, LRI	1799	1788	1.474	1.334 ± 0.310	3.173 ± 0.863	2.503 ± 1.245	3.294 ± 3.186	3.112 ± 2.566
102	*p*-Methoxyanisole	19:34.2	00:01.2	MS, LRI	1751	1752	1.309	0.153 ± 0.041	0.184 ± 0.074	0.141 ± 0.044	0.197 ± 0.052	0.191 ± 0.075
103	Benzeneacetic acid	32:45.2	00:00.8	MS, LRI	>2100	2519	1.243	0.002 ± 0.000	0.005 ± 0.005	0.008 ± 0.007	0.004 ± 0.007	0.009 ± 0.013
104	Benzyl alcohol	22:01.0	00:00.9	S, MS, LRI	1881	1877	1.243	0.914 ± 1.413	2.007 ± 2.953	0.468 ± 0.243	0.354 ± 0.079	0.565 ± 0.297
105	2-Hydroxybenzeneacetic acid	23:11.0	00:01.0	MS	1943	-	1.238	0.003 ± 0.008	0.008 ± 0.004	0.003 ± 0.003	0.005 ± 0.001	0.005 ± 0.002
106	2-Ethyl-*m*-xylene	11:58.5	00:01.6	MS, LRI	1373	1372	1.224	0.106 ± 0.064	0.112 ± 0.059	0.082 ± 0.043	0.094 ± 0.040	0.040 ± 0.035
107	Benzaldehyde	15:16.8	00:05.8	S, MS, LRI	1514	1509	1.094	1.935 ± 0.978	4.662 ± 6.475	1.479 ± 0.377	2.161 ± 0.535	2.934 ± 2.144
108	2-(1,1-Dimethylethyl)-1,4-dimethoxybenzene	22:29.0	00:01.4	MS, LRI	1906	1870	0.996	0.069 ± 0.010	0.061 ± 0.014	0.066 ± 0.017	0.075 ± 0.021	0.051 ± 0.044
109	*trans*-1,2-Diphenylcyclobutane	20:58.0	00:01.3	MS	1825	-	0.674	0.003 ± 0.002	0.003 ± 0.002	0.004 ± 0.007	0.002 ± 0.003	0.000 ± 0.000
110	3,3-Dimethoxy-1-phenylpropan-1-one	09:56.8	00:05.6	MS	1278	-	0.569	0.037 ± 0.032	0.048 ± 0.054	0.052 ± 0.059	0.025 ± 0.027	0.062 ± 0.031
111	*trans*-Anethole	21:12.0	00:01.3	S, MS, LRI	1837	1834	0.502	0.423 ± 0.223	0.449 ± 0.199	0.390 ± 0.213	0.395 ± 0.309	0.613 ± 0.344
112	*cis*-Anethole	19:55.0	00:01.3	S, MS, LRI	1769	1780	0.322	0.013 ± 0.005	0.015 ± 0.007	0.014 ± 0.004	0.014 ± 0.007	0.017 ± 0.010
113	1,2-Dimethylbenzene	07:41.0	00:05.1	MS, LRI	1176	1175	0.176	0.468 ± 0.333	0.412 ± 0.402	0.503 ± 0.355	0.356 ± 0.312	0.432 ± 0.375
	*Hydrocarbons*											
114	Pentadecane	14:54.0	00:02.7	S, MS, LRI	1496	1500	1.699	0.251 ± 0.055	0.195 ± 0.066	0.222 ± 0.104	0.205 ± 0.034	0.140 ± 0.045
115	2,3,3-Trimethyl-*cis*-4-nonene	10:42.0	00:01.6	MS	1313	-	1.585	0.078 ± 0.095	0.052 ± 0.034	0.031 ± 0.008	0.016 ± 0.009	0.026 ± 0.031
116	Hexadecane	17:00.0	00:02.7	S, MS, LRI	1600	1600	1.332	0.170 ± 0.025	0.140 ± 0.051	0.162 ± 0.082	0.134 ± 0.038	0.100 ± 0.031
117	*cis*,*trans*-1,3,5-Octatriene	08:08.4	00:00.7	MS	1196	-	1.087	0.376 ± 0.146	0.364 ± 0.125	0.315 ± 0.140	0.245 ± 0.137	0.329 ± 0.029
118	2,6,8-Trimethyl-*trans*-4-nonene	11:13.5	00:02.5	MS	1338	-	0.464	0.048 ± 0.083	0.029 ± 0.052	0.019 ± 0.039	0.032 ± 0.043	0.002 ± 0.002
	*Aldehydes*											
119	Decanal	14:54.0	00:01.5	S, MS, LRI	1496	1497	3.149	0.068 ± 0.041 ^a^	0.068 ± 0.035 ^a^	0.051 ± 0.040 ^ab^	0.014 ±0.011 ^b^	0.060 ± 0.008 ^ab^
120	*trans*-2-Decenal	17:49.0	00:01.4	MS, LRI	1651	1647	2.553	0.116 ± 0.030	0.131 ± 0.043	0.109 ± 0.030	0.071 ± 0.034	0.094 ± 0.065
121	*trans*-2-Octenal	13:25.3	00:01.3	S, MS, LRI	1435	1432	2.307	0.249 ± 0.301	0.017 ± 0.023	0.120 ± 0.038	0.105 ± 0.092	0.021 ± 0.020
122	Undecanal	17:07.0	00:01.5	S, MS, LRI	1607	1606	1.967	0.061 ± 0.020	0.053 ± 0.016	0.040 ± 0.010	0.041 ± 0.010	0.051 ± 0.031
123	Dodecanal	19:06.0	00:01.6	MS, LRI	1727	1722	1.471	0.066 ± 0.015	0.062 ± 0.019	0.050 ± 0.014	0.056 ± 0.022	0.043 ± 0.011
124	3,3-Dimethyl-2-oxobutanal	11:24.8	00:01.9	MS	1347	-	0.279	0.108 ± 0.091	0.158 ± 0.178	0.106 ± 0.104	0.226 ± 0.522	0.078 ± 0.082
125	Nonanal	12:40.6	00:01.5	S, MS, LRI	1405	1404	0.206	10.699 ± 7.689	12.183 ± 7.410	11.470 ± 8.481	8.722 ± 6.668	10.800 ± 6.868
	*Ketones*											
126	1,4,7,10,13-Pentaoxacyclononadecane-14,19-dione	27:35.0	00:01.3	MS	>2100	-	9.721	0.027 ± 0.038 ^b^	0.013 ± 0.014 ^b^	0.005 ± 0.003 ^b^	0.022 ± 0.020 ^b^	0.107 ± 0.038 ^a^
127	α-Isophorone	16:46.0	00:01.3	S, MS, LRI	1588	1593	7.380	0.116 ± 0.022 ^a^	0.101 ± 0.032 ^a^	0.047 ± 0.024 ^b^	0.093 ± 0.021 ^a^	0.093 ± 0.029 ^a^
128	Cyclohexylideneacetone	17:35.0	00:01.8	MS	1637	-	6.967	0.097 ± 0.097 ^c^	0.671 ± 0.428 ^b^	0.424 ± 0.259 ^bc^	0.546 ± 0.254 ^b^	1.194 ± 0.691 ^a^
129	Acetophenone	17:56.0	00:01.1	S, MS, LRI	1659	1660	6.036	0.297 ± 0.048 ^cd^	0.557 ± 0.116 ^a^	0.415 ± 0.107 ^bc^	0.271 ± 0.078 ^d^	0.487 ± 0.348 ^ab^
130	2-Undecanone	16:53.4	00:01.5	MS, LRI	1594	1598	4.027	0.755 ± 0.471 ^a^	0.320 ± 0.097 ^b^	0.294 ± 0.104 ^b^	0.347 ± 0.246 ^b^	0.215 ± 0.162 ^b^
131	4,4-(Ethylenedioxy)-2-pentanone	19:20.0	00:01.1	MS	1739	-	3.787	0.166 ± 0.053 ^ab^	0.245 ± 0.111 ^a^	0.145 ± 0.089 ^b^	0.102 ± 0.020 ^b^	0.102 ± 0.067 ^b^
132	Unsaturated diketone (n.i.; *m/z* 43, 99, 71)	14:26.0	00:01.1	MS	1477	-	3.213	0.137 ± 0.151 ^b^	0.361 ± 0.123 ^a^	0.269 ± 0.095 ^ab^	0.170 ± 0.174 ^b^	0.150 ± 0.114 ^b^
133	3-Undecanone	16:20.3	00:01.6	MS, LRI	1567	1571	3.176	0.455 ± 0.440 ^a^	0.075 ± 0.051 ^b^	0.081 ± 0.040 ^b^	0.123 ± 0.265 ^b^	0.025 ± 0.021 ^b^
134	3-Tridecanone	20:16.0	00:01.7	MS, LRI	1787	1755	3.120	0.036 ± 0.023 ^a^	0.010 ± 0.008 ^b^	0.008 ± 0.009 ^b^	0.018 ± 0.026 ^ab^	0.006 ± 0.005 ^b^
135	1b,5,5,6a-Tetramethyl-octahydro-1-oxa-cyclopropa[a]inden-6-one	18:31.0	00:02.1	MS	1695	-	2.570	0.016 ± 0.019	0.042 ± 0.038	0.047 ± 0.054	0.088 ± 0.068	0.123 ± 0.138
136	3-(Acetoxy)-4-methyl-2-pentanone	14:07.3	00:01.3	MS	1464	-	2.528	0.012 ± 0.021	0.055 ± 0.055	0.073 ± 0.057	0.025 ± 0.025	0.031 ± 0.027
137	*trans*-5-Methyl-2-(1-methylethyl)-cyclohexanone	14:08.1	00:01.6	MS, LRI	1464	1473	2.282	0.041 ± 0.045	0.312 ± 0.657	0.077 ± 0.050	0.058 ± 0.030	1.167 ± 1.900
138	4-(1,1-Dimethylethyl)-cyclohexanone	17:35.0	00:01.5	MS, LRI	1637	1645	2.032	0.057 ± 0.095	0.124 ± 0.122	0.025 ± 0.033	0.011 ± 0.011	0.092 ± 0.125
139	1-Phenyl-1-propanone	19:20.2	00:01.2	MS, LRI	1739	1744	1.745	0.019 ± 0.018	0.030 ± 0.013	0.015 ± 0.007	0.015 ± 0.004	0.021 ± 0.005
140	2-Nonanone	12:34.0	00:01.4	S, MS, LRI	1401	1402	1.689	10.162 ± 9.346	4.449 ± 3.737	6.475 ± 7.123	3.195 ± 1.103	2.225 ± 2.037
141	2H-Pyran-2,6(3H)-dione	24:10.5	00:00.8	MS	1995	-	1.599	0.485 ± 0.190	0.661 ± 0.326	0.564 ± 0.226	0.397 ± 0.138	0.394 ± 0.069
142	2-Heptanone	07:46.5	00:05.2	S, MS, LRI	1180	1180	1.445	0.692 ± 0.406	0.605 ± 0.416	1.117 ± 1.197	0.301 ± 0.265	0.502 ± 0.401
143	2,2-Dimethyl-1,3-dioxane-4,6-dione	18:17.0	00:01.1	MS	1681	-	1.167	0.032 ± 0.014	0.022 ± 0.021	0.037 ± 0.010	0.035 ± 0.006	0.026 ± 0.023
144	2-Decanone	14:47.0	00:01.5	MS, LRI	1491	1491	0.990	0.502 ± 0.298	0.457 ± 0.128	0.377 ± 0.175	0.432 ± 0.159	0.255 ± 0.107
145	Acetoin	10:07.9	00:00.8	S, MS, LRI	1287	1287	0.732	0.054 ± 0.017	0.071 ± 0.048	0.061 ± 0.026	0.093 ± 0.083	0.058 ± 0.024
146	2,6-Di(tert-butyl)-4-hydroxy-4-methyl-2,5-cyclohexadien-1-one	25:45.0	00:01.1	MS, LRI	2077	2094	0.694	0.009 ± 0.003	0.010 ± 0.006	0.010 ± 0.007	0.014 ± 0.011	0.013 ± 0.008
147	2-Cyclohexene-1,4-dione	19:33.7	00:01.0	MS	1751	-	0.669	0.006 ± 0.010	0.027 ± 0.031	0.093 ± 0.214	0.031 ± 0.060	0.055 ± 0.078
148	3,4-Dihydroxy-cyclobutene-1,2-dione	18:10.0	00:01.1	MS	1673	-	0.409	0.089 ± 0.031	0.075 ± 0.037	0.060 ± 0.053	0.078 ± 0.059	0.073 ± 0.022
149	5-Methyl-5-hepten-2-one	11:24.0	00:01.3	MS, LRI	1346	1343	0.215	0.147 ± 0.160	0.113 ± 0.085	0.125 ± 0.023	0.116 ± 0.112	0.170 ± 0.130
	*Alcohols*											
150	4-Methyl-1-heptanol	12:42.1	00:01.6	MS, LRI	1406	1409	23.056	0.313 ± 0.161 ^b^	0.115 ± 0.060 ^c^	0.056 ± 0.047 ^c^	0.033 ± 0.031 ^c^	0.638 ± 0.209 ^a^
151	*cis*-3-Hexen-1-ol	12:20.0	00:00.9	S, MS, LRI	1390	1386	15.611	7.191 ± 2.621 ^c^	15.988 ± 3.409 ^a^	11.216 ± 4.880 ^b^	3.383 ± 0.607 ^d^	5.727 ± 3.198 ^cd^
152	2-Heptanol	10:56.0	00:01.0	S, MS, LRI	1324	1320	7.290	0.943 ± 0.327 ^bc^	1.984 ± 0.923 ^a^	1.044 ± 0.321 ^bc^	0.601 ± 0.251 ^c^	1.571 ± 0.480 ^ab^
153	2-Penten-1-ol	10:56.2	00:00.8	MS, LRI	1324	1321	5.588	0.044 ± 0.021 ^a^	0.045 ± 0.014 ^a^	0.044 ± 0.017 ^a^	0.012 ± 0.002 ^b^	0.040 ± 0.012 ^a^
154	3-Octanol	12:36.4	00:01.1	MS, LRI	1402	1406	5.108	0.082 ± 0.068 ^b^	0.185 ± 0.070 ^a^	0.062 ± 0.053 ^b^	0.072 ± 0.050 ^b^	0.054 ± 0.054 ^b^
155	1-Undecanol	21:49.4	00:01.1	MS, LRI	1871	1883	5.052	0.004 ± 0.006 ^b^	0.023 ± 0.015 ^a^	0.007 ± 0.012 ^b^	0.022 ± 0.010 ^a^	0.022 ± 0.007 ^a^
156	Alcohol (n.i.; *m/z* 69, 41, 84)	15:02.3	00:01.0	MS	1501	-	4.278	0.491 ± 0.778 ^bc^	0.848 ± 0.393 ^ab^	1.178 ± 0.637 ^a^	0.192 ± 0.081 ^c^	0.079 ± 0.036 ^c^
157	1-Octen-3-ol	13:51.0	00:01.0	S, MS, LRI	1453	1452	3.832	3.494 ± 2.869 ^b^	5.797 ± 1.668 ^a^	2.467 ± 0.654 ^b^	2.597 ± 1.254 ^b^	3.015 ± 1.251 ^b^
158	2-Decanol	17:20.3	00:01.1	MS, LRI	1621	1621	3.058	0.023 ± 0.011 ^b^	0.047 ± 0.025 ^b^	0.021 ± 0.015 ^b^	0.024 ± 0.013 ^b^	0.212 ± 0.318 ^a^
159	2,3-Butanediol II	16:25.2	00:00.8	S, MS, LRI	1571	1567	2.708	1.964 ± 0.450	2.601 ± 0.580	2.586 ± 1.276	1.421 ± 0.780	2.007 ± 0.180
160	4-Hepten-1-ol	14:54.5	00:00.9	MS, LRI	1496	1502	2.635	0.105 ± 0.065	0.093 ± 0.090	0.125 ± 0.060	0.044 ± 0.025	0.172 ± 0.057
161	6-Methyl-5-hepten-2-ol	14:05.8	00:01.0	S, MS, LRI	1463	1466	2.465	0.034 ± 0.006	0.050 ± 0.015	0.042 ± 0.013	0.037 ± 0.011	0.030 ± 0.012
162	3-Methyl-1-pentanol	11:03.7	00:00.9	S, MS, LRI	1330	1332	2.387	5.790 ± 1.421	5.799 ± 1.504	6.404 ± 2.937	3.575 ± 1.820	4.453 ± 0.203
163	2,3-Butanediol I	15:36.0	00:02.1	S, MS, LRI	1530	1542	2.291	3.399 ± 1.779	4.339 ± 1.812	3.931 ± 2.279	1.744 ± 1.027	3.597 ± 0.875
164	Alcohol (n.i.; *m/z* 45, 55, 43)	14:17.6	00:01.0	MS	1471	-	2.157	0.137 ± 0.120	0.012 ± 0.022	0.096 ± 0.114	0.093 ± 0.042	0.054 ± 0.043
165	1-Decanol	20:02.0	00:01.1	S, MS, LRI	1775	1778	2.086	0.710 ± 0.219	0.625 ± 0.100	0.597 ± 0.138	0.812 ± 0.097	0.642 ± 0.209
166	3,5-Dimethyl-4-heptanol	19:35.2	00:00.8	MS	1752	-	2.033	0.043 ± 0.031	0.104 ± 0.063	0.098 ± 0.058	0.053 ± 0.056	0.068 ± 0.002
167	2-Ethylhexanol	14:40.0	00:01.0	MS, LRI	1486	1484	1.756	4.569 ± 0.735	4.664 ± 0.537	4.826 ± 0.481	4.169 ± 0.465	4.122 ± 0.201
168	8-Methyl-1,8-nonanediol	10:56.2	00:01.1	MS	1324	-	1.454	0.123 ± 0.079	0.199 ± 0.084	0.223 ± 0.122	0.206 ± 0.084	0.133 ± 0.116
169	3,4-Nonadienol	19:48.0	00:01.0	MS, LRI	1763	1754	1.445	0.017 ± 0.025	0.016 ± 0.013	0.007 ± 0.005	0.003 ± 0.002	0.005 ± 0.001
170	1-Pentanol	08:53.5	00:00.5	S, MS, LRI	1231	1242	1.272	0.082 ± 0.091	0.135 ± 0.100	0.112 ± 0.076	0.058 ± 0.060	0.039 ± 0.014
171	3-Ethyl-4-octanol	18:24.9	00:01.3	MS	1689	-	1.225	0.364 ± 0.208	0.304 ± 0.189	0.479 ± 0.198	0.471 ± 0.108	0.331 ± 0.229
172	2-Octen-1-ol	17:14.0	00:01.0	S, MS, LRI	1615	1622	1.115	0.151 ± 0.206	0.114 ± 0.060	0.077 ± 0.033	0.041 ± 0.015	0.057 ± 0.020
173	2-Undecanol	19:13.0	00:01.2	MS, LRI	1733	1738	0.841	0.173 ± 0.218	0.330 ± 0.184	0.226 ± 0.137	0.241 ± 0.094	0.278 ± 0.205
174	4-Methyl-1-pentanol	10:49.0	00:00.9	MS, LRI	1319	1319	0.819	2.297 ± 1.083	2.626 ± 0.800	3.318 ± 2.010	2.130 ± 2.101	1.782 ± 0.130
175	4-Ethyl-3-octanol	15:08.0	00:01.2	MS	1506	-	0.721	0.400 ± 0.059	0.374 ± 0.067	0.430 ± 0.232	0.389 ± 0.202	0.245 ± 0.213
176	2-Nonanol	15:15.0	00:01.1	S, MS, LRI	1512	1518	0.715	0.512 ± 0.187	0.581 ± 0.344	0.429 ± 0.127	0.398 ± 0.169	0.468 ± 0.272
177	1-Nonanol	18:03.3	00:01.0	S, MS, LRI	1666	1661	0.580	1.420 ± 1.635	0.776 ± 0.538	2.515 ± 3.679	1.557 ± 2.268	1.018 ± 1.218
178	1-Heptanol	13:58.2	00:00.9	S, MS, LRI	1458	1457	0.560	1.238 ± 0.721	1.394 ± 0.657	1.120 ± 0.904	0.875 ± 0.381	1.183 ± 0.341
179	2-Methyl-1-pentanol	10:28.5	00:00.9	S, MS, LRI	1303	1297	0.229	0.262 ± 0.326	0.200 ± 0.248	0.323 ± 0.434	0.312 ± 0.145	0.199 ± 0.124
180	*trans*-4-*tert*-Butylcyclohexanol	19:43.3	00:01.1	MS, LRI	1759	1730	0.189	0.136 ± 0.326	0.149 ± 0.283	0.267 ± 0.447	0.204 ± 0.181	0.216 ± 0.332
181	2-Octanol (internal standard)	13:09.0	00:01.0	S, MS, LRI	1424	1418		40.000 ± 0.000	40.000 ± 0.000	40.000 ± 0.000	40.000 ± 0.000	40.000 ± 0.000
	*Acids*											
182	Propionic acid	15:43.0	00:00.7	S, MS, LRI	1536	1540	4.365	1.294 ± 0.324 ^ab^	1.631 ± 0.472 ^a^	1.159 ± 0.294 ^b^	0.946 ± 0.158 ^b^	0.991 ± 0.348 ^b^
183	Acid (n.i.; *m/z* 74, 45, 73)	14:33.0	00:01.1	MS, LRI	1481	1491	4.041	0.018 ± 0.015 ^b^	0.038 ± 0.020 ^a^	0.016 ± 0.013 ^b^	0.009 ± 0.006 ^b^	0.013 ± 0.008 ^b^
184	*trans*-2-Hexenoic acid	23:39.0	00:00.8	MS, LRI	1967	1967	3.651	0.081 ± 0.047 ^b^	0.205 ± 0.094 ^a^	0.159 ± 0.107 ^ab^	0.083 ± 0.045 ^b^	0.266 ± 0.213 ^a^
185	Nonanoic acid	26:51.5	00:00.8	S, MS, LRI	>2100	2119	3.641	0.096 ± 0.049 ^b^	0.169 ± 0.095 ^b^	0.094 ± 0.043 ^b^	0.166 ± 0.156 ^b^	0.313 ± 0.078 ^a^
186	*trans*-3-Hexenoic acid	22:49.8	00:00.8	MS, LRI	1924	1929	3.190	0.031 ± 0.033 ^a^	0.006 ± 0.005 ^b^	0.007 ± 0.003 ^b^	0.005 ± 0.003 ^b^	0.011 ± 0.005 ^ab^
187	Formic acid	15:10.4	00:00.7	MS, LRI	1508	1501	2.526	1.442 ± 0.465	2.092 ± 0.849	1.523 ± 0.393	1.176 ± 0.507	1.306 ± 0.515
188	3,5,5-Trimethylhexanoic acid	23:46.0	00:00.8	MS	1973	-	2.194	0.330 ± 0.053	0.347 ± 0.115	0.370 ± 0.113	0.437 ± 0.094	0.479 ± 0.101
189	2-Propenoic acid	17:42.7	00:00.7	MS	1645	-	2.118	0.245 ± 0.073	0.262 ± 0.101	0.266 ± 0.042	0.214 ± 0.039	0.146 ± 0.055
190	Heptanoic acid	23:22.2	00:00.8	S, MS, LRI	1953	1955	1.423	0.071 ± 0.022	0.075 ± 0.044	0.083 ± 0.076	0.060 ± 0.021	0.152 ± 0.139
191	2-Decenoic acid	15:43.7	00:00.8	MS, LRI	1536	1540	1.289	0.021 ± 0.022	0.012 ± 0.024	0.005 ± 0.010	0.025 ± 0.011	0.022 ± 0.025
192	Pentanoic acid	19:34.0	00:00.8	S, MS, LRI	1751	1751	1.006	0.408 ± 0.074	0.490 ± 0.147	0.395 ± 0.074	0.394 ± 0.063	0.513 ± 0.338
193	Isobutyric acid	16:18.0	00:00.7	S, MS, LRI	1565	1555	0.832	3.347 ± 0.988	4.725 ± 1.518	4.212 ± 2.107	3.795 ± 1.757	3.200 ± 2.305
194	*trans*,*trans*-2,4-Hexadienoic acid	26:51.6	00:00.8	MS, LRI	>2100	2150	0.753	0.188 ± 0.145	0.053 ± 0.066	18.360 ± 48.172	4.350 ± 10.563	2.236 ± 3.744
195	Isovaleric acid	18:18.8	00:00.7	S, MS, LRI	1683	1680	0.745	5.833 ± 1.482	3.926 ± 3.916	5.923 ± 2.842	6.130 ± 2.208	5.222 ± 2.990
196	2-Ethylhexanoic acid	23:18.0	00:00.8	MS, LRI	1949	1960	0.568	0.602 ± 1.256	0.232 ± 0.224	0.312 ± 0.271	0.144 ± 0.061	0.151 ± 0.019
197	Butyric acid	17:28.0	00:00.7	S, MS, LRI	1629	1626	0.546	18.241 ± 2.608	19.305 ± 5.118	17.622 ± 2.372	16.638 ± 0.905	18.144 ± 6.443
	*Esters*											
198	Methyl octanoate	12:34.0	00:01.5	MS, LRI	1401	1404	12.568	51.242 ± 14.675 ^a^	14.588 ± 8.497 ^b^	25.349 ± 13.750 ^b^	21.407 ± 4.375 ^b^	12.261 ± 13.962 ^b^
199	*cis*-3-Hexen-1-yl acetate	10:56.0	00:01.3	MS, LRI	1324	1300	12.068	20.041 ± 8.968 ^b^	39.339 ± 11.050 ^a^	22.891 ± 14.489 ^b^	6.372 ± 2.659 ^c^	4.576 ± 3.341 ^c^
200	Methyl hexanoate	07:51.1	00:05.4	S, MS, LRI	1183	1188	10.455	6.240 ± 2.416 ^a^	2.417 ± 1.319 ^b^	3.001 ± 1.098 ^b^	2.216 ± 0.469 ^b^	1.222 ± 1.089 ^b^
201	Butyl hexanoate	13:05.2	00:01.7	S, MS, LRI	1422	1428	10.423	0.059 ± 0.026 ^a^	0.015 ± 0.014 ^b^	0.017 ± 0.016 ^b^	0.015 ± 0.008 ^b^	0.008 ± 0.007 ^b^
202	Isoamyl hexanoate	14:05.0	00:01.8	S, MS, LRI	1462	1458	9.888	7.130 ± 1.573 ^a^	1.804 ± 1.100 ^c^	3.941 ± 3.105 ^b^	3.323 ± 0.756 ^bc^	1.537 ± 1.597 ^c^
203	Ethyl 3-nonenoate	16:44.2	00:01.6	MS	1587	-	7.481	0.067 ± 0.040 ^a^	0.021 ± 0.020 ^b^	0.011 ± 0.005 ^b^	0.017 ± 0.015 ^b^	0.006 ± 0.007 ^b^
204	Hexanodibutyrin	12:20.0	00:01.7	MS	1390	-	7.472	0.444 ± 0.181 ^a^	0.571 ± 0.245 ^a^	0.180 ± 0.173 ^b^	0.179 ± 0.103 ^b^	0.705 ± 0.336 ^a^
205	Methyl decanoate	16:53.0	00:01.6	MS, LRI	1594	1593	7.131	1.375 ± 0.608 ^a^	0.552 ± 0.226 ^b^	0.741 ± 0.248 ^b^	0.599 ± 0.132 ^b^	0.426 ± 0.345 ^b^
206	Phenethyl formate	20:29.8	00:01.1	MS, LRI	1799	1806	6.718	0.088 ± 0.035 ^c^	0.211 ± 0.070 ^a^	0.156 ± 0.046 ^b^	0.119 ± 0.043 ^bc^	0.112 ± 0.032 ^bc^
207	Ethyl *trans*-4-octenoate	15:15.0	00:01.5	MS	1512	-	6.256	0.031 ± 0.003 ^a^	0.017 ± 0.009 ^b^	0.023 ± 0.006 ^b^	0.016 ± 0.007 ^b^	0.017 ± 0.006 ^b^
208	Ethyl methyl succinate	17:36.4	00:01.1	MS, LRI	1638	1631	6.026	0.347 ± 0.156 ^a^	0.306 ± 0.126 ^a^	0.242 ± 0.078 ^a^	0.079 ± 0.067 ^b^	0.254 ± 0.070 ^a^
209	Octyl formate	16:04.2	00:01.0	MS, LRI	1553	1560	5.909	6.604 ± 0.947 ^b^	6.267 ± 1.160 ^b^	6.531 ± 1.465 ^b^	9.177 ± 1.345 ^a^	6.050 ± 2.280 ^b^
210	*trans*-3-Hexen-1-yl acetate	10:42.0	00:01.3	MS, LRI	1313	1316	5.675	20.322 ± 7.276 ^a^	10.073 ± 8.187 ^b^	5.922 ± 3.176 ^b^	10.530 ± 4.255 ^b^	7.388 ± 8.444 ^b^
211	Ethyl hexadecanoate	30:13.7	00:01.5	MS, LRI	>2100	2261	5.586	0.560 ± 0.414 ^a^	0.051 ± 0.082 ^b^	0.119 ± 0.222 ^b^	0.091 ± 0.149 ^b^	0.052 ± 0.064 ^b^
212	Ethyl 2-hydroxy-4-methylpentanoate	15:49.3	00:01.0	MS	1541	-	5.543	0.380 ± 0.161 ^c^	0.829 ± 0.571 ^bc^	1.875 ± 0.999 ^a^	1.625 ± 1.112 ^ab^	0.266 ± 0.231 ^c^
213	2-Phenylethyl isobutyrate	22:45.7	00:01.1	MS, LRI	1921	1916	5.419	3.788 ± 1.880 ^a^	1.824 ± 1.708 ^b^	1.421 ± 1.243 ^b^	0.816 ± 0.397 ^b^	0.769 ± 0.245 ^b^
214	Propyl hexanoate	10:56.2	00:01.7	MS, LRI	1324	1319	4.982	0.760 ± 0.330 ^a^	0.339 ± 0.286 ^b^	0.286 ± 0.227 ^b^	0.379 ± 0.143 ^b^	0.170 ± 0.180 ^b^
215	Diethyl glutarate	20:23.0	00:01.2	MS, LRI	1793	1780	4.706	0.028 ± 0.012 ^c^	0.105 ± 0.061 ^a^	0.064 ± 0.043 ^bc^	0.059 ± 0.013 ^bc^	0.092 ± 0.020 ^ab^
216	3-Ethoxypropyl acetate	11:52.0	00:01.2	MS	1368	-	4.423	0.754 ± 0.514 ^b^	2.176 ± 1.442 ^a^	0.638 ± 0.941 ^b^	0.442 ± 0.365 ^b^	0.454 ± 0.707 ^b^
217	Isoamyl octanoate	18:06.1	00:01.9	MS, LRI	1669	1657	4.033	4.133 ± 0.747 ^a^	2.340 ± 0.822 ^b^	3.155 ± 1.362 ^ab^	3.789 ± 0.950 ^a^	2.125 ± 1.656 ^b^
218	Ethyl heptanoate	11:17.0	00:01.6	MS, LRI	1341	1342	3.762	3.007 ± 1.131 ^a^	1.591 ± 1.355 ^b^	1.701 ± 0.709 ^b^	1.498 ± 0.355 ^b^	0.938 ± 1.126 ^b^
219	Ethyl pyruvate	09:53.4	00:01.0	MS, LRI	1276	1276	3.717	1.711 ± 0.693 ^b^	3.025 ± 0.908 ^a^	1.931 ± 1.095 ^b^	1.496 ± 0.650 ^b^	1.685 ± 0.421 ^b^
220	Isoamyl decanoate	21:58.0	00:01.9	MS, LRI	1879	1871	3.685	0.039 ± 0.093 ^b^	0.191 ± 0.114 ^ab^	0.180 ± 0.153 ^ab^	0.239 ± 0.042 ^ab^	0.178 ± 0.115 ^ab^
221	Propyl octanoate	15:22.0	00:01.8	MS, LRI	1518	1504	3.637	0.893 ± 0.277 ^a^	0.428 ± 0.431 ^b^	0.384 ± 0.269 ^b^	0.577 ± 0.297 ^ab^	0.270 ± 0.261 ^b^
222	Ethyl 4-pyrazolecarboxylate	14:33.0	00:01.1	MS	1481	-	3.291	0.016 ± 0.002 ^b^	0.026 ± 0.011 ^a^	0.014 ± 0.010 ^b^	0.012 ± 0.008 ^b^	0.010 ± 0.009 ^b^
223	Methyl 2-methyllactate	16:39.0	00:01.2	MS	1582	-	3.049	0.116 ± 0.015 ^a^	0.088 ± 0.013 ^bc^	0.102 ± 0.017 ^ab^	0.109 ± 0.024 ^ab^	0.068 ± 0.059 ^c^
224	3-Hydroxy-2,4,4-trimethylpentyl isobutyrate	21:54.7	00:01.2	MS	1876	-	3.046	0.089 ± 0.019 ^b^	0.191 ± 0.051 ^a^	0.124 ± 0.108 ^b^	0.104 ± 0.035 ^b^	0.112 ± 0.044 ^b^
225	Isobutyl hexanoate	11:45.0	00:01.8	MS, LRI	1362	1357	2.919	1.039 ± 0.567 ^a^	0.392 ± 0.297 ^b^	0.767 ± 0.580 ^ab^	0.595 ± 0.202 ^ab^	0.259 ± 0.272 ^b^
226	Hydroxyl acid ester (n.i.; *m/z* 143, 115, 75)	17:49.0	00:01.3	MS	1651	-	2.680	0.005 ± 0.005	0.012 ± 0.005	0.009 ± 0.006	0.007 ± 0.004	0.014 ± 0.005
227	Ethyl 4-hydroxybutyrate	20:47.7	00:00.9	MS, LRI	1815	1819	2.518	0.955 ± 0.717	1.134 ± 0.677	1.141 ± 0.695	0.315 ± 0.171	0.536 ± 0.197
228	Octyl acetate	14:24.4	00:01.5	MS, LRI	1475	1475	2.454	0.408 ± 0.857	1.601 ± 1.141	1.423 ± 1.416	3.153 ± 1.639	6.579 ± 10.545
229	3-Methyl-3-buten-1-yl acetate	08:07.8	00:01.2	MS, LRI	1196	1190	2.448	0.072 ± 0.037	0.066 ± 0.036	0.032 ± 0.028	0.049 ± 0.021	0.025 ± 0.026
230	Ethyl 2-octenoate	16:04.0	00:01.5	MS, LRI	1553	1557	2.386	0.031 ± 0.005	0.027 ± 0.009	0.024 ± 0.008	0.019 ± 0.003	0.027 ± 0.015
231	Ethyl undecanoate	18:45.2	00:01.0	MS, LRI	1709	1725	2.352	0.539 ± 0.749	0.198 ± 0.111	0.319 ± 0.391	1.006 ± 0.777	0.166 ± 0.169
232	Pentyl acetate	07:36.1	00:01.3	S, MS, LRI	1172	1161	2.334	0.123 ± 0.199	0.088 ± 0.204	0.037 ± 0.056	0.282 ± 0.174	0.053 ± 0.057
233	Isobutyl octanoate	15:57.7	00:01.9	MS, LRI	1548	1551	2.226	0.217 ± 0.071	0.119 ± 0.083	0.185 ± 0.114	0.213 ± 0.109	0.074 ± 0.069
234	Ethyl 4-hexenoate	10:21.3	00:01.4	MS, LRI	1297	1292	2.211	7.705 ± 10.236	2.044 ± 1.364	1.287 ± 0.650	0.784 ± 0.458	1.363 ± 1.374
235	Ethyl *trans*-2-butenoate	07:19.0	00:05.0	MS, LRI	1159	1161	2.127	8.551 ± 4.382	5.204 ± 3.185	7.463 ± 2.841	4.376 ± 2.710	4.293 ± 2.995
236	*trans*,*trans*-2,4-Octadien-1-yl acetate	16:18.0	00:01.4	MS	1565	-	1.956	0.017 ± 0.017	0.024 ± 0.018	0.006 ± 0.010	0.007 ± 0.009	0.017 ± 0.017
237	2-Phenylethyl octanoate	32:17.5	00:01.3	MS, LRI	>2100	2373	1.762	0.032 ± 0.036	0.006 ± 0.005	0.009 ± 0.004	0.024 ± 0.030	0.004 ± 0.004
238	Isoamyl butyrate	09:45.8	00:01.7	S, MS, LRI	1270	1266	1.756	2.573 ± 0.919	2.131 ± 0.957	2.055 ± 0.892	1.912 ± 0.358	1.089 ± 1.161
239	Di-isobutyl acetate	20:02.0	00:01.2	MS	1775	-	1.724	0.206 ± 0.065	0.243 ± 0.211	0.139 ± 0.106	0.112 ± 0.037	0.094 ± 0.032
240	3-Methylheptyl acetate	12:26.5	00:01.6	MS	1395	-	1.665	0.367 ± 0.347	0.236 ± 0.216	0.182 ± 0.086	0.112 ± 0.050	0.124 ± 0.077
241	Diethyl malonate	16:32.0	00:01.1	MS, LRI	1577	1574	1.648	0.174 ± 0.052	0.188 ± 0.079	0.148 ± 0.050	0.126 ± 0.033	0.197 ± 0.034
242	Heptyl acetate	12:13.4	00:01.5	MS, LRI	1385	1385	1.487	0.236 ± 0.133	0.249 ± 0.179	0.151 ± 0.091	0.120 ± 0.044	0.143 ± 0.145
243	Ethyl hydrogen succinate	30:16.2	00:00.8	MS, LRI	>2100	2350	1.443	3.279 ± 0.920	7.386 ± 6.573	5.875 ± 1.785	4.985 ± 2.470	4.242 ± 1.226
244	Methyl 2-isopropoxypropanoate	20:30.0	00:01.3	MS	1799	-	1.288	0.030 ± 0.042	0.076 ± 0.036	0.061 ± 0.043	0.065 ± 0.045	0.053 ± 0.047
245	Vinyl decanoate	19:27.2	00:01.4	MS	1745	-	1.258	0.224 ± 0.357	0.086 ± 0.065	0.044 ± 0.038	0.038 ± 0.034	0.130 ± 0.062
246	Diethyl malate	25:29.4	00:00.9	MS, LRI	2063	2065	1.210	0.227 ± 0.144	0.462 ± 0.502	0.399 ± 0.220	0.360 ± 0.231	0.629 ± 0.235
247	*trans*-Penten-1-yl acetate	08:50.0	00:01.2	MS	1228	-	1.132	0.050 ± 0.049	0.039 ± 0.049	0.024 ± 0.031	0.011 ± 0.011	0.024 ± 0.023
248	Phenylmethyl acetate	19:27.0	00:01.2	MS, LRI	1745	1747	1.119	0.042 ± 0.026	0.211 ± 0.394	0.035 ± 0.018	0.046 ± 0.018	0.054 ± 0.021
249	Ethyl 3-hydroxybutyrate	15:15.5	00:00.9	MS, LRI	1512	1512	1.045	0.398 ± 0.278	0.407 ± 0.202	0.430 ± 0.195	0.233 ± 0.157	0.430 ± 0.093
250	Isoamyl propanoate	07:56.8	00:01.5	MS, LRI	1188	1188	1.017	0.638 ± 0.264	0.677 ± 0.436	0.740 ± 0.490	0.543 ± 0.341	0.250 ± 0.186
251	Ethyl hydroxyacetate	13:09.0	00:00.8	MS, LRI	1424	1436	0.987	0.032 ± 0.045	0.097 ± 0.079	0.058 ± 0.076	0.085 ± 0.144	0.163 ± 0.215
252	Ethyl nonanoate	15:43.0	00:01.7	MS, LRI	1536	1535	0.928	1.843 ± 0.503	0.984 ± 0.394	0.971 ± 0.345	2.321 ± 3.355	1.116 ± 0.807
253	2-(1,1-Dimethylethyl)-cyclohexen-1-yl acetate	16:11.0	00:01.8	MS	1559	-	0.848	0.046 ± 0.026	0.024 ± 0.015	0.045 ± 0.031	0.040 ± 0.032	0.034 ± 0.004
254	Ethyl 2-hydroxy-3-phenylpropanoate	29:11.1	00:01.0	MS, LRI	>2100	2273	0.846	0.001 ± 0.001	0.001 ± 0.001	0.009 ± 0.019	0.007 ± 0.014	0.001 ± 0.000
255	Ethyl 3-methylbutylbutanedioate	22:29.7	00:01.3	MS, LRI	1907	1907	0.835	2.315 ± 0.806	3.353 ± 2.324	2.743 ± 1.814	2.843 ± 0.908	1.639 ± 0.545
256	Ethyl 9-decenoate	18:45.0	00:01.6	S, MS, LRI	1708	1708	0.801	0.133 ± 0.189	0.177 ± 0.227	0.280 ± 0.347	0.101 ± 0.086	0.067 ± 0.058
257	Ethyl 3-ethoxy-*trans*-2-propenoate	15:43.0	00:01.2	MS	1536	-	0.785	1.418 ± 0.096	1.363 ± 0.205	1.480 ± 0.136	1.542 ± 0.317	1.360 ± 0.353
258	Butyl ethyl succinate	20:37.0	00:01.3	MS, LRI	1806	1820	0.776	0.245 ± 0.112	0.295 ± 0.195	0.294 ± 0.182	0.220 ± 0.139	0.136 ± 0.063
259	Ethyl 2,4-hexadienoate I	14:35.0	00:01.3	MS, LRI	1483	1501	0.673	0.033 ± 0.034	0.019 ± 0.011	0.869 ± 2.269	0.343 ± 0.855	0.097 ± 0.153
260	Ethyl 2,4-hexadienoate II	15:08.0	00:01.3	MS, LRI	1506	1501	0.645	0.290 ± 0.328	0.038 ± 0.016	6.200 ± 16.289	3.473 ± 8.685	0.353 ± 0.515
261	Isobutyl acetate	04:23.8	00:04.7	S, MS, LRI	1015	1009	0.531	0.726 ± 0.371	0.774 ± 0.280	0.728 ± 0.435	0.534 ± 0.175	0.658 ± 0.405
262	2-Phenylethyl isovalerate	24:00.0	00:01.4	MS, LRI	1985	1988	0.530	0.015 ± 0.012	0.014 ± 0.014	0.013 ± 0.010	0.020 ± 0.006	0.011 ± 0.010
263	Methyl 2-hydroxybutanoate	11:45.0	00:01.1	MS, LRI	1362	1382	0.529	0.274 ± 0.056	0.217 ± 0.155	0.208 ± 0.156	0.280 ± 0.040	0.225 ± 0.197
264	Isopropyl lactate	15:36.0	00:01.6	MS	1530	-	0.512	0.032 ± 0.007	0.027 ± 0.004	0.029 ± 0.016	0.026 ± 0.007	0.023 ± 0.024
265	Ethyl *cis*-4-decenoate	18:17.4	00:01.6	MS, LRI	1681	1680	0.510	0.017 ± 0.019	0.012 ± 0.010	0.008 ± 0.014	0.015 ± 0.011	0.011 ± 0.009
266	Ethyl *cis*-4-octenoate	14:39.6	00:01.5	MS	1486	-	0.503	0.127 ± 0.073	0.094 ± 0.054	0.123 ± 0.053	0.098 ± 0.015	0.101 ± 0.094
267	Ethyl 2-hexenoate	11:31.7	00:01.5	S, MS, LRI	1352	1357	0.469	1.626 ± 0.984	1.864 ± 0.759	2.107 ± 1.213	1.408 ± 0.806	1.721 ± 1.542
268	Isoamyl lactate	16:18.0	00:01.0	MS, LRI	1565	1572	0.376	0.449 ± 0.304	0.829 ± 1.062	0.597 ± 0.380	0.703 ± 0.581	0.596 ± 0.341
269	Ethyl 2-propynoate	09:11.0	00:01.8	MS	1244	-	0.368	4.550 ± 1.065	4.324 ± 1.444	4.768 ± 1.504	4.322 ± 1.565	3.562 ± 2.475
270	Diethyl fumarate	17:56.0	00:01.2	MS, LRI	1659	1660	0.155	0.055 ± 0.027	0.047 ± 0.018	0.048 ± 0.031	0.050 ± 0.014	0.045 ± 0.014
	*Volatile phenols*											
271	2-Methoxyphenol	21:47.0	00:00.9	MS, LRI	1869	1869	5.084	0.023 ± 0.016 ^b^	0.052 ± 0.034 ^a^	0.019 ± 0.011 ^b^	0.010 ± 0.001 ^b^	0.011 ± 0.003 ^b^
272	4-Vinylguaiacol	27:33.5	00:00.9	S, MS, LRI	>2100	2168	2.970	0.563 ± 0.266 ^ab^	0.762 ± 0.426 ^a^	0.373 ± 0.170 ^b^	0.377 ± 0.330 ^b^	0.163 ± 0.069 ^b^
273	Phenol	24:14.0	00:00.8	S, MS, LRI	1998	1995	1.607	0.701 ± 0.049	0.856 ± 0.131	0.804 ± 0.309	0.601 ± 0.130	0.886 ± 0.541
274	4-Ethylguaiacol	24:44.7	00:01.0	S, MS, LRI	2024	2024	1.514	0.011 ± 0.020	0.024 ± 0.034	0.004 ± 0.004	0.002 ± 0.005	0.003 ± 0.003
275	2,4-Bis(1,1-dimethylethyl)phenol	29:01.0	00:01.0	MS, LRI	>2100	2270	1.328	0.923 ± 0.417	0.821 ± 0.247	1.133 ± 0.583	0.667 ± 0.280	0.810 ± 0.189
	*Furanoids and lactones*											
276	3-Methyl-2(5H)-furanone	19:13.3	00:01.0	MS, LRI	1733	1726	7.776	0.029 ± 0.012 ^a^	0.024 ± 0.011 ^ab^	0.017 ± 0.009 ^b^	0.004 ± 0.004 ^c^	0.017 ± 0.006 ^ab^
277	Acetylfuran	15:01.2	00:01.0	MS, LRI	1501	1501	4.958	0.092 ± 0.054 ^b^	0.416 ± 0.326 ^a^	0.201 ± 0.104 ^b^	0.086 ± 0.040 ^b^	0.092 ± 0.031 ^b^
278	γ-Butyrolactone	17:28.0	00:01.0	MS, LRI	1629	1626	4.491	3.839 ± 1.256 ^a^	4.284 ± 1.527 ^a^	4.055 ± 0.852 ^a^	1.918 ± 1.271 ^b^	2.661 ± 0.844 ^ab^
279	2-(5-Methyl-5-vinyltetrahydro-2-furanyl)-2-propanol	14:16.1	00:01.2	MS	1470	-	4.293	0.050 ± 0.089 ^b^	0.106 ± 0.063 ^b^	0.053 ± 0.063 ^b^	0.100 ± 0.084 ^b^	0.305 ± 0.233 ^a^
280	γ-Nonalactone	24:42.6	00:01.2	S, MS, LRI	2022	2018	3.909	0.037 ± 0.072 ^c^	0.311 ± 0.222 ^a^	0.152 ± 0.123 ^bc^	0.200 ± 0.100 ^ab^	0.191 ± 0.089 ^abc^
281	Lactone (n.i.; *m/z* 85, 57, 100)	23:39.0	00:01.1	MS	1967	-	2.450	0.057 ± 0.059	0.013 ± 0.014	0.039 ± 0.037	0.010 ± 0.006	0.009 ± 0.001
282	Pantolactone	24:42.0	00:00.8	MS, LRI	2022	2029	2.220	0.091 ± 0.018	0.184 ± 0.075	0.174 ± 0.101	0.156 ± 0.067	0.123 ± 0.046
283	Furfuryl ether	10:14.0	00:01.1	MS	1292	-	2.138	0.159 ± 0.187	0.242 ± 0.117	0.245 ± 0.100	0.091 ± 0.033	0.116 ± 0.071
284	Furfural	14:05.2	00:00.9	S, MS, LRI	1462	1460	2.087	1.122 ± 0.381	16.951 ± 26.840	2.258 ± 1.044	0.857 ± 0.168	1.010 ± 0.306
285	Ethyl 2-furoate	17:21.0	00:01.1	MS, LRI	1622	1624	1.823	4.805 ± 1.288	7.089 ± 2.513	4.929 ± 2.119	5.484 ± 1.375	4.786 ± 1.742
286	γ-Octalactone	22:49.8	00:01.1	S, MS, LRI	1924	1923	1.722	0.533 ± 0.208	0.907 ± 0.309	0.708 ± 0.325	0.723 ± 0.299	0.730 ± 0.048
287	2(5H)-furanone	19:59.4	00:00.9	S, MS, LRI	1773	1787	1.266	0.079 ± 0.013	0.151 ± 0.172	0.078 ± 0.013	0.060 ± 0.010	0.079 ± 0.046
288	5-Methyl-2-furfural	16:25.8	00:01.0	S, MS, LRI	1571	1570	1.263	0.014 ± 0.014	1.971 ± 4.278	0.055 ± 0.053	0.033 ± 0.013	0.027 ± 0.033
289	Lactone (n.i.; *m/z* 99, 71, 87)	23:41.8	00:01.2	MS	1970	-	1.206	0.012 ± 0.022	0.121 ± 0.223	0.022 ± 0.036	0.025 ± 0.022	0.040 ± 0.034
290	γ-Hydroxymethyl-γ-butyrolactone	28:33.0	00:00.9	MS	>2100	-	0.725	1.594 ± 1.045	1.857 ± 1.170	2.753 ± 1.813	2.442 ± 1.912	1.735 ± 1.520
291	5-Ethoxydihydro-2(3H)-furanone	19:20.0	00:01.0	MS, LRI	1739	1728	0.666	0.054 ± 0.024	0.059 ± 0.031	0.071 ± 0.032	0.046 ± 0.027	0.062 ± 0.039
292	δ-Caprolactone	20:37.4	00:01.1	MS, LRI	1806	1818	0.121	0.344 ± 0.225	0.332 ± 0.181	0.357 ± 0.160	0.305 ± 0.174	0.288 ± 0.108
	*Sulfur containing compounds*											
293	Methional	14:02.7	00:01.0	MS, LRI	1461	1461	11.821	0.018 ± 0.014 ^c^	0.116 ± 0.047 ^a^	0.060 ± 0.043 ^b^	0.017 ± 0.005 ^c^	0.027 ± 0.031 ^bc^
294	2-(Methylthio)ethanol	15:29.0	00:00.8	S, MS, LRI	1524	1531	9.501	0.261 ± 0.063 ^b^	0.356 ± 0.069 ^a^	0.290 ± 0.094 ^ab^	0.133 ± 0.020 ^c^	0.237 ± 0.103 ^b^
295	Methionol	19:08.8	00:00.9	S, MS, LRI	1729	1733	5.647	2.344 ± 0.660 ^bc^	4.022 ± 1.550 ^a^	3.056 ± 1.076 ^ab^	1.741 ± 0.881 ^c^	1.465 ± 0.676 ^c^
296	Ethyl thiophene-2-carboxylate	20:02.0	00:01.2	MS	1775	-	4.883	0.024 ± 0.006 ^a^	0.020 ± 0.003 ^ab^	0.018 ± 0.005 ^bc^	0.017 ± 0.004 ^bc^	0.012 ± 0.002 ^c^
297	4-(Methylthio)-1-butanol	21:26.0	00:00.9	MS	1850	-	4.672	0.011 ± 0.005 ^bc^	0.022 ± 0.010 ^a^	0.018 ± 0.009 ^ab^	0.009 ± 0.004 ^c^	0.007 ± 0.003 ^c^
298	S-(3-hydroxypropyl) thioacetate	14:47.0	00:01.1	MS	1491	-	4.320	0.052 ± 0.015 ^b^	0.083 ± 0.032 ^a^	0.062 ± 0.016 ^b^	0.048 ± 0.006 ^b^	0.042 ± 0.020 ^b^
299	2-Thiophenecarboxaldehyde	18:45.0	00:01.0	S, MS, LRI	1708	1701	3.796	0.039 ± 0.017 ^b^	0.087 ± 0.046 ^a^	0.063 ± 0.036 ^ab^	0.032 ± 0.013 ^b^	0.069 ± 0.020 ^ab^
300	Ethyl methanesulfonate	18:31.0	00:00.9	MS	1695	-	3.638	0.177 ± 0.028 ^a^	0.157 ± 0.037 ^a^	0.159 ± 0.030 ^a^	0.120 ± 0.023 ^b^	0.163 ± 0.029 ^a^
301	Ethyl 3-(methylthio)-*trans*-2-propenoate	19:41.0	00:01.2	MS, LRI	1757	1733	2.915	0.013 ± 0.007 ^b^	0.021 ± 0.008 ^a^	0.012 ± 0.004 ^b^	0.018 ± 0.003 ^ab^	0.010 ± 0.007 ^b^
302	3-(Methylthio)propyl acetate	17:28.7	00:01.2	MS, LRI	1630	1627	2.832	0.589 ± 0.319 ^ab^	0.731 ± 0.337 ^a^	0.385 ± 0.305 ^bc^	0.427 ± 0.061 ^bc^	0.202 ± 0.087 ^c^
303	Ethyl 3-(methylthio)-*trans*-2-propenoate	21:33.0	00:01.2	MS, LRI	1856	1837	2.795	0.002 ± 0.004 ^b^	0.011 ± 0.006 ^a^	0.006 ± 0.005 ^ab^	0.010 ± 0.006 ^a^	0.005 ± 0.006 ^ab^
304	Diethyl sulfate	17:21.0	00:01.0	MS	1622	-	2.496	0.013 ± 0.003	0.010 ± 0.004	0.011 ± 0.003	0.009 ± 0.002	0.011 ± 0.002
305	Ethyl thiocyanate	21:05.2	00:01.2	MS	1831	-	2.399	0.250 ± 0.090	0.299 ± 0.119	0.279 ± 0.086	0.251 ± 0.073	0.112 ± 0.045
306	3-Ethoxythiophene	14:19.0	00:01.2	MS	1472	-	1.656	0.030 ± 0.015	0.042 ± 0.035	0.054 ± 0.039	0.023 ± 0.005	0.020 ± 0.018
307	S-[(2,5-dihydro-4-hydroxy-5-oxo-3-furanyl)methyl] ethanethioate	23:09.7	00:00.8	MS	1942	-	1.376	0.113 ± 0.149	0.032 ± 0.055	0.048 ± 0.057	0.030 ± 0.024	0.016 ± 0.020
308	1-(*tert*-Butylsulfonyl)-2-octanol	19:14.9	00:02.2	MS	1734	-	0.996	0.182 ± 0.125	0.193 ± 0.116	0.220 ± 0.078	0.160 ± 0.086	0.086 ± 0.078
309	Cyclohexyl isothiocyanate	18:17.0	00:01.6	MS, LRI	1681	1667	0.780	0.014 ± 0.011	0.017 ± 0.011	0.017 ± 0.009	0.014 ± 0.008	0.006 ± 0.005
310	3-[(2-Hydroxyethyl)thio]-1-propanol	20:58.0	00:00.9	MS	1825	-	0.515	0.020 ± 0.004	0.019 ± 0.006	0.027 ± 0.023	0.029 ± 0.026	0.022 ± 0.009
311	2-Methyldihydro-3(2H)-thiophenone	15:28.6	00:01.1	MS, LRI	1523	1538	0.510	1.630 ± 0.556	1.418 ± 0.673	1.130± 0.911	1.449 ± 0.752	1.235 ± 0.462
	*Other compounds*											
312	2,6,10,10-Tetramethyl-1-oxaspiro[4.5]deca-3,6-diene	15:50.0	00:01.9	MS	1541	-	7.995	0.139 ± 0.058 ^a^	0.025 ± 0.012 ^c^	0.076 ± 0.036 ^b^	0.054 ± 0.020 ^bc^	0.075 ± 0.072 ^bc^
313	Ethylene diglycol monoethyl ether	17:19.0	00:00.9	MS, LRI	1620	1622	5.688	0.081 ± 0.031 ^b^	0.238 ± 0.114 ^a^	0.175 ± 0.095 ^a^	0.227 ± 0.062 ^a^	0.261 ± 0.017 ^a^
314	Acetic formic anhydride	15:59.6	00:00.7	MS	1549	-	1.865	0.065 ± 0.090	0.159 ± 0.132	0.151 ± 0.102	0.059 ± 0.084	0.040 ± 0.053
315	Crotonic anhydride	21:25.8	00:01.5	MS	1850	-	1.557	0.077 ± 0.060	0.063 ± 0.042	0.043 ± 0.023	0.065 ± 0.018	0.020 ± 0.017
316	1H-indole	31:00.2	00:00.9	MS, LRI	>2100	2420	1.036	0.044 ± 0.012	0.041 ± 0.019	0.037 ± 0.021	0.027 ± 0.005	0.035 ± 0.026
317	Methylsuccinic anhydride	18:59.0	00:00.9	MS	1720	-	0.517	0.013 ± 0.021	0.051 ± 0.112	0.038 ± 0.028	0.029 ± 0.026	0.053 ± 0.009

ID—identification of compounds; S—retention time and mass spectrum consistent with that of the pure standard and with NIST05 mass spectra electronic library; LRI—linear retention index consistent with that found in literature; MS—mass spectra consistent with that from NIST 2.0, Wiley 8, and FFNSC 2 mass spectra electronic libraries or literature; n.i.—not identified. The compounds with only MS symbol in ID column were tentatively identified. LRI_lit_—linear retention index from the literature, LRI_exp_—linear retention index obtained experimentally. Varieties: MI—Malvazija istarska, PO—Pošip, MA—Maraština, KR—Kraljevina, SK—Škrlet. Different superscript lowercase letters in a row represent statistically significant differences between mean values at *p* < 0.05 obtained by one-way ANOVA and least significant difference (LSD) test.

## Supplementary Materials

The following are available online at https://www.mdpi.com/2304-8158/9/12/1787/s1, Figure S1: Example of a contour plot obtained for Malvazija istarska monovarietal wine using HS-SPME/GC×GC-TOF-MS. Colored areas represent more abundant volatile aroma compounds and black dots represent less abundant and trace volatile aroma compounds, Table S1: Physico-chemical parameters in in Croatian monovarietal wines, Table S2: Concentrations (μg/L) of volatile aroma compounds found in individual Croatian monovarietal wines after headspace solid-phase microextraction followed by gas chromatography-mass spectrometry (HS-SPME/GC–MS) sorted by compound class, Table S3: Concentrations (μg/L relative to internal standard 2-octanol) of volatile aroma compounds found in individual Croatian monovarietal wines obtained by headspace solid-phase microextraction combined with comprehensive two-dimensional gas chromatography-mass spectrometry with time-of-flight mass spectrometric detection (HS-SPME/GC×GC-TOF-MS) sorted by compound class.
