# Supplementary material for: Comprehensive 2D Gas Chromatography with TOF-MS Detection Confirms the Matchless Discriminatory Power of Monoterpenes and Provides In-Depth Volatile Profile Information for Highly Efficient White Wine Varietal Differentiation
Source: Foods. 2020 Dec 2;9(12):1787. doi: 10.3390/foods9121787 (PMC7759857; doi:10.3390/foods9121787)
Supplement: Supplementary file 1 [file foods-09-01787-s001.zip › Supplementary file Figure S1 - Lukic et al 2020.docx]

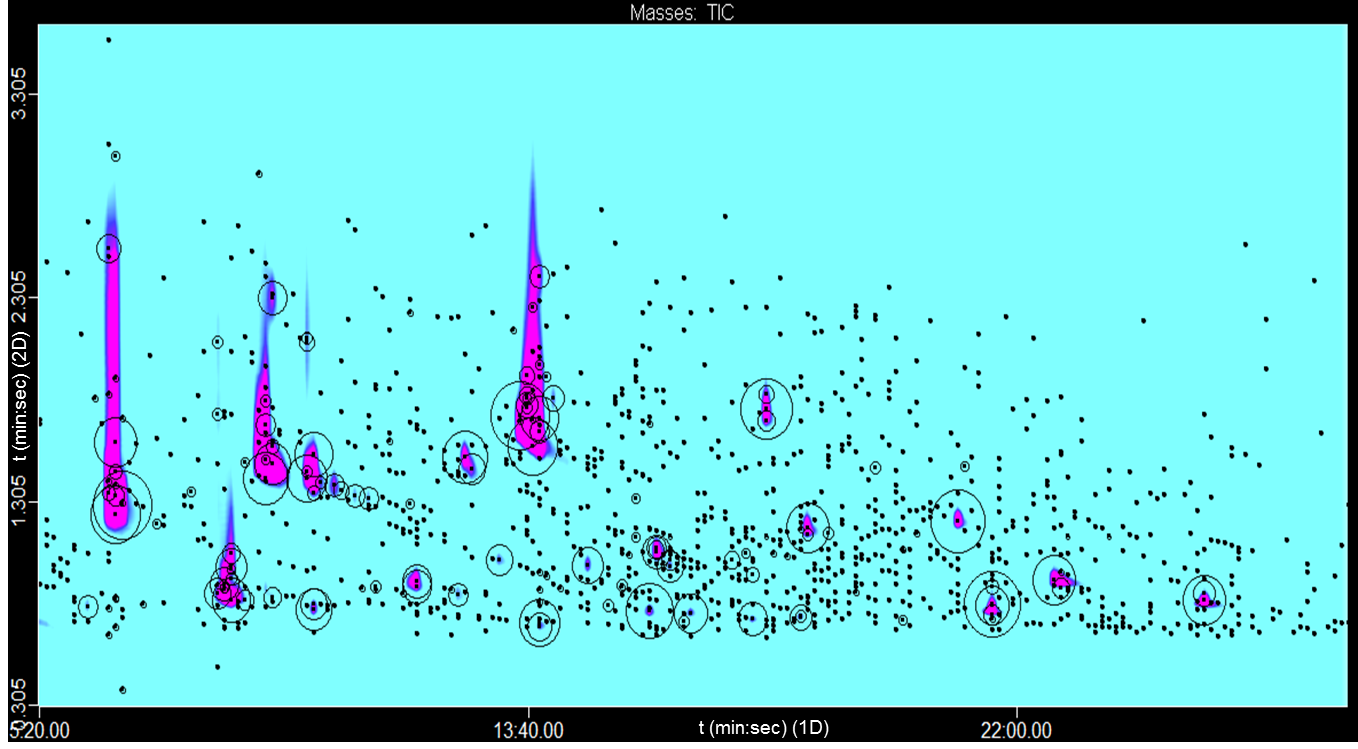


**Figure S1.** Example of a contour plot obtained for monovarietal Malvazija istarska wine using HS-SPME/GC×GC-TOF-MS. Colored areas represent more abundant volatile aroma compounds and black dots represent less abundant and trace volatile aroma compounds.
