# Supplementary material for: Comprehensive 2D Gas Chromatography with TOF-MS Detection Confirms the Matchless Discriminatory Power of Monoterpenes and Provides In-Depth Volatile Profile Information for Highly Efficient White Wine Varietal Differentiation
Source: Foods. 2020 Dec 2;9(12):1787. doi: 10.3390/foods9121787 (PMC7759857; doi:10.3390/foods9121787)
Supplement: Supplementary file 1 [file foods-09-01787-s001.zip › Supplementary file Table S1 - Lukic et al 2020.docx]

**Table S1.** Physico-chemical parameters in in Croatian monovarietal wines

| **Physico-chemical parameter** | **Variety** | | | | |
| --- | --- | --- | --- | --- | --- |
|  | **MI** | **PO** | **MA** | **KR** | **SK** |
| Specific gravity 20 °C/20 °C | 0.9906 ± 0.0009 ^c^ | 0.9920 ± 0.0008 ^a^ | 0.9914 ± 0.0008 ^ab^ | 0.9919 ± 0.0004 ^a^ | 0.9907 ± 0.0010 ^bc^ |
| Alcoholic strength (vol. %) | 13.2 ± 0.7 ^a^ | 13.1 ± 0.3 ^a^ | 12.9 ± 0.8 ^ab^ | 11.2 ± 0.3 ^c^ | 12.3 ± 0.2 ^b^ |
| Total acidity (as mg/L tartaric acid) | 5.0 ± 0.3 | 5.7 ± 0.6 | 5.4 ± 0.5 | 5.5 ± 0.3 | 5.3 ± 0.4 |
| Volatile acidity (as mg/L acetic acid) | 0.27 ± 0.08 | 0.35 ± 0.09 | 0.28 ± 0.09 | 0.25 ± 0.08 | 0.26 ± 0.08 |
| Reducing sugars (g/L) | 2.2 ± 0.4 ^b^ | 2.5 ± 0.4 ^ab^ | 2.7 ± 0.7 ^a^ | 1.5 ± 0.4 ^c^ | 1.5 ± 0.3 ^c^ |
| Total dry extract (g/L) | 20.2 ± 1.2 ^bc^ | 23.6 ± 2.5 ^a^ | 21.7 ± 0.7 ^b^ | 17.7 ± 0.7 ^d^ | 19.0 ± 1.1 ^cd^ |
| Total dry extract without reducing sugars (g/L) | 19.0 ± 1.0 ^b^ | 22.1 ± 2.3 ^a^ | 20.0 ± 0.6 ^b^ | 17.2 ± 0.3 ^c^ | 18.5 ± 0.8 ^bc^ |
| pH | 3.46 ± 0.09 ^a^ | 3.46 ± 0.09 ^a^ | 3.33 ± 0.14 ^b^ | 3.18 ± 0.05 ^c^ | 3.16 ± 0.10 ^c^ |

Varieties: MI – Malvazija istarska, PO – Pošip, MA – Maraština, KR – Kraljevina, SK – Škrlet. Different superscript lowercase letters in a row present

statistically significant differences between mean values at *p* < 0.05 obtained by one-way ANOVA and Fisher’s least significant difference (LSD) test
