# Supplementary material for: Comprehensive 2D Gas Chromatography with TOF-MS Detection Confirms the Matchless Discriminatory Power of Monoterpenes and Provides In-Depth Volatile Profile Information for Highly Efficient White Wine Varietal Differentiation
Source: Foods. 2020 Dec 2;9(12):1787. doi: 10.3390/foods9121787 (PMC7759857; doi:10.3390/foods9121787)
Supplement: Supplementary file 1 [file foods-09-01787-s001.zip › Supplementary file Table S2 - Lukic et al 2020.pdf]

Table S2. Concentrations (μg/L) of volatile aroma compounds found in individual Croatian monovarietal wines after headspace solid-phase microextraction followed by gas chromatography-mass spectrometry (HS-SPME/GC–MS) sorted by compound class

					Malvazija istarska							
**Compound**	**Group**	**ID**	**tR**	**LRIexp**	**MI-1**	**MI-2**	**MI-3**	**MI-4**	**MI-5**	**MI-6**	**MI-7**	**MI-8**
β-pinene	terpene	MS, LRI	13,75	1146	5,41	5,05	1,05	2,64	8,55	3,27	2,84	6,39
limonene	terpene	MS, LRI	15,28	1191	1,65	1,56	0,42	1,01	2,67	1,03	0,84	1,46
trans-ocimene	terpene	MS, LRI	18,05	1252	1,87	1,83	0,37	0,96	3,23	1,16	0,90	2,34
α-terpinolene	terpene	MS, LRI	19,57	1287	0,61	0,58	0,12	0,36	1,06	0,34	0,28	0,57
monoterpenyl acetate (n.i.)	terpene	MS	21,92	1302	5,76	3,67	0,26	1,45	5,37	3,01	2,31	3,11
trans- rose oxide	terpene	MS, LRI	23,38	1352	0,33	0,21	0,27	0,29	0,24	0,23	0,16	0,43
monoterpene (n.i.)	terpene	MS	28,49	1441	1,36	1,38	0,29	0,67	1,88	0,86	0,74	1,07
nerol oxide	terpene	MS, LRI	29,30	1459	3,48	5,19	1,28	2,85	3,62	2,80	2,66	2,45
cis-linalool furan oxide	terpene	MS, LRI	29,40	1464	0,11	0,10	0,08	0,10	0,06	0,04	0,05	0,06
monoterpene (n.i.)	terpene	MS	29,93	1476	0,82	0,63	0,11	0,20	0,90	0,45	0,38	0,47
monoterpene (n.i.)	terpene	MS, LRI	31,49	1509	3,38	2,00	1,85	2,07	2,54	2,68	2,61	2,46
geranyl ethyl ether	terpene	MS, LRI	31,90	1511	0,53	1,01	0,08	0,12	0,89	0,42	0,49	0,69
linalool	terpene	S, MS, LRI	33,17	1542	100,15	82,06	21,71	49,51	103,22	57,93	52,13	77,28
4-terpineol	terpene	MS, LRI	35,62	1594	0,32	0,10	0,42	0,13	0,23	0,23	0,31	0,21
ho-trienol	terpene	MS, LRI	36,03	1601	8,79	14,04	1,55	6,27	9,16	9,07	7,96	2,79
α-terpineol	terpene	S, MS, LRI	39,98	1684	22,56	21,19	5,55	10,64	25,75	11,98	10,70	16,82
trans-linalool pyran oxide	terpene	MS, LRI	41,82	1726	0,08	0,06	0,08	0,11	0,08	0,06	0,08	0,08
citronellol	terpene	S, MS, LRI	43,19	1758	5,16	5,81	4,41	4,38	5,54	5,69	4,36	4,79
nerol	terpene	S, MS, LRI	44,59	1791	21,31	15,59	3,52	7,84	18,52	10,52	8,51	21,25
geraniol	terpene	S, MS, LRI	46,59	1838	50,47	45,93	12,29	26,57	45,67	29,62	30,49	84,06
geranyl acetone	terpene	MS, LRI	47,02	1845	3,00	2,88	3,19	2,80	3,59	2,73	3,51	1,73
trans-nerolidol	terpene	MS, LRI	54,65	2031	2,24	3,20	2,43	2,37	2,96	3,22	3,69	3,01
vitispirane I	norisoprenoid	MS, LRI	32,14	1521	0,07	0,06	0,17	0,06	0,08	0,13	0,05	0,12
vitispirane II	norisoprenoid	MS, LRI	32,27	1523	0,06	0,04	0,10	0,05	0,06	0,10	0,05	0,10
actinidol ethyl ether I	norisoprenoid	MS, LRI	40,42	1690	0,15	0,21	0,47	0,18	0,24	0,12	0,20	0,39
actinidol ethyl ether II	norisoprenoid	MS, LRI	41,81	1723	0,09	0,13	0,28	0,10	0,14	0,07	0,12	0,23
β-damascenone	norisoprenoid	MS, LRI	45,43	1809	3,47	2,83	3,04	2,48	4,02	3,66	4,34	4,31
actinidol I	norisoprenoid	MS, LRI	49,91	1914	0,10	0,10	0,17	0,07	0,12	0,07	0,10	0,20
β-ionone	norisoprenoid	S, MS, LRI	50,28	1923	0,06	0,05	0,08	0,06	0,05	0,08	0,06	0,07
actinidol II	norisoprenoid	MS, LRI	50,45	1927	0,17	0,19	0,30	0,13	0,21	0,12	0,17	0,33
benzaldehyde	benzenoid	S, MS, LRI	31,43	1508	1,44	0,63	4,33	1,63	0,62	1,38	0,77	2,48
ethyl cinnamate	benzenoid	S, MS, LRI	57,55	2111	0,21	0,60	0,33	0,35	0,26	0,31	0,42	0,78
1-hexanol	alcohol	S, MS, LRI	23,59	1356	970,29	534,66	1040,67	601,20	801,14	1192,32	768,67	428,18
trans-3-hexen-1-ol	alcohol	S, MS, LRI	24,05	1361	34,86	45,80	105,29	34,34	59,84	72,77	79,29	58,80
cis-3-hexen-1-ol	alcohol	S, MS, LRI	25,05	1379	39,16	59,25	146,84	44,24	109,22	115,25	63,09	42,83
2-phenylethanol	alcohol	S, MS, LRI	48,87	1891	21667,74	21798,49	13923,67	14710,36	24388,53	24739,26	24374,56	14773,09
butyric acid	acid	S, MS, LRI	36,46	1612	1927,51	1556,07	1510,34	1708,54	1358,71	1815,02	1837,33	2415,25
hexanoic acid	acid	S, MS, LRI	46,16	1830	5370,66	5578,25	5751,74	5429,90	5014,14	6092,73	5639,38	6843,91
octanoic acid	acid	S, MS, LRI	54,93	2043	3939,77	5440,75	3482,26	3687,82	3379,00	4566,63	4566,74	5289,57
decanoic acid	acid	S, MS, LRI	62,82	2257	529,46	985,44	587,18	488,87	490,79	583,60	654,29	848,57
ethyl butyrate	ester	S, MS, LRI	9,45	1030	453,32	402,63	369,82	501,10	372,07	496,67	500,36	558,70
ethyl 2-methylbutyrate	ester	S, MS, LRI	10,00	1049	2,96	5,69	4,62	3,07	4,75	4,01	5,52	2,90
ethyl 3-methylbutyrate	ester	S, MS, LRI	10,51	1065	6,23	11,74	7,65	7,42	9,07	8,48	10,91	6,58
isoamyl acetate	ester	S, MS, LRI	12,49	1120	3456,35	2210,15	1793,86	4471,13	2527,20	3389,48	3561,74	4983,04
ethyl hexanoate	ester	S, MS, LRI	17,59	1236	643,42	531,22	1103,52	771,03	643,23	761,95	689,14	629,28
hexyl acetate	ester	S, MS, LRI	19,43	1272	219,85	178,08	182,69	199,66	228,84	316,75	256,08	151,13
ethyl lactate	ester	S, MS, LRI	22,93	1341	28572,32	13905,68	26316,10	18741,23	56319,00	13895,97	21866,83	27930,19
ethyl octanoate	ester	S, MS, LRI	28,10	1435	1237,00	879,36	1201,46	1463,44	1064,45	1392,21	1517,92	932,46
ethyl decanoate	ester	S, MS, LRI	37,72	1637	291,23	394,38	290,21	251,75	260,70	280,76	345,93	305,67
diethyl succinate	ester	S, MS, LRI	39,07	1667	1486,31	1714,87	1657,72	1618,67	2130,39	1716,59	1965,67	786,47
2-phenethyl acetate	ester	S, MS, LRI	45,05	1803	2079,95	2063,74	1314,07	2001,39	2361,67	2837,84	2725,42	2456,39

	Pošip							Maraština						
**Compound**	**PO-1**	**PO-2**	**PO-3**	**PO-4**	**PO-5**	**PO-6**	**PO-7**	**MA-1**	**MA-2**	**MA-3**	**MA-4**	**MA-5**	**MA-6**	**MA-7**
β-pinene	1,75	3,23	3,36	2,91	1,51	1,85	2,38	0,43	0,22	0,44	0,46	0,41	0,64	0,20
limonene	0,61	1,15	1,21	1,13	0,57	0,93	1,25	0,25	0,15	0,20	0,19	0,14	0,25	0,14
trans-ocimene	0,90	1,69	1,81	1,40	0,71	0,99	1,36	0,17	0,09	0,18	0,19	0,17	0,28	0,08
α-terpinolene	0,25	2,80	0,54	0,46	0,21	0,36	0,48	0,08	0,03	0,04	0,05	0,07	0,16	0,03
monoterpenyl acetate (n.i.)	1,16	1,62	1,12	1,18	1,05	0,86	0,76	0,77	0,26	0,48	0,79	0,29	1,24	0,32
trans- rose oxide	0,15	0,17	0,22	0,18	0,27	0,20	0,24	0,28	0,12	0,15	0,05	0,15	0,15	0,12
monoterpene (n.i.)	0,41	0,87	0,52	0,55	0,42	1,05	0,63	0,76	0,04	0,29	0,26	0,08	0,30	0,14
nerol oxide	2,86	3,22	2,67	3,80	1,75	7,37	4,52	3,77	0,13	0,89	1,00	1,03	1,81	0,82
cis-linalool furan oxide	0,21	0,18	0,10	0,25	0,13	0,26	0,11	0,13	0,02	0,04	0,04	0,02	0,13	0,04
monoterpene (n.i.)	0,16	0,34	0,22	0,29	0,21	0,34	0,21	0,27	0,42	0,09	0,09	0,53	0,16	0,11
monoterpene (n.i.)	2,38	3,27	2,67	2,22	2,45	1,39	2,46	3,18	1,73	2,88	1,16	2,02	1,49	2,33
geranyl ethyl ether	1,93	2,56	0,27	0,31	0,37	0,39	0,19	0,43	1,33	2,23	0,09	1,44	0,19	1,83
linalool	40,42	51,11	37,99	44,91	28,10	33,10	31,58	23,40	7,51	20,15	18,99	13,83	32,94	12,78
4-terpineol	0,25	0,32	0,22	0,28	0,19	0,25	0,15	0,33	0,06	0,35	0,12	0,22	0,20	0,33
ho-trienol	6,72	7,44	6,65	7,33	3,08	9,87	7,56	2,60	1,50	0,93	1,69	0,60	3,80	0,85
α-terpineol	7,63	14,56	8,94	13,32	7,20	14,38	10,37	7,78	4,77	5,09	3,35	3,44	8,91	5,16
trans-linalool pyran oxide	0,11	0,06	0,19	0,14	0,07	0,17	0,17	0,09	0,02	0,07	0,00	0,05	0,12	0,12
citronellol	5,62	5,05	5,97	3,93	4,55	5,48	5,01	5,80	7,50	2,43	3,70	6,12	6,80	4,76
nerol	7,48	10,26	6,06	6,61	5,84	4,67	4,50	4,16	3,75	3,32	5,65	3,94	7,67	3,39
geraniol	18,90	38,83	23,71	30,06	28,89	14,90	14,36	11,46	148,32	27,90	18,63	17,33	24,18	31,93
geranyl acetone	4,80	3,22	2,36	4,69	2,39	3,93	3,65	2,33	8,23	8,45	2,24	4,68	14,20	12,91
trans-nerolidol	2,82	4,18	2,68	3,02	2,49	3,38	3,62	3,02	1,02	5,97	2,09	2,21	2,36	1,97
vitispirane I	0,18	0,35	0,20	0,44	0,76	0,74	0,54	0,36	0,09	0,42	0,22	0,25	0,17	0,82
vitispirane II	0,15	0,27	0,17	0,31	0,55	0,54	0,38	0,24	0,06	0,23	0,16	0,16	0,17	0,38
actinidol ethyl ether I	0,13	0,34	0,19	0,38	0,64	0,78	0,56	0,66	0,07	0,51	0,27	0,20	0,07	0,62
actinidol ethyl ether II	0,08	0,11	0,11	0,22	0,39	0,47	0,34	0,39	0,04	0,30	0,15	0,12	0,04	0,36
β-damascenone	1,91	3,47	2,50	2,43	5,72	2,08	1,54	1,64	1,89	1,44	2,19	2,18	3,11	1,50
actinidol I	0,07	0,16	0,09	0,17	0,23	0,20	0,19	0,18	0,05	0,21	0,10	0,13	0,06	0,21
β-ionone	0,06	0,07	0,05	0,04	0,04	0,05	0,04	0,07	0,05	0,07	0,06	0,06	0,07	0,09
actinidol II	0,12	0,27	0,16	0,30	0,23	0,20	0,32	0,30	0,10	0,35	0,17	0,21	0,12	0,34
benzaldehyde	1,91	1,38	1,28	1,51	15,72	1,24	1,33	1,58	0,71	1,40	0,61	2,11	0,90	0,90
ethyl cinnamate	0,92	0,88	2,35	1,06	0,47	0,32	2,12	0,32	0,35	0,51	0,47	0,31	0,36	0,40
1-hexanol	776,85	1255,20	862,18	798,24	854,47	1123,99	978,61	1162,11	818,56	905,64	767,50	684,24	695,99	980,02
trans-3-hexen-1-ol	51,66	74,69	62,68	26,58	39,01	29,69	35,14	57,45	44,48	43,99	53,90	48,04	52,04	26,10
cis-3-hexen-1-ol	257,46	210,64	194,89	401,66	506,97	260,56	263,14	95,24	151,02	209,49	458,11	144,36	110,60	183,59
2-phenylethanol	36803,58	36275,15	36075,14	30215,55	23977,72	35789,03	33096,70	26767,08	47082,88	48285,45	23629,98	24477,88	30288,49	24288,67
butyric acid	1827,70	1795,27	1694,92	1248,91	1690,37	1309,80	1682,64	1675,90	1030,57	1257,91	1992,76	1783,33	2181,45	1875,93
hexanoic acid	5866,60	6125,83	4946,15	4148,49	5630,87	4527,07	5047,53	3984,23	3122,85	3965,24	5962,66	8089,98	6306,31	5560,54
octanoic acid	6814,74	7884,91	7048,85	3534,14	5249,63	5602,26	7543,62	3350,48	2690,15	5690,32	5466,56	6757,75	5405,46	6669,88
decanoic acid	1976,90	1738,33	2151,70	554,91	870,09	1808,91	2292,40	621,57	300,16	1581,76	1447,29	1135,95	783,89	1568,34
ethyl butyrate	396,21	431,63	450,07	369,77	492,79	342,29	425,33	379,63	215,81	299,18	374,72	395,08	450,39	428,24
ethyl 2-methylbutyrate	7,76	8,31	6,27	4,38	3,05	9,79	6,43	9,56	6,27	10,35	7,11	3,55	3,39	5,74
ethyl 3-methylbutyrate	16,02	16,24	13,40	8,87	6,90	22,64	19,48	17,28	10,92	20,49	15,28	6,72	6,48	12,82
isoamyl acetate	2186,59	6523,08	4112,59	3689,66	2892,38	1458,65	2386,65	782,68	983,07	2563,24	1588,59	1382,49	1456,72	1469,68
ethyl hexanoate	365,59	421,32	303,74	357,35	555,90	336,66	314,86	466,17	325,67	254,34	477,24	574,00	718,41	428,10
hexyl acetate	157,32	343,60	237,94	130,90	193,79	142,42	225,15	60,18	70,38	157,79	184,29	105,13	92,66	192,30
ethyl lactate	15841,02	172308,76	24756,62	29533,19	29129,37	27308,84	21833,27	37217,55	29753,43	27993,02	21497,93	63791,31	14483,00	46498,34
ethyl octanoate	796,88	1382,82	961,19	1063,42	1301,47	1228,67	871,11	825,87	834,44	559,31	591,36	721,89	1236,47	950,18
ethyl decanoate	243,21	219,60	273,81	249,67	419,07	318,68	235,63	323,00	120,44	115,99	92,14	155,85	214,03	232,26
diethyl succinate	582,65	4399,95	856,30	3014,05	1424,45	2011,96	1132,43	3421,48	1856,47	1191,91	749,23	1442,64	1754,83	1238,65
2-phenethyl acetate	4705,92	7348,31	5940,98	4168,52	3045,45	3601,42	4307,78	921,76	2312,45	5030,32	2516,46	2121,62	1604,23	2006,69

	Kraljevina							Škrlet		
**Compound**	**KR-1**	**KR-2**	**KR-3**	**KR-4**	**KR-5**	**KR-6**	**KR-7**	**SK-1**	**SK-2**	**SK-3**
β-pinene	1,07	1,00	0,96	0,94	0,88	1,32	1,92	4,42	5,09	3,01
limonene	0,31	0,35	0,26	0,34	0,30	0,35	0,59	2,18	3,82	1,98
trans-ocimene	0,50	0,48	0,45	0,43	0,43	0,63	0,94	1,22	1,84	0,84
α-terpinolene	0,10	0,12	0,10	0,09	0,09	0,27	0,21	0,56	0,05	0,40
monoterpenyl acetate (n.i.)	0,10	0,06	0,11	0,07	0,19	0,12	0,23	4,05	3,04	2,39
trans- rose oxide	0,11	0,15	0,15	0,15	0,15	0,20	0,19	0,98	0,46	0,34
monoterpene (n.i.)	0,00	0,00	0,00	0,00	0,00	0,05	0,12	2,89	3,74	1,72
nerol oxide	0,62	0,77	1,32	0,99	1,07	1,13	1,86	4,57	5,27	2,50
cis-linalool furan oxide	0,03	0,02	0,01	0,02	0,03	0,01	0,03	0,19	0,32	0,10
monoterpene (n.i.)	0,27	0,05	0,02	0,35	0,04	0,03	0,07	0,83	0,98	0,51
monoterpene (n.i.)	1,00	1,26	0,82	1,19	1,15	1,10	1,25	6,09	1,07	1,46
geranyl ethyl ether	0,05	0,02	0,06	0,06	0,09	0,03	0,06	0,67	1,11	0,67
linalool	6,39	7,73	6,01	6,51	6,02	7,61	13,23	105,48	92,15	74,62
4-terpineol	0,17	0,09	0,14	0,09	0,09	0,09	0,14	0,48	0,60	0,45
ho-trienol	1,02	1,06	1,74	1,28	1,36	1,51	3,22	4,97	4,17	3,40
α-terpineol	1,56	2,20	1,22	1,68	1,56	1,78	3,90	41,18	51,18	29,11
trans-linalool pyran oxide	0,08	0,02	0,02	0,03	0,02	0,10	0,02	0,08	0,06	0,04
citronellol	2,18	2,72	2,41	2,77	2,29	3,05	2,49	7,62	4,53	4,65
nerol	0,95	0,67	0,83	0,85	0,83	1,26	2,03	20,58	18,24	13,26
geraniol	2,14	2,33	2,85	4,49	5,13	0,97	1,24	55,46	48,37	34,75
geranyl acetone	3,44	2,68	2,37	2,24	2,58	2,41	2,73	3,80	2,30	1,56
trans-nerolidol	1,63	1,51	1,31	2,03	1,54	1,47	1,65	1,54	1,87	1,17
vitispirane I	0,13	0,22	0,13	0,27	0,19	0,20	0,20	0,25	0,53	0,19
vitispirane II	0,07	0,10	0,09	0,11	0,09	0,10	0,10	0,11	0,21	0,10
actinidol ethyl ether I	0,11	0,13	0,09	0,12	0,09	0,11	0,08	0,17	0,29	0,25
actinidol ethyl ether II	0,07	0,08	0,06	0,08	0,05	0,07	0,05	0,10	0,19	0,15
β-damascenone	2,49	2,30	1,75	2,26	2,40	2,32	2,41	0,99	0,56	1,12
actinidol I	0,05	0,06	0,04	0,05	0,04	0,04	0,03	0,07	0,12	0,10
β-ionone	0,05	0,04	0,05	0,05	0,06	0,05	0,06	0,09	0,06	0,07
actinidol II	0,09	0,10	0,07	0,09	0,06	0,08	0,06	0,11	0,20	0,17
benzaldehyde	2,38	1,20	2,69	1,86	3,29	3,28	3,26	2,67	1,81	4,86
ethyl cinnamate	0,22	0,13	0,14	0,12	0,16	0,27	0,39	0,10	0,10	0,27
1-hexanol	273,69	320,19	340,43	313,52	290,06	367,17	348,15	1396,22	1547,24	1688,79
trans-3-hexen-1-ol	46,84	32,37	53,19	45,42	36,11	57,01	30,72	89,81	47,02	54,77
cis-3-hexen-1-ol	25,97	22,05	34,67	27,06	27,14	26,21	20,02	31,88	52,80	79,31
2-phenylethanol	16184,79	20029,56	12136,63	21751,01	20412,92	22492,58	31980,28	18406,29	18141,06	16450,42
butyric acid	1539,21	1730,89	1650,83	1644,19	1551,91	1749,70	1203,98	2182,84	1646,27	1535,86
hexanoic acid	7484,78	6214,50	6461,13	6453,34	6004,04	7054,95	5742,51	8106,41	7068,95	5900,99
octanoic acid	6828,88	6376,49	5664,22	5597,62	6212,75	6612,20	6243,80	6629,65	7353,14	5096,39
decanoic acid	1094,56	1048,99	952,96	891,66	957,45	1000,16	1014,50	860,77	1658,42	751,89
ethyl butyrate	377,99	347,53	408,52	369,97	363,55	425,56	277,98	436,32	316,33	300,32
ethyl 2-methylbutyrate	5,07	3,67	4,55	9,57	4,91	6,12	9,89	3,65	3,66	4,73
ethyl 3-methylbutyrate	8,71	7,84	8,34	14,06	9,19	11,21	20,03	7,99	7,77	9,40
isoamyl acetate	3160,75	2245,08	3205,45	1920,92	2333,04	2887,98	1028,95	2522,48	1637,81	1479,13
ethyl hexanoate	747,46	450,28	658,11	584,44	517,67	677,52	428,73	597,90	394,92	432,03
hexyl acetate	124,05	114,31	145,68	81,38	107,78	136,96	45,22	241,86	162,09	217,32
ethyl lactate	16195,12	24840,34	14389,09	22371,76	21718,31	25416,99	52584,98	24974,19	33528,75	39459,86
ethyl octanoate	810,06	557,12	701,65	672,78	954,19	749,26	466,40	767,74	284,53	579,80
ethyl decanoate	243,44	190,16	230,32	259,04	229,89	212,12	175,58	166,00	207,89	223,88
diethyl succinate	692,93	1043,06	638,74	1470,63	891,31	1029,09	1216,70	972,46	1040,11	1181,08
2-phenethyl acetate	2665,35	2725,08	2125,37	2726,49	2735,55	2865,86	2215,74	2021,06	1777,87	1453,18
