# Supplementary material for: Comprehensive 2D Gas Chromatography with TOF-MS Detection Confirms the Matchless Discriminatory Power of Monoterpenes and Provides In-Depth Volatile Profile Information for Highly Efficient White Wine Varietal Differentiation
Source: Foods. 2020 Dec 2;9(12):1787. doi: 10.3390/foods9121787 (PMC7759857; doi:10.3390/foods9121787)
Supplement: Supplementary file 1 [file foods-09-01787-s001.zip › Supplementary file Table S3 - Lukic et al 2020.pdf]

Table S3. Concentrations (μg/L relative to internal standard 2-octanol) of volatile aroma compounds found in individual Croatian monovarietal wines obtained by headspace solid-phase microextraction combined with comprehensive two-dimensional gas chromatography-mass spectrometry with time-of-flight mass spectrometric detection (HS-SPME/GC×GC-TOF-MS) sorted by compound class.

						Malvazija istarska								Pošip							Maraština							Kraljevina							Škrlet		
**Compound**	**group**	**tR (1D)**	**tR (2D)**	**LRI calc**	**LRI lit**	**MI-1**	**MI-2**	**MI-3**	**MI-4**	**MI-5**	**MI-6**	**MI-7**	**MI-8**	**PO-1**	**PO-2**	**PO-3**	**PO-4**	**PO-5**	**PO-6**	**PO-7**	**MA-1**	**MA-2**	**MA-3**	**MA-4**	**MA-5**	**MA-6**	**MA-7**	**KR-1**	**KR-2**	**KR-3**	**KR-4**	**KR-5**	**KR-6**	**KR-7**	**SK-1**	**SK-2**	**SK-3**
β-myrcene	terpene	07:18.8	00:01.6	**1159**	1159	4.048	2.543	0.439	1.741	3.515	1.093	1.834	3.596	0.777	2.046	0.968	0.774	0.454	0.563	0.616	0.355	0.312	0.426	0.338	0.274	0.394	0.219	0.188	0.188	0.223	0.104	0.184	0.049	0.349	6.403	4.050	2.606
α-terpinene	terpene	07:33.6	00:01.8	**1170**	1175	0.044	0.000	0.041	0.000	0.177	0.000	0.000	0.000	0.017	0.000	0.000	0.000	0.000	0.000	0.022	0.000	0.000	0.000	0.019	0.000	0.000	0.020	0.000	0.000	0.000	0.011	0.000	0.000	0.039	0.082	0.237	0.000
limonene	terpene	08:01.0	00:01.8	**1191**	1194	1.479	0.833	0.537	0.628	2.549	1.115	1.937	2.132	0.681	0.124	0.045	0.507	0.361	0.603	0.682	0.511	0.275	0.352	0.413	0.185	0.378	0.405	0.094	0.263	0.094	0.224	0.074	0.198	0.390	3.693	4.268	3.905
monoterpene (n.i.)	terpene	08:15.2	00:01.8	**1202**		0.774	0.396	0.055	0.000	0.451	0.052	0.046	0.245	0.057	0.289	0.142	0.103	0.111	0.074	0.105	0.057	0.050	0.029	0.079	0.020	0.062	0.031	0.032	0.022	0.036	0.000	0.000	0.022	0.029	0.387	0.745	0.593
trans -β-ocimene	terpene	09:01.7	00:01.7	**1237**	1241	0.000	0.000	0.246	0.000	0.000	0.000	0.377	0.000	0.000	0.182	0.111	0.000	0.176	0.000	0.000	0.000	0.045	0.137	0.293	0.037	0.000	0.180	0.022	0.000	0.000	0.000	0.000	0.028	0.048	0.000	1.852	0.906
cis -α-ocimene	terpene	09:24.8	00:01.6	**1254**	1255	3.081	2.533	1.047	1.945	3.798	1.237	1.835	2.751	0.784	1.035	0.801	0.844	0.356	0.689	0.770	0.537	0.392	0.513	0.546	0.395	0.503	0.322	0.210	0.269	0.363	0.322	0.238	0.249	0.705	4.666	3.582	0.963
o -cymene	terpene	09:49.9	00:01.6	**1273**	1268	1.678	1.524	0.879	1.062	2.062	1.005	0.824	1.408	0.189	0.842	0.166	1.149	0.609	0.691	0.769	0.805	0.267	0.567	0.598	0.113	0.134	0.605	0.182	0.329	0.199	0.203	0.155	0.315	0.563	2.949	3.447	1.445
α-terpinolene	terpene	10:03.7	00:01.9	**1284**	1282	3.000	1.749	0.533	1.302	2.803	0.827	1.339	1.485	0.608	0.998	0.338	0.565	0.238	0.185	0.706	0.712	0.201	0.411	0.581	0.123	0.216	0.465	0.101	0.182	0.099	0.211	0.113	0.163	0.340	3.800	5.620	0.754
linalool ethyl ether	terpene	11:02.8	00:01.9	**1329**	1331	9.232	7.915	1.291	4.266	8.215	3.503	3.487	5.444	2.067	3.435	2.004	1.506	0.462	1.127	1.608	1.240	0.958	0.754	1.146	0.765	1.216	0.909	0.380	0.382	0.324	0.386	0.287	0.417	0.931	9.540	8.310	1.245
trans -alloocimene	terpene	12:13.0	00:01.6	**1384**	1388	0.453	0.341	0.137	0.202	0.743	0.252	0.323	0.443	0.186	0.166	0.098	0.127	0.120	0.111	0.152	0.157	0.036	0.085	0.133	0.045	0.071	0.089	0.023	0.050	0.029	0.041	0.021	0.036	0.084	0.710	1.054	0.494
p -cymenene	terpene	13:30.2	00:01.5	**1439**	1438	1.502	1.297	0.985	1.261	1.788	1.259	1.192	1.371	0.918	1.161	0.761	0.873	0.692	0.743	1.147	1.443	0.625	0.907	1.005	0.700	0.603	1.139	0.601	0.693	0.674	0.817	0.504	0.684	1.157	1.780	2.460	0.893
linalool furan oxide	terpene	13:39.9	00:01.2	**1445**	1450	0.467	0.487	0.220	0.568	0.515	0.416	0.335	0.541	0.368	0.479	0.241	0.427	0.732	0.508	0.608	0.477	0.072	0.187	0.212	0.173	0.086	0.340	0.053	0.124	0.000	0.275	0.062	0.202	0.385	0.705	1.176	0.735
cosmene	terpene	13:41.6	00:01.4	**1446**	1460	0.027	0.204	0.272	0.268	0.027	0.014	0.046	0.013	0.174	0.040	0.000	0.055	0.019	0.051	0.052	0.224	0.118	0.087	0.131	0.072	0.000	0.108	0.000	0.134	0.043	0.153	0.050	0.097	0.084	0.015	0.036	0.031
monoterpene (n.i.)	terpene	13:54.1	00:01.9	**1455**		16.351	14.573	3.426	8.048	16.203	8.113	7.557	10.752	4.983	7.131	4.585	3.716	0.601	5.036	4.889	6.172	2.149	2.135	2.508	1.257	2.453	2.484	0.924	0.978	0.573	1.106	0.612	0.897	2.635	20.329	26.171	2.180
neryl ethyl ether	terpene	14:26.5	00:01.8	**1477**	1468	0.544	0.489	0.066	0.231	0.543	0.224	0.244	0.327	0.150	0.235	0.120	0.115	0.030	0.192	0.159	0.228	0.072	0.072	0.077	0.032	0.068	0.151	0.016	0.013	0.009	0.027	0.012	0.018	0.068	1.448	1.096	0.047
geranyl ethyl ether	terpene	15:08.0	00:01.8	**1506**	1506	3.112	2.629	0.551	1.577	3.372	1.207	1.719	2.170	0.773	1.300	0.620	0.608	0.229	0.576	0.866	0.897	0.331	0.432	0.513	0.426	0.395	0.508	0.117	0.199	0.111	0.241	0.129	0.184	0.570	3.932	4.505	0.443
terpene n.i.	terpene	15:08.0	00:01.8	**1506**		1.022	0.798	0.742	0.811	1.164	0.616	1.166	0.813	1.034	1.237	0.720	0.634	0.439	0.379	1.048	1.189	0.541	1.158	0.520	0.802	0.268	1.040	0.319	0.596	0.215	0.577	0.395	0.404	0.701	2.079	0.327	0.104
4-thujanol	terpene	15:20.1	00:01.7	**1516**		0.048	0.042	0.138	0.000	0.043	0.035	0.052	0.083	0.113	0.052	0.041	0.048	0.040	0.019	0.046	0.075	0.000	0.094	0.090	0.131	0.046	0.036	0.084	0.079	0.036	0.091	0.085	0.031	0.068	0.047	0.032	0.057
linalool	terpene	15:50.0	00:01.1	**1541**	1541	25.792	23.758	7.316	21.459	9.650	12.740	11.761	20.834	11.417	13.374	11.510	10.898	8.739	10.970	8.390	7.021	3.700	5.016	5.099	4.654	7.871	3.864	2.770	3.113	2.636	2.147	2.191	2.237	4.905	27.317	20.350	23.356
α-bergamotene	terpene	16:18.0	00:02.4	**1565**	1585	0.021	0.022	0.040	0.024	0.025	0.014	0.047	0.021	0.025	0.064	0.017	0.035	0.078	0.000	0.038	0.029	0.030	0.205	0.047	0.008	0.000	0.090	0.041	0.057	0.014	0.036	0.025	0.045	0.072	0.046	0.065	0.017
4-terpineol	terpene	17:00.0	00:01.3	**1600**	1597	0.470	0.512	0.616	0.335	0.447	0.365	0.566	0.403	0.407	0.457	0.327	0.384	0.379	0.240	0.347	0.533	0.254	0.499	0.444	0.357	0.299	0.540	0.264	0.267	0.375	0.238	0.197	0.251	0.430	0.690	0.713	0.627
ho-trienol	terpene	17:07.0	00:01.0	**1607**	1612	3.445	4.754	0.958	2.525	3.567	2.958	3.081	1.458	2.870	2.719	2.114	2.068	1.213	2.619	2.547	1.193	0.340	0.510	0.855	0.203	1.177	0.409	0.373	0.560	0.804	0.680	0.428	0.594	1.544	2.664	2.608	2.259
carvomenthenal	terpene	17:14.0	00:01.5	**1615**	1629	0.430	0.389	0.145	0.234	0.391	0.265	0.339	0.859	0.183	0.310	0.101	0.116	0.311	0.125	0.207	0.185	0.109	0.132	0.215	0.061	0.118	0.172	0.040	0.047	0.037	0.056	0.033	0.056	0.133	0.456	0.678	0.442
β-cyclocitral	terpene	17:21.0	00:01.5	**1622**	1629	0.050	0.053	0.098	0.064	0.064	0.060	0.063	0.083	0.069	0.075	0.044	0.074	0.081	0.058	0.098	0.091	0.040	0.085	0.073	0.036	0.030	0.104	0.039	0.062	0.039	0.062	0.035	0.049	0.069	0.068	0.068	0.019
menthol	terpene	17:42.0	00:01.1	**1644**	1641	0.098	0.117	0.053	0.108	0.191	0.150	0.203	0.115	0.161	0.099	0.112	0.082	0.861	0.128	0.109	0.110	0.086	0.106	0.097	0.086	0.149	0.169	0.107	0.094	0.105	0.085	0.071	0.091	0.144	0.108	0.113	3.309
citronellyl acetate	terpene	18:10.0	00:01.5	**1673**	1668	0.037	0.096	0.007	0.027	0.087	0.071	0.055	0.065	0.140	0.115	0.138	0.011	0.007	0.045	0.117	0.014	0.043	0.035	0.062	0.042	0.018	0.074	0.037	0.072	0.032	0.024	0.022	0.032	0.025	0.119	0.070	0.000
α-farnesene I	terpene	18:17.0	00:01.9	**1681**	1697	0.059	0.085	0.198	0.122	0.125	0.116	0.149	0.156	0.127	0.093	0.046	0.050	0.053	0.043	0.109	0.105	0.049	0.211	0.126	0.065	0.046	0.111	0.093	0.148	0.028	0.156	0.058	0.114	0.207	0.136	0.142	0.059
α-terpineol	terpene	18:47.2	00:01.2	**1710**	1709	4.967	4.268	1.832	2.899	5.750	2.457	2.717	4.578	2.394	3.081	1.733	2.435	2.238	2.794	2.408	2.034	0.701	1.341	1.396	0.779	1.079	1.459	0.479	0.820	0.658	0.756	0.415	0.398	1.461	11.115	12.103	11.667
neryl acetate	terpene	19:20.7	00:01.5	**1739**	1742	0.078	0.057	0.123	0.080	0.080	0.079	0.072	0.108	0.119	0.113	0.051	0.023	0.007	0.006	0.103	0.098	0.021	0.080	0.078	0.029	0.006	0.096	0.032	0.121	0.014	0.148	0.023	0.100	0.111	0.112	0.163	0.000
linalool pyran oxide	terpene	19:34.0	00:01.1	**1751**	**1721**	0.056	0.054	0.077	0.070	0.089	0.038	0.053	0.035	0.133	0.079	0.041	0.074	0.000	0.128	0.118	0.071	0.000	0.014	0.033	0.013	0.071	0.000	0.000	0.034	0.027	0.054	0.022	0.027	0.056	0.096	0.104	0.058
sesquiterpene (n.i.)	terpene	19:48.0	00:01.8	**1763**		0.062	0.052	0.075	0.066	0.049	0.066	0.062	0.079	0.053	0.057	0.006	0.045	0.034	0.016	0.052	0.048	0.039	0.052	0.018	0.044	0.026	0.055	0.036	0.041	0.039	0.049	0.036	0.047	0.070	0.075	0.052	0.000
α-farnesene II	terpene	19:48.0	00:01.9	**1763**	1762	0.034	0.035	0.060	0.054	0.043	0.046	0.059	0.090	0.040	0.049	0.000	0.023	0.032	0.020	0.041	0.032	0.016	0.066	0.000	0.025	0.000	0.040	0.029	0.042	0.012	0.051	0.022	0.040	0.077	0.073	0.046	0.022
β-cadinene	terpene	19:55.0	00:02.1	**1769**		0.022	0.016	0.022	0.019	0.018	0.024	0.021	0.021	0.021	0.019	0.014	0.014	0.000	0.012	0.014	0.020	0.007	0.018	0.015	0.016	0.014	0.019	0.014	0.017	0.002	0.016	0.012	0.013	0.024	0.019	0.018	0.000
geranyl acetate	terpene	19:55.0	00:01.5	**1769**	1768	0.040	0.034	0.015	0.021	0.039	0.036	0.047	0.064	0.080	0.101	0.067	0.000	0.020	0.011	0.058	0.027	0.020	0.048	0.057	0.038	0.009	0.050	0.042	0.054	0.037	0.017	0.027	0.042	0.022	0.066	0.036	0.000
citronellol	terpene	20:02.9	00:01.1	**1776**	1777	0.302	0.250	0.362	0.259	0.320	0.321	0.286	0.326	0.398	0.283	0.141	0.160	0.289	0.199	0.280	0.367	0.123	0.164	0.326	0.271	0.254	0.313	0.135	0.207	0.068	0.196	0.048	0.179	0.248	0.521	0.067	0.341
sesquiterpene (n.i.)	terpene	20:16.0	00:02.0	**1787**		0.010	0.016	0.033	0.020	0.020	0.026	0.023	0.023	0.020	0.019	0.000	0.011	0.010	0.009	0.026	0.025	0.009	0.038	0.026	0.011	0.000	0.030	0.018	0.025	0.000	0.037	0.011	0.022	0.041	0.026	0.042	0.000
α-curcumene	terpene	20:16.0	00:01.8	**1787**	1782	0.016	0.014	0.058	0.020	0.020	0.025	0.026	0.031	0.030	0.026	0.011	0.016	0.014	0.015	0.024	0.023	0.010	0.032	0.023	0.014	0.019	0.026	0.015	0.036	0.008	0.041	0.015	0.033	0.054	0.021	0.034	0.011
γ-isogeraniol	terpene	20:29.2	00:01.0	**1799**	1800	0.068	0.190	0.148	0.180	0.000	0.000	0.188	0.414	0.161	0.223	0.110	0.000	0.212	0.046	0.108	0.044	0.053	0.151	0.184	0.102	0.000	0.079	0.000	0.061	0.077	0.125	0.079	0.084	0.000	0.111	0.000	0.150
nerol	terpene	20:43.5	00:01.0	**1812**	1811	0.633	0.524	0.330	0.422	1.001	0.334	0.335	0.965	0.030	0.340	0.219	0.370	0.257	0.123	0.201	0.223	0.118	0.182	0.316	0.216	0.313	0.207	0.125	0.106	0.011	0.063	0.056	0.078	0.100	0.929	0.845	0.658
monoterpene (n.i.)	terpene	20:48.1	00:01.7	**1816**		0.028	0.032	0.087	0.051	0.035	0.033	0.056	0.126	0.047	0.103	0.036	0.048	0.086	0.055	0.094	0.061	0.005	0.098	0.000	0.026	0.009	0.087	0.027	0.036	0.008	0.041	0.006	0.035	0.045	0.026	0.038	0.019
isogeraniol	terpene	20:55.4	00:01.0	**1822**	1828	0.036	0.017	0.035	0.027	0.022	0.014	0.031	0.108	0.015	0.039	0.004	0.011	0.034	0.005	0.019	0.000	0.002	0.017	0.001	0.011	0.000	0.006	0.000	0.006	0.000	0.023	0.000	0.017	0.022	0.022	0.014	0.008
trans -calamenene	terpene	21:19.0	00:01.9	**1844**	1837	0.044	0.044	0.095	0.074	0.042	0.072	0.072	0.058	0.096	0.059	0.039	0.038	0.057	0.040	0.062	0.058	0.033	0.063	0.056	0.038	0.042	0.052	0.073	0.083	0.038	0.103	0.043	0.083	0.113	0.066	0.072	0.030
geraniol	terpene	21:33.0	00:01.0	**1856**	1857	0.476	0.385	0.237	0.287	0.470	0.234	0.350	1.016	0.293	0.353	0.210	0.156	0.300	0.111	0.154	0.124	0.068	0.140	0.173	0.142	0.151	0.069	0.099	0.080	0.110	0.063	0.054	0.084	0.095	0.649	0.499	0.618
trans -geranyl acetone	terpene	21:40.0	00:01.5	**1863**	1868	0.220	0.264	1.487	0.300	0.255	0.201	0.297	0.250	0.240	0.219	0.168	0.142	0.251	0.214	0.364	0.149	0.182	0.330	0.196	0.232	0.165	0.191	0.216	0.193	1.709	0.187	0.193	0.174	0.281	0.163	0.181	0.112
α-calacorene	terpene	22:29.0	00:01.8	**1906**	1916	0.019	0.013	0.025	0.020	0.020	0.019	0.023	0.020	0.016	0.019	0.009	0.023	0.000	0.011	0.018	0.017	0.010	0.022	0.016	0.012	0.012	0.019	0.018	0.022	0.007	0.024	0.013	0.018	0.035	0.019	0.016	0.000
β-calacorene	terpene	22:43.0	00:01.8	**1919**	1918	0.087	0.081	0.122	0.124	0.094	0.114	0.122	0.118	0.079	0.082	0.090	0.077	0.071	0.057	0.069	0.096	0.065	0.075	0.063	0.084	0.083	0.080	0.145	0.143	0.050	0.182	0.098	0.139	0.160	0.115	0.087	0.015
2-acetyl-2-carene	terpene	23:11.0	00:01.3	**1943**		0.000	0.000	0.097	0.027	0.000	0.000	0.000	0.114	0.052	0.000	0.000	0.041	0.000	0.068	0.099	0.000	0.000	0.095	0.083	0.037	0.000	0.115	0.000	0.000	0.031	0.000	0.012	0.024	0.000	0.039	0.059	0.062
cis -Z-α-bisabolene epoxide	terpene	24:28.0	00:01.3	**2010**	2007	0.000	0.000	0.009	0.005	0.003	0.000	0.000	0.000	0.005	0.006	0.003	0.009	0.008	0.013	0.015	0.012	0.003	0.022	0.009	0.005	0.000	0.021	0.009	0.014	0.005	0.021	0.010	0.013	0.019	0.017	0.040	0.011
nerolidol	terpene	24:56.2	00:01.3	**2034**	2034	0.058	0.090	0.212	0.132	0.103	0.075	0.098	0.143	0.160	0.132	0.097	0.060	0.054	0.077	0.131	0.094	0.058	0.284	0.164	0.127	0.011	0.134	0.134	0.188	0.035	0.187	0.104	0.146	0.197	0.154	0.181	0.059
γ-dehydro-ar-himachalene	terpene	25:10.0	00:01.7	**2046**		0.003	0.000	0.008	0.006	0.000	0.003	0.004	0.003	0.006	0.006	0.003	0.000	0.006	0.000	0.000	0.003	0.000	0.005	0.004	0.000	0.002	0.004	0.021	0.016	0.006	0.019	0.012	0.020	0.019	0.007	0.005	0.000
cadalene	terpene	27:46.2	00:01.6	**>2100**	2191	0.035	0.040	0.050	0.051	0.037	0.054	0.064	0.042	0.073	0.044	0.033	0.034	0.037	0.015	0.039	0.032	0.034	0.039	0.036	0.043	0.013	0.045	0.053	0.068	0.027	0.088	0.059	0.063	0.089	0.044	0.044	0.007
dehydroaromadendrene	terpene	28:20.9	00:01.4	**>2100**		0.002	0.000	0.003	0.003	0.000	0.000	0.000	0.004	0.002	0.000	0.000	0.000	0.095	0.000	0.000	0.005	0.000	0.002	0.000	0.000	0.000	0.000	0.003	0.004	0.003	0.005	0.013	0.011	0.045	0.068	0.008	0.008
α-ionene	norisoprenoi	14:28.9	00:02.0	**1479**		0.074	0.041	0.048	0.035	0.041	0.019	0.064	0.041	0.008	0.022	0.010	0.000	0.067	0.000	0.013	0.146	0.076	0.065	0.093	0.078	0.026	0.000	0.096	0.077	0.053	0.085	0.091	0.052	0.143	0.014	0.033	0.049
vitispirane	norisoprenoi	15:29.0	00:01.9	**1524**	1524	0.806	0.636	1.815	0.776	1.158	1.760	0.765	1.521	2.124	2.416	1.642	3.875	3.489	5.462	5.502	4.451	3.033	4.287	3.469	2.755	1.066	9.344	1.386	2.540	1.161	3.654	1.671	1.729	3.262	2.369	5.818	0.835
3,4-dehydro-β-ionone	norisoprenoi	18:38.0	00:01.8	**1702**		0.032	0.023	0.030	0.026	0.043	0.023	0.045	0.038	0.010	0.012	0.006	0.008	0.034	0.005	0.002	0.065	0.028	0.032	0.046	0.024	0.014	0.044	0.027	0.042	0.020	0.048	0.025	0.040	0.078	0.018	0.018	0.025
actinidol ethyl ether I	norisoprenoi	18:52.0	00:01.9	**1714**	IMA	0.035	0.052	0.262	0.069	0.085	0.031	0.072	0.185	0.054	0.155	0.056	0.149	0.315	0.262	0.279	0.271	0.067	0.268	0.145	0.073	0.000	0.348	0.049	0.070	0.000	0.093	0.039	0.068	0.070	0.097	0.210	0.092
1,2-dihydro-1,1,6-trimethyl-naphtha	norisoprenoi	19:06.0	00:01.6	**1727**	1729	0.000	0.014	0.038	0.017	0.020	0.017	0.031	0.033	0.012	0.021	0.007	0.015	0.031	0.012	0.033	0.050	0.016	0.040	0.042	0.015	0.000	0.055	0.014	0.024	0.010	0.040	0.012	0.023	0.060	0.016	0.029	0.000
1,2-dihydro-1,5,8-trimethylnaphthal	norisoprenoi	19:41.0	00:01.6	**1757**		0.405	0.226	0.549	0.279	0.334	0.247	0.406	0.433	0.204	0.403	0.142	0.263	0.672	0.332	0.525	1.082	0.479	0.644	0.655	0.378	0.201	1.337	0.424	0.715	0.272	1.331	0.445	0.776	1.425	0.608	2.301	0.344
norisoprenoid (n.i.)	norisoprenoi	20:00.5	00:01.6	**1774**	1788	0.242	0.143	0.345	0.173	0.262	0.179	0.311	0.326	0.182	0.228	0.067	0.100	0.352	0.086	0.126	0.129	0.058	0.115	0.248	0.108	0.088	0.139	0.174	0.229	0.158	0.215	0.135	0.195	0.312	0.075	0.027	0.061
β-damascenone	norisoprenoi	21:05.0	00:01.5	**1831**	1832	7.841	5.541	10.836	5.990	9.027	6.218	9.601	10.909	6.105	7.452	4.392	3.600	11.576	3.265	4.000	4.107	2.495	3.766	7.565	4.291	3.668	4.475	7.083	7.644	6.266	6.927	5.473	6.633	8.843	2.525	1.986	2.497
1,2-dihydro-1,4,6-trimethylnaphthal	norisoprenoi	25:38.3	00:01.5	**2071**		0.000	0.002	0.002	0.000	0.000	0.000	0.000	0.002	0.000	0.000	0.001	0.000	0.000	0.001	0.000	0.004	0.003	0.006	0.017	0.011	0.001	0.023	0.006	0.025	0.004	0.030	0.014	0.026	0.058	0.000	0.003	0.002
1,2-dimethylbenzene	benzenoid	07:41.0	00:05.1	**1176**	1175	0.317	0.324	0.888	0.450	0.708	0.899	0.000	0.159	1.056	0.073	0.082	0.312	0.000	0.611	0.748	0.796	0.064	0.715	0.674	0.000	0.397	0.874	0.062	0.595	0.198	0.570	0.067	0.151	0.852	0.634	0.663	0.000
styrene	benzenoid	09:27.4	00:05.0	**1256**	1257	2.021	2.832	2.004	2.254	1.777	2.483	1.077	2.089	3.414	3.017	2.507	1.430	1.129	2.999	2.735	1.905	1.909	2.335	2.231	2.326	2.569	1.853	1.677	1.365	1.439	1.570	1.672	1.571	2.616	1.307	1.221	1.141
3,3-dimethoxy-1-phenylpropan-1-or	benzenoid	09:56.8	00:05.6	**1278**		0.056	0.074	0.000	0.047	0.070	0.000	0.000	0.054	0.000	0.000	0.066	0.000	0.146	0.055	0.067	0.037	0.000	0.055	0.025	0.019	0.179	0.047	0.030	0.000	0.000	0.027	0.000	0.050	0.067	0.059	0.034	0.095
α-methylstyrene	benzenoid	11:10.8	00:01.3	**1336**	1325	0.000	0.000	0.017	0.009	0.012	0.000	0.014	0.000	0.018	0.013	0.025	0.011	0.007	0.011	0.016	0.028	0.011	0.013	0.019	0.006	0.009	0.018	0.011	0.017	0.010	0.032	0.010	0.012	0.024	0.000	0.014	0.339
2-ethyl-*m* -xylene	benzenoid	11:58.5	00:01.6	**1373**	1372	0.050	0.102	0.103	0.214	0.037	0.189	0.095	0.061	0.151	0.143	0.159	0.061	0.000	0.144	0.128	0.098	0.077	0.021	0.097	0.147	0.034	0.097	0.062	0.119	0.054	0.172	0.086	0.074	0.089	0.055	0.066	0.000
trimethyl-tetrahydronaphthalene	benzenoid	13:12.1	00:01.8	**1426**		0.000	0.000	0.017	0.000	0.007	0.000	0.033	0.000	0.000	0.000	0.000	0.007	0.008	0.000	0.000	0.160	0.017	0.023	0.049	0.038	0.000	0.102	0.053	0.120	0.028	0.152	0.025	0.120	0.213	0.004	0.015	0.000
durene	benzenoid	13:16.0	00:01.5	**1429**	1435	0.076	0.079	0.083	0.100	0.114	0.117	0.013	0.096	0.108	0.118	0.081	0.073	0.003	0.097	0.112	0.124	0.052	0.072	0.104	0.048	0.085	0.078	0.038	0.070	0.054	0.082	0.000	0.044	0.104	0.027	0.065	0.010
3-methylphenylacetylene	benzenoid	14:33.0	00:01.3	**1481**	1450.9	0.014	0.012	0.017	0.011	0.012	0.011	0.010	0.012	0.023	0.013	0.010	0.016	0.010	0.024	0.015	0.016	0.010	0.018	0.020	0.000	0.010	0.021	0.024	0.032	0.026	0.033	0.023	0.027	0.061	0.030	0.040	0.015
prehnitene	benzenoid	14:40.0	00:01.5	**1486**	1476	0.226	0.206	0.203	0.156	0.237	0.187	0.138	0.229	0.170	0.205	0.122	0.133	0.102	0.136	0.249	0.286	0.215	0.269	0.230	0.188	0.156	0.399	0.096	0.118	0.107	0.133	0.119	0.098	0.194	0.154	0.193	0.024
benzofuran	benzenoid	15:01.0	00:01.1	**1500**	1496	0.028	0.034	0.032	0.036	0.058	0.037	0.060	0.030	0.064	0.084	0.048	0.038	0.033	0.081	0.081	0.072	0.028	0.053	0.054	0.032	0.035	0.052	0.033	0.043	0.029	0.046	0.025	0.038	0.064	0.032	0.033	0.024
benzaldehyde	benzenoid	15:16.8	00:05.8	**1514**	1509	1.154	0.834	3.920	1.944	1.497	1.787	1.617	2.723	4.341	1.506	1.395	1.794	19.165	1.825	2.607	1.813	0.773	1.670	1.478	1.887	1.280	1.448	1.765	2.093	1.735	1.837	2.219	2.193	3.285	1.964	1.446	5.391
benzenoid n.i.	benzenoid	15:50.0	00:01.9	**1541**		0.146	0.081	0.084	0.077	0.239	0.136	0.152	0.197	0.016	0.031	0.032	0.030	0.000	0.030	0.033	0.075	0.046	0.147	0.085	0.069	0.037	0.075	0.050	0.061	0.037	0.055	0.031	0.053	0.093	0.143	0.083	0.000
benzenoid n.i.	benzenoid	16:37.2	00:01.4	**1581**		0.000	0.007	0.006	0.005	0.010	0.008	0.010	0.006	0.007	0.008	0.004	0.011	0.005	0.000	0.000	0.000	0.006	0.008	0.009	0.003	0.000	0.007	0.007	0.020	0.012	0.014	0.007	0.019	0.017	0.012	0.009	0.007
2,3-dihydrobenzofuran	benzenoid	16:46.0	00:01.2	**1588**		0.025	0.029	0.023	0.024	0.033	0.039	0.000	0.029	0.064	0.044	0.039	0.021	0.043	0.047	0.061	0.038	0.022	0.055	0.055	0.025	0.036	0.039	0.019	0.028	0.021	0.031	0.020	0.024	0.060	0.029	0.027	0.029
benzonitrile	benzenoid	17:00.0	00:01.0	**1600**	1591	0.030	0.028	0.035	0.039	0.042	0.043	0.046	0.000	0.078	0.072	0.109	0.030	0.049	0.047	0.060	0.083	0.033	0.057	0.059	0.032	0.030	0.071	0.027	0.064	0.025	0.036	0.026	0.032	0.059	0.036	0.035	0.045
trans -edulan	benzenoid	17:07.0	00:01.8	**1607**	1602	0.032	0.027	0.045	0.029	0.043	0.027	0.036	0.076	0.080	0.115	0.058	0.086	0.094	0.083	0.071	0.064	0.030	0.065	0.080	0.051	0.033	0.070	0.033	0.046	0.032	0.048	0.033	0.044	0.059	0.045	0.059	0.000
meso -2,3-diphenylbutane	benzenoid	17:07.0	00:01.3	**1607**		0.025	0.037	0.000	0.000	0.048	0.035	0.030	0.027	0.075	0.048	0.057	0.023	0.045	0.056	0.049	0.046	0.024	0.046	0.042	0.030	0.040	0.030	0.021	0.026	0.022	0.027	0.018	0.024	0.048	0.031	0.027	0.039
2-methylbenzaldehyde	benzenoid	17:27.8	00:01.1	**1629**	1622	0.024	0.047	0.030	0.041	0.040	0.037	0.036	0.070	0.038	0.039	0.031	0.062	0.082	0.061	0.090	0.079	0.030	0.030	0.040	0.000	0.027	0.031	0.021	0.054	0.040	0.027	0.021	0.024	0.035	0.050	0.027	0.076
2,3-dihydro-1,1,5,6-tetramethyl-1H-i	benzenoid	17:28.0	00:01.7	**1629**		0.028	0.019	0.025	0.020	0.022	0.015	0.036	0.022	0.005	0.009	0.000	0.000	0.028	0.000	0.004	0.071	0.035	0.022	0.041	0.030	0.012	0.055	0.033	0.076	0.022	0.070	0.027	0.060	0.104	0.016	0.012	0.021
benzeneacetaldehyde	benzenoid	17:49.0	00:01.1	**1651**	1648	4.736	5.563	6.043	4.041	7.168	6.639	8.585	4.589	14.285	9.087	10.118	7.255	9.349	11.119	10.669	10.084	6.197	11.041	10.055	5.545	8.372	8.115	5.190	6.576	4.331	6.208	3.664	5.271	9.947	5.421	5.705	8.376
2,3-dihydro-1,1,5,6-tetramethyl-1H-i	benzenoid	18:17.0	00:01.7	**1681**		0.081	0.062	0.086	0.068	0.068	0.052	0.113	0.074	0.017	0.029	0.012	0.017	0.093	0.008	0.014	0.161	0.095	0.084	0.122	0.094	0.042	0.138	0.115	0.143	0.073	0.161	0.104	0.131	0.244	0.041	0.044	0.064
ethyl benzoate	benzenoid	18:17.0	00:01.2	**1681**	1678	0.600	0.437	1.206	0.835	0.693	0.648	0.733	0.922	2.298	1.302	1.432	1.432	1.312	1.216	1.457	0.672	0.431	0.771	0.782	0.712	0.531	0.673	0.443	0.556	0.502	0.554	0.410	0.494	0.770	0.704	0.499	0.509
3-ethylbenzaldehyde	benzenoid	18:59.4	00:01.2	**1721**	1732	0.082	0.061	0.088	0.089	0.092	0.100	0.105	0.085	0.104	0.099	0.081	0.076	0.095	0.073	0.116	0.120	0.063	0.093	0.109	0.066	0.066	0.086	0.047	0.066	0.054	0.073	0.045	0.062	0.107	0.074	0.079	0.072
octylbenzene	benzenoid	19:27.0	00:01.8	**1745**	1746	0.117	0.100	0.124	0.114	0.091	0.116	0.106	0.103	0.077	0.089	0.053	0.074	0.054	0.026	0.081	0.078	0.087	0.073	0.073	0.076	0.054	0.080	0.056	0.076	0.069	0.076	0.063	0.072	0.093	0.094	0.084	0.033
azulene	benzenoid	19:34.0	00:01.3	**1751**	1746	0.207	0.161	0.200	0.271	0.255	0.301	0.329	0.195	0.186	0.148	0.144	0.166	0.143	0.254	0.225	0.233	0.135	0.171	0.175	0.101	0.151	0.191	0.102	0.124	0.140	0.137	0.102	0.095	0.208	0.138	0.141	0.072
4-ethylbenzaldehyde	benzenoid	19:34.0	00:01.2	**1751**	1747	0.058	0.045	0.064	0.054	0.065	0.068	0.070	0.043	0.080	0.075	0.061	0.068	0.052	0.049	0.083	0.076	0.046	0.068	0.078	0.051	0.040	0.064	0.035	0.038	0.037	0.050	0.034	0.044	0.063	0.050	0.053	0.039
p -methoxyanisole	benzenoid	19:34.2	00:01.2	**1751**	1752	0.078	0.118	0.209	0.153	0.162	0.151	0.189	0.161	0.182	0.165	0.149	0.148	0.182	0.120	0.344	0.095	0.097	0.205	0.147	0.141	0.115	0.191	0.127	0.224	0.141	0.199	0.218	0.193	0.279	0.174	0.273	0.125
ethyl *o* -methylbenzoate	benzenoid	19:34.2	00:01.3	**1751**		0.043	0.038	0.039	0.042	0.037	0.028	0.042	0.043	0.045	0.048	0.039	0.034	0.037	0.040	0.044	0.026	0.029	0.035	0.037	0.039	0.037	0.036	0.000	0.032	0.029	0.032	0.026	0.031	0.044	0.039	0.033	0.011
m -methoxyanisole	benzenoid	19:48.0	00:01.2	**1763**	1761	0.027	0.000	0.028	0.000	0.000	0.000	0.000	0.017	0.037	0.036	0.062	0.000	0.058	0.031	0.021	0.029	0.000	0.015	0.016	0.006	0.000	0.000	0.000	0.000	0.027	0.028	0.016	0.021	0.000	0.062	0.022	0.126
6-[1-(hydroxymethyl)vinyl]-4,8a-dim	benzenoid	19:54.7	00:02.0	**1769**		0.000	0.000	0.012	0.015	0.000	0.041	0.000	0.012	0.008	0.013	0.010	0.000	0.033	0.000	0.010	0.026	0.000	0.011	0.000	0.012	0.000	0.014	0.040	0.036	0.014	0.069	0.013	0.041	0.071	0.018	0.021	0.000
cis -anethole	benzenoid	19:55.0	00:01.3	**1769**	1780	0.008	0.015	0.007	0.009	0.017	0.014	0.011	0.021	0.021	0.016	0.008	0.008	0.020	0.006	0.024	0.015	0.008	0.012	0.019	0.014	0.012	0.021	0.010	0.011	0.015	0.011	0.007	0.017	0.027	0.010	0.014	0.029
α,α-dimethylbenzenemethanol	benzenoid	19:55.2	00:01.0	**1769**	1770	0.023	0.023	0.027	0.022	0.021	0.029	0.022	0.000	0.038	0.029	0.071	0.021	0.026	0.022	0.023	0.038	0.018	0.023	0.025	0.013	0.023	0.029	0.020	0.038	0.018	0.026	0.024	0.029	0.034	0.027	0.022	0.560
methyl salicylate	benzenoid	20:16.0	00:01.2	**1787**	1789	1.694	1.592	3.371	2.231	5.126	4.467	5.798	3.374	3.744	2.163	1.170	1.027	18.750	1.457	3.528	4.678	0.968	2.601	2.853	1.086	1.215	3.727	1.019	3.053	1.348	2.543	1.040	1.490	3.362	1.247	3.025	39.614
ethyl benzeneacetate	benzenoid	20:30.0	00:01.2	**1799**	1788	1.333	1.129	1.737	0.997	1.164	1.251	1.877	1.187	3.419	3.483	2.305	3.336	3.276	1.869	4.521	4.505	1.069	3.464	2.895	1.903	1.159	2.525	1.427	1.681	2.380	3.057	1.741	2.360	10.411	6.075	1.606	1.655
trans -1,2-diphenylcyclobutane	benzenoid	20:58.0	00:01.3	**1825**		0.004	0.000	0.005	0.004	0.003	0.004	0.003	0.001	0.001	0.002	0.002	0.004	0.002	0.005	0.006	0.000	0.004	0.018	0.001	0.002	0.000	0.001	0.000	0.001	0.001	0.002	0.000	0.001	0.007	0.001	0.000	0.000
(+)-cuparene	benzenoid	21:05.0	00:02.0	**1831**	1830	0.020	0.018	0.020	0.020	0.020	0.022	0.021	0.019	0.022	0.021	0.000	0.014	0.000	0.016	0.023	0.023	0.015	0.018	0.020	0.013	0.014	0.019	0.013	0.016	0.006	0.000	0.012	0.013	0.024	0.017	0.018	0.000
trans -1-(2,3,6-trimethylphenyl)buta-	benzenoid	21:12.0	00:01.5	**1837**	1832	0.051	0.033	0.104	0.042	0.066	0.044	0.066	0.112	0.030	0.062	0.017	0.037	0.119	0.041	0.092	0.096	0.034	0.107	0.096	0.036	0.016	0.145	0.035	0.059	0.022	0.079	0.032	0.058	0.112	0.038	0.079	0.033
trans -anethole	benzenoid	21:12.0	00:01.3	**1837**	1834	0.268	0.331	0.026	0.476	0.687	0.404	0.477	0.711	0.672	0.400	0.294	0.330	0.691	0.180	0.575	0.233	0.171	0.322	0.444	0.408	0.326	0.822	0.307	0.276	0.175	0.203	0.218	0.548	1.036	0.316	0.534	0.990
3-methylbenzofuran	benzenoid	21:19.0	00:01.1	**1844**		0.022	0.024	0.019	0.025	0.034	0.037	0.028	0.025	0.058	0.045	0.044	0.021	0.054	0.048	0.055	0.025	0.011	0.052	0.051	0.042	0.017	0.036	0.020	0.039	0.024	0.021	0.025	0.037	0.059	0.029	0.032	0.040
ethyl salicylate	benzenoid	21:35.8	00:01.1	**1859**	1837	0.014	0.008	0.000	0.104	0.139	0.000	0.000	0.031	0.005	0.003	0.000	0.000	0.000	0.000	0.023	0.133	0.006	0.035	0.000	0.000	0.012	0.000	0.004	0.020	0.006	0.033	0.001	0.000	0.015	0.000	0.013	0.519
1-methylnapthalene	benzenoid	21:40.0	00:01.3	**1863**	1878	0.012	0.012	0.011	0.018	0.020	0.023	0.022	0.017	0.011	0.013	0.011	0.009	0.013	0.030	0.020	0.017	0.010	0.016	0.015	0.011	0.013	0.017	0.010	0.009	0.014	0.009	0.009	0.010	0.015	0.011	0.010	0.009
benzyl alcohol	benzenoid	22:01.0	00:00.9	**1881**	1877	0.428	0.293	0.560	0.544	0.236	0.420	0.429	4.400	8.564	0.950	0.770	0.404	2.152	0.319	0.890	0.246	0.316	0.498	0.986	0.381	0.411	0.437	0.370	0.328	0.424	0.400	0.213	0.438	0.304	0.410	0.377	0.908
2-methylnaphthalene	benzenoid	22:15.0	00:01.3	**1894**	1872	0.018	0.018	0.016	0.023	0.021	0.025	0.028	0.019	0.015	0.014	0.011	0.014	0.016	0.021	0.017	0.019	0.010	0.017	0.016	0.011	0.011	0.016	0.011	0.014	0.014	0.018	0.011	0.014	0.025	0.014	0.014	0.008
ethyl benzenepropanoate	benzenoid	22:15.0	00:01.3	**1894**	1892	0.304	0.272	0.296	0.898	0.330	0.180	0.239	0.711	0.702	0.970	0.261	0.934	0.318	0.109	0.269	0.161	0.278	0.578	0.557	0.138	0.247	0.236	0.161	0.253	0.151	0.251	0.146	0.216	0.265	0.222	0.096	0.075
2-(1,1-dimethylethyl)-1,4-dimethoxy	benzenoid	22:29.0	00:01.4	**1906**	1870	0.076	0.050	0.080	0.075	0.066	0.059	0.076	0.073	0.075	0.076	0.053	0.061	0.038	0.051	0.069	0.068	0.045	0.081	0.081	0.050	0.049	0.085	0.062	0.078	0.046	0.078	0.073	0.070	0.116	0.075	0.078	0.000
butylated hydroxytoluene	benzenoid	22:43.0	00:01.5	**1919**	1920	0.403	0.310	0.262	0.239	0.248	0.369	0.419	0.265	0.303	0.610	0.239	0.247	0.553	0.304	0.200	0.302	0.234	0.501	0.166	0.252	0.347	0.261	0.269	0.296	0.100	0.208	0.229	0.206	0.287	0.234	0.343	0.054
2-hydroxybenzeneacetic acid	benzenoid	23:11.0	00:01.0	**1943**		0.000	0.000	0.005	0.000	0.000	0.000	0.000	0.022	0.006	0.007	0.005	0.006	0.014	0.004	0.012	0.000	0.000	0.005	0.007	0.004	0.000	0.004	0.003	0.005	0.004	0.006	0.004	0.005	0.005	0.004	0.004	0.008
methyl 2-(benzyloxy)propanoate	benzenoid	23:18.0	00:01.3	**1949**		0.018	0.451	0.027	1.582	0.350	1.969	1.970	0.236	0.028	0.084	0.019	0.000	0.344	0.348	0.182	0.083	0.017	0.050	0.096	0.020	0.072	0.412	0.032	0.017	1.243	0.014	0.096	0.027	0.045	0.020	0.034	0.000
styralyl isobutyrate	benzenoid	23:31.8	00:01.4	**1961**		0.070	0.062	0.038	0.109	0.056	0.073	0.069	0.042	0.336	0.208	0.189	0.035	0.041	0.192	0.332	0.037	0.042	0.315	0.213	0.185	0.079	0.290	0.065	0.204	0.090	0.111	0.121	0.200	0.250	0.099	0.118	0.073
2',5'-dimethylcrotonophenone	benzenoid	24:00.0	00:01.3	**1985**		0.032	0.030	0.064	0.032	0.047	0.029	0.054	0.073	0.038	0.045	0.024	0.022	0.103	0.024	0.025	0.022	0.009	0.024	0.066	0.025	0.013	0.026	0.029	0.043	0.028	0.048	0.035	0.039	0.059	0.017	0.000	0.014
1,1'-oxybisbenzene	benzenoid	24:20.1	00:01.3	**2003**	2017	0.009	0.011	0.011	0.011	0.007	0.013	0.011	0.011	0.003	0.004	0.003	0.003	0.005	0.009	0.003	0.009	0.005	0.002	0.002	0.002	0.004	0.004	0.003	0.002	0.006	0.002	0.001	0.003	0.004	0.004	0.003	0.002
benzoic acid	benzenoid	30:48.9	00:00.8	**>2100**	2438	0.265	0.264	0.236	0.294	0.247	0.292	0.265	0.291	0.453	0.415	0.316	0.362	0.375	0.468	0.494	0.296	0.334	0.331	0.376	0.434	0.311	0.366	0.299	0.260	0.263	0.243	0.325	0.242	0.000	0.275	0.266	0.463
benzeneacetic acid	benzenoid	32:45.2	00:00.8	**>2100**	2519	0.002	0.002	0.002	0.002	0.002	0.002	0.002	0.003	0.015	0.003	0.002	0.000	0.004	0.002	0.007	0.003	0.003	0.014	0.019	0.007	0.002	0.007	0.001	0.002	0.002	0.002	0.002	0.002	0.020	0.025	0.002	0.002
cis , *trans* -1,3,5-octatriene	hydrocarbor	08:08.4	00:00.7	**1196**		0.144	0.243	0.359	0.385	0.439	0.443	0.637	0.360	0.407	0.236	0.142	0.402	0.426	0.461	0.472	0.458	0.137	0.317	0.463	0.143	0.265	0.421	0.201	0.348	0.088	0.340	0.091	0.203	0.448	0.299	0.356	0.333
2,3,3-trimethyl-*cis* -4-nonene	hydrocarbor	10:42.0	00:01.6	**1313**		0.099	0.000	0.118	0.041	0.055	0.000	0.020	0.287	0.065	0.092	0.000	0.066	0.080	0.015	0.050	0.037	0.020	0.028	0.041	0.020	0.033	0.035	0.000	0.011	0.015	0.019	0.016	0.023	0.029	0.061	0.018	0.000
2,6,8-trimethyl-*trans* -4-nonene	hydrocarbor	11:13.5	00:02.5	**1338**		0.000	0.133	0.005	0.009	0.222	0.011	0.002	0.007	0.007	0.007	0.145	0.014	0.000	0.000	0.030	0.104	0.000	0.000	0.000	0.005	0.026	0.000	0.056	0.000	0.106	0.064	0.000	0.000	0.000	0.003	0.004	0.000
pentadecane	hydrocarbor	14:54.0	00:02.7	**1496**	1500	0.228	0.325	0.178	0.279	0.199	0.323	0.220	0.258	0.229	0.192	0.224	0.179	0.086	0.297	0.155	0.202	0.225	0.111	0.167	0.274	0.424	0.150	0.213	0.187	0.258	0.160	0.232	0.172	0.213	0.171	0.161	0.089
hexadecane	hydrocarbor	17:00.0	00:02.7	**1600**	1600	0.149	0.208	0.155	0.153	0.146	0.205	0.177	0.165	0.153	0.146	0.164	0.099	0.068	0.228	0.123	0.115	0.200	0.085	0.086	0.225	0.302	0.119	0.194	0.123	0.173	0.101	0.144	0.096	0.108	0.120	0.115	0.064
3,3-dimethyl-2-oxobutanal	aldehyde	11:24.8	00:01.9	**1347**		0.000	0.043	0.052	0.302	0.123	0.131	0.128	0.090	0.190	0.079	0.519	0.000	0.013	0.209	0.095	0.208	0.087	0.018	0.272	0.129	0.026	0.000	0.000	0.000	1.406	0.102	0.060	0.012	0.000	0.163	0.000	0.072
nonanal	aldehyde	12:40.6	00:01.5	**1405**	1404	3.847	4.570	8.764	28.210	12.945	10.903	7.424	8.929	15.359	8.882	26.617	7.219	3.979	13.547	9.676	20.709	12.027	4.781	25.309	8.779	4.973	3.714	6.688	2.458	22.407	11.814	4.741	7.015	5.933	18.212	4.651	9.535
trans -2-octenal	aldehyde	13:25.3	00:01.3	**1435**	1432	0.135	0.000	0.588	0.000	0.732	0.475	0.062	0.000	0.000	0.004	0.000	0.059	0.029	0.000	0.027	0.106	0.149	0.144	0.100	0.077	0.083	0.177	0.082	0.085	0.267	0.000	0.166	0.012	0.121	0.000	0.041	0.023
decanal	aldehyde	14:54.0	00:01.5	**1496**	1497	0.053	0.052	0.012	0.038	0.135	0.108	0.093	0.050	0.104	0.103	0.055	0.078	0.070	0.000	0.064	0.075	0.000	0.000	0.113	0.052	0.053	0.062	0.017	0.023	0.000	0.017	0.000	0.015	0.027	0.060	0.067	0.052
undecanal	aldehyde	17:07.0	00:01.5	**1607**	1606	0.047	0.040	0.080	0.057	0.049	0.101	0.066	0.047	0.054	0.085	0.057	0.041	0.036	0.051	0.044	0.062	0.030	0.037	0.037	0.038	0.041	0.034	0.034	0.030	0.060	0.034	0.042	0.045	0.042	0.086	0.026	0.041
trans -2-decenal	aldehyde	17:49.0	00:01.4	**1651**	1647	0.079	0.068	0.147	0.140	0.127	0.114	0.104	0.145	0.118	0.147	0.059	0.124	0.155	0.116	0.200	0.107	0.072	0.130	0.107	0.111	0.078	0.159	0.057	0.063	0.118	0.068	0.021	0.056	0.112	0.153	0.105	0.024
dodecanal	aldehyde	19:06.0	00:01.6	**1727**	1722	0.083	0.053	0.076	0.081	0.042	0.056	0.076	0.062	0.053	0.065	0.053	0.077	0.042	0.095	0.047	0.038	0.049	0.043	0.040	0.063	0.075	0.041	0.057	0.037	0.071	0.041	0.042	0.045	0.098	0.055	0.033	0.042
2-heptanone	ketone	07:46.5	00:05.2	**1180**	1180	0.390	0.644	0.764	0.280	1.249	0.492	1.350	0.371	1.292	0.118	0.341	0.960	0.249	0.603	0.673	0.499	0.343	0.448	1.312	0.893	3.727	0.595	0.094	0.566	0.000	0.442	0.386	0.000	0.615	0.869	0.561	0.075
acetoin	ketone	10:07.9	00:00.8	**1287**	1287	0.054	0.053	0.038	0.050	0.052	0.081	0.028	0.073	0.082	0.171	0.080	0.040	0.031	0.041	0.054	0.114	0.041	0.054	0.043	0.049	0.078	0.049	0.068	0.074	0.099	0.275	0.044	0.042	0.050	0.074	0.069	0.031
5-methyl-5-hepten-2-one	ketone	11:24.0	00:01.3	**1346**	1343	0.147	0.073	0.467	0.000	0.114	0.007	0.069	0.296	0.150	0.000	0.115	0.143	0.230	0.153	0.000	0.153	0.103	0.132	0.145	0.094	0.108	0.137	0.056	0.069	0.366	0.087	0.062	0.056	0.115	0.098	0.092	0.320
2-nonanone	ketone	12:34.0	00:01.4	**1401**	1402	6.242	4.236	16.613	2.527	17.136	5.011	27.871	1.662	8.915	10.522	2.231	3.401	0.742	2.015	3.320	2.159	2.266	2.524	5.039	5.669	22.244	5.425	3.641	2.531	2.751	2.132	4.643	2.072	4.598	4.379	1.965	0.330
3-(acetoxy)-4-methyl-2-pentanone	ketone	14:07.3	00:01.3	**1464**		0.000	0.022	0.058	0.000	0.000	0.000	0.017	0.000	0.077	0.070	0.000	0.000	0.040	0.040	0.158	0.157	0.032	0.115	0.061	0.031	0.000	0.114	0.000	0.058	0.009	0.027	0.025	0.000	0.059	0.000	0.047	0.045
trans -5-methyl-2-(1-methylethyl)-cy	ketone	14:08.1	00:01.6	**1464**	1473	0.031	0.100	0.000	0.000	0.114	0.000	0.043	0.041	0.161	0.055	0.059	0.050	1.798	0.060	0.000	0.072	0.068	0.028	0.118	0.000	0.138	0.114	0.092	0.081	0.013	0.032	0.071	0.040	0.080	0.032	0.108	3.361
unsaturated diketone (n.i.)	ketone	14:26.0	00:01.1	**1477**		0.156	0.000	0.362	0.023	0.362	0.145	0.022	0.028	0.267	0.408	0.289	0.572	0.457	0.228	0.308	0.350	0.189	0.431	0.253	0.195	0.178	0.288	0.021	0.185	0.000	0.144	0.154	0.154	0.530	0.180	0.024	0.245
2-decanone	ketone	14:47.0	00:01.5	**1491**	1491	0.432	0.000	0.338	0.900	0.387	0.732	0.824	0.406	0.500	0.566	0.327	0.393	0.435	0.315	0.663	0.434	0.336	0.475	0.465	0.000	0.416	0.513	0.332	0.325	0.692	0.303	0.588	0.295	0.491	0.326	0.307	0.132
diketone (n.i.)	ketone	15:07.1	00:01.2	**1505**		0.048	0.000	0.111	0.135	0.115	0.101	0.179	0.095	0.099	0.087	0.130	0.203	0.050	0.047	0.112	0.114	0.104	0.042	0.168	0.000	0.036	0.095	0.144	0.191	0.240	0.155	0.104	0.152	0.098	0.110	0.055	0.124
3-undecanone	ketone	16:20.3	00:01.6	**1567**	1571	0.055	0.250	0.060	0.905	0.215	1.055	0.973	0.128	0.004	0.070	0.046	0.043	0.092	0.163	0.104	0.103	0.053	0.042	0.069	0.055	0.089	0.159	0.043	0.034	0.721	0.001	0.059	0.003	0.003	0.037	0.037	0.000
isophorone	ketone	16:46.0	00:01.3	**1588**	1593	0.092	0.090	0.138	0.103	0.109	0.110	0.149	0.137	0.085	0.062	0.059	0.133	0.116	0.116	0.133	0.059	0.025	0.056	0.089	0.030	0.020	0.052	0.081	0.109	0.076	0.108	0.065	0.086	0.122	0.077	0.076	0.127
2-undecanone	ketone	16:53.4	00:01.5	**1594**	1598	0.271	0.510	1.132	1.057	0.428	1.274	1.250	0.121	0.384	0.447	0.240	0.232	0.193	0.355	0.387	0.341	0.276	0.249	0.290	0.274	0.142	0.484	0.239	0.241	0.891	0.224	0.278	0.191	0.362	0.337	0.276	0.030
cyclohexylideneacetone	ketone	17:35.0	00:01.8	**1637**		0.047	0.036	0.307	0.065	0.067	0.032	0.046	0.179	0.307	0.573	0.186	0.605	1.507	0.772	0.746	0.429	0.223	0.626	0.331	0.304	0.154	0.899	0.293	0.628	0.282	0.837	0.356	0.523	0.902	0.828	1.991	0.764
4-(1,1-dimethylethyl)-cyclohexanone	ketone	17:35.0	00:01.5	**1637**	1645	0.076	0.107	0.269	0.007	0.000	0.000	0.000	0.000	0.138	0.017	0.197	0.062	0.361	0.039	0.055	0.082	0.005	0.006	0.063	0.000	0.020	0.000	0.000	0.000	0.000	0.010	0.024	0.015	0.026	0.236	0.036	0.006
acetophenone	ketone	17:56.0	00:01.1	**1659**	1660	0.249	0.236	0.264	0.345	0.364	0.346	0.283	0.289	0.721	0.661	0.428	0.406	0.516	0.581	0.589	0.487	0.269	0.500	0.484	0.294	0.350	0.520	0.196	0.292	0.226	0.301	0.210	0.248	0.424	0.295	0.277	0.889
3,4-dihydroxy-cyclobutene-1,2-dione	ketone	18:10.0	00:01.1	**1673**		0.042	0.064	0.112	0.100	0.133	0.064	0.088	0.112	0.068	0.127	0.031	0.085	0.119	0.042	0.056	0.090	0.076	0.162	0.031	0.018	0.015	0.032	0.037	0.076	0.041	0.070	0.035	0.086	0.203	0.061	0.060	0.098
2,2-dimethyl-1,3-dioxane-4,6-dione	ketone	18:17.0	00:01.1	**1681**		0.033	0.000	0.030	0.039	0.040	0.040	0.041	0.038	0.000	0.038	0.032	0.000	0.000	0.034	0.050	0.044	0.025	0.040	0.048	0.029	0.027	0.046	0.033	0.037	0.028	0.037	0.029	0.035	0.047	0.035	0.044	0.000
1b,5,5,6a-tetramethyl-octahydro-1-o:	ketone	18:31.0	00:02.1	**1695**		0.000	0.000	0.000	0.017	0.000	0.051	0.030	0.029	0.021	0.036	0.019	0.042	0.000	0.058	0.116	0.092	0.020	0.089	0.000	0.000	0.000	0.126	0.041	0.085	0.016	0.202	0.043	0.074	0.158	0.098	0.272	0.000
4,4-(ethylenedioxy)-2-pentanone	ketone	19:20.0	00:01.1	**1739**		0.141	0.148	0.080	0.216	0.115	0.203	0.224	0.200	0.245	0.339	0.273	0.109	0.373	0.080	0.293	0.040	0.256	0.138	0.129	0.091	0.085	0.277	0.099	0.122	0.133	0.077	0.089	0.104	0.092	0.179	0.058	0.069
1-phenyl-1-propanone	ketone	19:20.2	00:01.2	**1739**	1744	0.020	0.000	0.000	0.000	0.031	0.030	0.050	0.020	0.056	0.033	0.017	0.023	0.030	0.022	0.026	0.000	0.013	0.020	0.020	0.017	0.015	0.022	0.013	0.015	0.018	0.016	0.011	0.013	0.022	0.018	0.017	0.027
2-cyclohexene-1,4-dione	ketone	19:33.7	00:01.0	**1751**		0.000	0.028	0.000	0.009	0.000	0.000	0.000	0.015	0.019	0.013	0.000	0.094	0.017	0.025	0.022	0.000	0.026	0.010	0.012	0.577	0.009	0.017	0.012	0.000	0.028	0.006	0.165	0.008	0.000	0.012	0.009	0.146
3-tridecanone	ketone	20:16.0	00:01.7	**1787**	1755	0.008	0.040	0.012	0.061	0.021	0.067	0.056	0.023	0.008	0.011	0.000	0.000	0.012	0.022	0.015	0.000	0.010	0.000	0.010	0.000	0.016	0.023	0.010	0.000	0.077	0.006	0.014	0.007	0.012	0.009	0.010	0.000
2H-pyran-2,6(3H)-dione	ketone	24:10.5	00:00.8	**1995**		0.325	0.531	0.204	0.357	0.556	0.829	0.534	0.547	0.980	0.674	0.356	0.316	0.957	0.327	1.015	0.372	0.844	0.648	0.847	0.361	0.314	0.560	0.303	0.635	0.441	0.502	0.278	0.252	0.368	0.318	0.414	0.451
2,6-di(*tert* -butyl)-4-hydroxy-4-methy	ketone	25:45.0	00:01.1	**2077**	2094	0.012	0.005	0.011	0.007	0.013	0.006	0.007	0.007	0.009	0.015	0.004	0.006	0.020	0.006	0.014	0.005	0.003	0.024	0.010	0.009	0.008	0.011	0.000	0.013	0.009	0.014	0.018	0.035	0.011	0.010	0.007	0.022
1,4,7,10,13-pentaoxacyclononadecan	ketone	27:35.0	00:01.3	**>2100**		0.008	0.005	0.008	0.109	0.006	0.009	0.007	0.060	0.008	0.011	0.004	0.012	0.042	0.010	0.000	0.005	0.007	0.000	0.001	0.008	0.005	0.008	0.009	0.062	0.008	0.009	0.014	0.037	0.014	0.104	0.070	0.146
1-pentanol	alcohol	08:53.5	00:00.5	**1231**	1242	0.000	0.062	0.052	0.000	0.260	0.090	0.173	0.021	0.145	0.138	0.320	0.114	0.059	0.000	0.169	0.078	0.010	0.141	0.099	0.057	0.241	0.159	0.144	0.000	0.000	0.059	0.095	0.000	0.108	0.035	0.055	0.029
2-methyl-1-pentanol	alcohol	10:28.5	00:00.9	**1303**	1297	0.166	0.137	0.184	0.000	1.042	0.147	0.119	0.304	0.000	0.000	0.527	0.000	0.562	0.094	0.220	0.085	1.239	0.506	0.000	0.136	0.117	0.177	0.155	0.501	0.111	0.331	0.422	0.410	0.256	0.336	0.093	0.169
4-methyl-1-pentanol	alcohol	10:49.0	00:00.9	**1319**	1319	1.881	1.872	1.471	1.904	4.023	2.292	3.878	1.057	3.555	2.079	2.493	1.165	2.905	3.188	2.994	3.998	1.132	6.699	4.793	1.420	3.197	1.985	1.120	1.222	0.902	2.406	1.102	1.394	6.762	1.912	1.653	1.780
2-heptanol	alcohol	10:56.0	00:01.0	**1324**	1320	0.855	0.631	1.584	0.886	0.662	0.783	0.857	1.288	2.079	2.520	1.243	3.107	1.441	0.609	2.892	0.563	0.740	1.443	0.902	1.274	1.290	1.099	0.456	0.719	0.338	0.503	0.508	0.579	1.105	1.218	1.379	2.117
2-penten-1-ol	alcohol	10:56.2	00:00.8	**1324**	1321	0.037	0.029	0.078	0.018	0.069	0.056	0.031	0.034	0.053	0.068	0.036	0.037	0.053	0.024	0.041	0.031	0.016	0.044	0.069	0.047	0.041	0.057	0.014	0.010	0.012	0.011	0.011	0.012	0.017	0.031	0.035	0.054
8-methyl-1,8-nonanediol	alcohol	10:56.2	00:01.1	**1324**		0.155	0.160	0.213	0.000	0.143	0.152	0.000	0.163	0.187	0.363	0.165	0.246	0.139	0.109	0.186	0.256	0.000	0.245	0.335	0.250	0.129	0.347	0.170	0.234	0.171	0.201	0.062	0.285	0.317	0.182	0.216	0.000
3-methyl-1-pentanol	alcohol	11:03.7	00:00.9	**1330**	1332	4.503	5.993	3.019	6.402	6.976	6.072	7.493	5.863	6.484	4.759	4.990	7.425	7.346	3.325	6.264	8.036	5.525	5.501	11.776	2.221	5.332	6.433	2.470	2.333	2.934	4.501	2.435	3.007	7.345	4.626	4.230	4.502
cis -3-hexen-1-ol	alcohol	12:20.0	00:00.9	**1390**	1386	5.032	6.076	10.349	4.144	10.054	10.368	6.253	5.256	14.988	13.396	11.654	20.580	18.226	13.661	19.411	8.168	6.589	14.449	20.302	9.350	7.393	12.264	3.436	2.809	3.175	2.793	3.494	3.375	4.598	3.845	3.917	9.420
3-octanol	alcohol	12:36.4	00:01.1	**1402**	1406	0.171	0.000	0.141	0.027	0.039	0.069	0.043	0.168	0.217	0.120	0.141	0.221	0.275	0.084	0.237	0.026	0.026	0.124	0.133	0.000	0.034	0.091	0.044	0.018	0.064	0.029	0.072	0.125	0.151	0.000	0.054	0.107
4-methyl-1-heptanol	alcohol	12:42.1	00:01.6	**1406**	1409	0.385	0.297	0.112	0.204	0.623	0.211	0.242	0.425	0.162	0.000	0.083	0.125	0.150	0.108	0.174	0.135	0.010	0.000	0.087	0.039	0.042	0.076	0.016	0.011	0.027	0.040	0.015	0.024	0.099	0.623	0.854	0.436
2-octanol	alcohol	13:09.0	00:01.0	**1424**	1418	40.000	40.000	40.000	40.000	40.000	40.000	40.000	40.000	40.000	40.000	40.000	40.000	40.000	40.000	40.000	40.000	40.000	40.000	40.000	40.000	40.000	40.000	40.000	40.000	40.000	40.000	40.000	40.000	40.000	40.000	40.000	40.000
1-octen-3-ol	alcohol	13:51.0	00:01.0	**1453**	1452	5.125	1.342	4.797	2.009	1.349	2.234	1.560	9.535	7.066	3.960	6.705	4.921	5.102	4.306	8.519	2.234	2.076	3.635	2.679	1.785	2.922	1.936	1.791	1.315	2.324	2.150	2.425	2.987	5.184	3.643	1.575	3.829
1-heptanol	alcohol	13:58.2	00:00.9	**1458**	1457	1.989	0.456	2.087	0.829	0.695	0.894	0.753	2.199	1.260	1.920	1.437	1.184	2.516	0.518	0.926	0.977	0.712	1.088	1.019	0.017	2.987	1.039	0.740	0.453	0.604	0.894	0.839	0.947	1.645	1.284	0.802	1.462
6-methyl-5-hepten-2-ol	alcohol	14:05.8	00:01.0	**1463**	1466	0.036	0.029	0.029	0.036	0.031	0.034	0.032	0.046	0.056	0.061	0.046	0.036	0.047	0.030	0.075	0.033	0.041	0.053	0.038	0.035	0.028	0.065	0.029	0.042	0.030	0.042	0.032	0.029	0.059	0.026	0.021	0.044
alcohol (n.i.)	alcohol	14:17.6	00:01.0	**1471**		0.056	0.339	0.057	0.261	0.000	0.222	0.078	0.083	0.029	0.000	0.000	0.000	0.001	0.055	0.000	0.000	0.134	0.000	0.000	0.273	0.218	0.050	0.139	0.066	0.159	0.086	0.062	0.092	0.043	0.065	0.090	0.006
2-ethylhexanol	alcohol	14:40.0	00:01.0	**1486**	1484	5.304	3.848	4.217	4.017	6.009	4.643	4.160	4.357	4.408	4.392	4.318	4.770	5.435	4.004	5.318	5.228	4.085	4.618	5.373	4.784	4.436	5.255	3.915	4.056	4.613	3.826	3.841	3.908	5.024	4.045	3.971	4.351
4-hepten-1-ol	alcohol	14:54.5	00:00.9	**1496**	1502	0.149	0.065	0.225	0.115	0.099	0.110	0.081	0.000	0.000	0.000	0.172	0.170	0.000	0.114	0.196	0.139	0.117	0.145	0.164	0.129	0.000	0.184	0.000	0.028	0.056	0.048	0.037	0.060	0.076	0.159	0.123	0.235
alcohol (n.i.)	alcohol	15:02.3	00:01.0	**1501**		0.244	0.158	0.410	0.312	0.146	0.113	0.145	2.400	0.672	1.211	0.579	0.599	1.469	0.388	1.022	0.422	0.411	2.069	1.101	1.411	1.807	1.026	0.328	0.195	0.174	0.052	0.183	0.196	0.215	0.054	0.062	0.120
4-ethyl-3-octanol	alcohol	15:08.0	00:01.2	**1506**		0.433	0.302	0.413	0.387	0.407	0.328	0.467	0.461	0.392	0.413	0.357	0.429	0.302	0.273	0.451	0.508	0.324	0.608	0.634	0.000	0.316	0.618	0.354	0.440	0.390	0.445	0.000	0.412	0.681	0.351	0.383	0.000
2-nonanol	alcohol	15:15.0	00:01.1	**1512**	1518	0.403	0.413	0.767	0.340	0.825	0.422	0.557	0.367	0.906	1.196	0.361	0.371	0.441	0.243	0.551	0.299	0.288	0.465	0.539	0.324	0.480	0.611	0.240	0.653	0.272	0.375	0.305	0.319	0.622	0.462	0.743	0.199
2,3-butanediol I	alcohol	15:36.0	00:02.1	**1530**	1542	3.247	5.718	2.172	3.346	5.408	3.543	3.688	0.066	4.694	4.093	4.483	3.749	2.828	2.511	8.017	4.180	8.130	0.735	4.317	3.558	2.260	4.340	1.731	3.438	1.473	2.042	2.188	1.294	0.043	2.772	4.514	3.505
2,3-butanediol II	alcohol	16:25.2	00:00.8	**1571**	1567	1.725	2.865	1.366	1.731	1.737	2.242	2.046	1.999	3.078	2.214	2.918	2.555	3.153	1.506	2.783	5.019	3.549	1.335	1.890	2.260	1.761	2.286	1.399	3.049	1.311	1.368	0.771	0.673	1.376	1.831	1.999	2.190
2-octen-1-ol	alcohol	17:14.0	00:01.0	**1615**	1622	0.088	0.079	0.155	0.061	0.047	0.058	0.061	0.655	0.119	0.143	0.088	0.090	0.231	0.046	0.080	0.080	0.043	0.103	0.135	0.067	0.045	0.069	0.025	0.018	0.045	0.048	0.037	0.064	0.049	0.069	0.034	0.067
2-decanol	alcohol	17:20.3	00:01.1	**1621**	1621	0.015	0.019	0.024	0.000	0.030	0.032	0.034	0.027	0.077	0.028	0.053	0.010	0.078	0.033	0.053	0.011	0.016	0.043	0.037	0.019	0.018	0.000	0.034	0.032	0.016	0.024	0.001	0.025	0.039	0.579	0.025	0.032
1-nonanol	alcohol	18:03.3	00:01.0	**1666**	1661	0.937	0.981	5.297	0.650	1.609	0.000	1.228	0.662	0.035	0.990	1.327	0.894	0.999	1.188	0.000	0.869	0.000	0.830	10.498	0.667	1.329	3.413	0.913	0.502	0.000	6.567	0.508	0.721	1.688	0.687	2.367	0.000
3-ethyl-4-octanol	alcohol	18:24.9	00:01.3	**1689**		0.075	0.446	0.000	0.531	0.390	0.462	0.459	0.548	0.034	0.396	0.460	0.058	0.279	0.397	0.504	0.055	0.433	0.585	0.634	0.570	0.509	0.567	0.520	0.574	0.325	0.424	0.385	0.444	0.628	0.468	0.458	0.066
2-undecanol	alcohol	19:13.0	00:01.2	**1733**	1738	0.000	0.161	0.661	0.152	0.000	0.000	0.248	0.159	0.539	0.557	0.320	0.147	0.204	0.109	0.434	0.000	0.110	0.206	0.301	0.323	0.232	0.408	0.161	0.301	0.189	0.161	0.282	0.184	0.412	0.317	0.460	0.056

3,5-dimethyl-4-heptanol	alcohol	19:35.2	00:00.8	**1752**		0.051	0.067	0.000	0.000	0.089	0.043	0.056	0.035	0.161	0.078	0.123	0.025	0.120	0.028	0.189	0.116	0.098	0.191	0.117	0.062	0.000	0.100	0.000	0.089	0.021	0.078	0.014	0.016	0.154	0.069	0.070	0.066
trans-4-tert-butylcyclohexanol	alcohol	19:43.3	00:01.1	**1759**	1730	0.940	0.029	0.089	0.008	0.004	0.008	0.005	0.006	0.000	0.002	0.191	0.030	0.048	0.000	0.773	0.881	0.000	0.003	0.958	0.003	0.017	0.004	0.058	0.251	0.087	0.222	0.562	0.218	0.030	0.051	0.598	0.000
3,4-nonadienol	alcohol	19:48.0	00:01.0	**1763**	1754	0.017	0.006	0.015	0.009	0.000	0.006	0.008	0.078	0.011	0.013	0.007	0.015	0.044	0.006	0.018	0.004	0.000	0.008	0.016	0.007	0.007	0.008	0.002	0.000	0.003	0.003	0.000	0.005	0.006	0.006	0.004	0.006
1-decanol	alcohol	20:02.0	00:01.1	**1775**	1778	0.631	0.436	1.176	0.742	0.690	0.554	0.653	0.801	0.772	0.617	0.532	0.516	0.673	0.545	0.721	0.518	0.358	0.607	0.610	0.815	0.618	0.652	0.859	0.817	0.757	0.892	0.630	0.923	0.803	0.870	0.595	0.459
1-undecanol	alcohol	21:49.4	00:01.1	**1871**	1883	0.000	0.010	0.000	0.003	0.002	0.001	0.001	0.015	0.034	0.024	0.032	0.002	0.003	0.020	0.042	0.000	0.000	0.001	0.031	0.000	0.011	0.003	0.012	0.017	0.017	0.025	0.018	0.026	0.043	0.017	0.030	0.018
2-phenylethanol	alcohol	22:44.4	00:00.9	**1920**	1921	0.008	0.142	0.009	0.029	0.076	0.040	0.015	0.006	0.000	0.001	0.000	0.000	0.000	0.007	0.000	0.053	1.212	0.000	0.000	0.004	0.019	0.003	0.003	0.002	0.004	0.000	0.000	0.000	0.000	0.000	0.000	0.001
acid (n.i.)	acid	14:33.0	00:01.1	**1481**	1491	0.045	0.013	0.000	0.032	0.014	0.017	0.019	0.000	0.038	0.061	0.037	0.014	0.024	0.022	0.068	0.023	0.019	0.019	0.038	0.000	0.000	0.013	0.009	0.000	0.010	0.009	0.009	0.010	0.019	0.008	0.008	0.022
formic acid	acid	15:10.4	00:00.7	**1508**	1501	1.237	1.050	1.296	1.148	1.444	1.639	2.500	1.218	1.876	1.491	1.262	3.252	1.791	1.627	3.346	1.743	1.158	1.720	1.838	1.390	0.875	1.935	0.732	1.085	0.807	1.541	0.953	0.950	2.160	0.915	1.114	1.889
propionic acid	acid	15:43.0	00:00.7	**1536**	1540	1.097	1.191	0.877	1.125	1.388	1.631	1.879	1.165	2.380	1.655	1.545	1.254	1.824	0.898	1.862	1.536	1.320	1.033	1.201	0.860	0.739	1.421	0.799	1.173	0.875	1.133	0.817	0.820	1.005	0.880	0.713	1.381
2-decenoic acid	acid	15:43.7	00:00.8	**1536**	1540	0.044	0.012	0.013	0.063	0.000	0.020	0.014	0.000	0.062	0.000	0.000	0.024	0.000	0.000	0.000	0.000	0.000	0.000	0.000	0.023	0.015	0.000	0.022	0.015	0.016	0.030	0.017	0.040	0.038	0.000	0.016	0.049
isobutyric acid	acid	16:18.0	00:00.7	**1565**	1555	3.167	3.346	3.766	2.761	4.881	3.534	3.874	1.446	6.555	7.239	4.042	3.404	4.034	3.671	4.129	4.574	2.718	8.614	2.898	4.075	4.166	2.438	3.341	2.840	2.209	4.289	2.812	3.588	7.489	1.692	2.055	5.854
butyric acid	acid	17:28.0	00:00.7	**1629**	1626	18.897	16.745	14.080	17.782	16.598	19.127	19.825	22.872	18.512	19.619	19.311	11.386	28.167	16.326	21.812	16.737	17.691	13.031	17.944	17.895	20.289	19.766	16.037	18.242	16.792	16.182	15.717	16.087	17.411	15.906	13.119	25.407
2-propenoic acid	acid	17:42.7	00:00.7	**1645**		0.249	0.249	0.249	0.079	0.260	0.308	0.320	0.245	0.233	0.286	0.302	0.350	0.315	0.300	0.046	0.322	0.282	0.264	0.259	0.278	0.272	0.183	0.203	0.229	0.163	0.207	0.197	0.212	0.289	0.194	0.158	0.086
isovaleric acid	acid	18:18.8	00:00.7	**1683**	1680	5.523	6.574	4.721	4.271	7.242	5.961	8.284	4.090	9.981	0.184	0.000	4.520	0.174	6.567	6.056	6.974	5.373	9.418	7.212	0.264	6.853	5.371	5.003	4.626	4.702	7.833	5.053	5.224	10.467	3.519	3.472	8.674
pentanoic acid	acid	19:34.0	00:00.8	**1751**	1751	0.405	0.347	0.346	0.453	0.419	0.300	0.492	0.506	0.541	0.598	0.356	0.343	0.744	0.388	0.460	0.344	0.328	0.474	0.510	0.323	0.370	0.419	0.375	0.368	0.401	0.401	0.314	0.376	0.522	0.317	0.320	0.904
trans-3-hexenoic acid	acid	22:49.8	00:00.8	**1924**	1929	0.004	0.005	0.059	0.075	0.079	0.007	0.013	0.010	0.000	0.010	0.000	0.010	0.012	0.003	0.010	0.012	0.004	0.004	0.008	0.009	0.003	0.006	0.003	0.004	0.005	0.004	0.000	0.010	0.008	0.017	0.008	0.008
2-ethylhexanoic acid	acid	23:18.0	00:00.8	**1949**	1960	0.000	0.000	0.000	0.000	0.306	0.903	0.000	3.611	0.124	0.669	0.095	0.000	0.372	0.158	0.207	0.205	0.193	0.137	0.115	0.844	0.171	0.519	0.104	0.087	0.083	0.145	0.137	0.227	0.226	0.169	0.152	0.132
heptanoic acid	acid	23:22.2	00:00.8	**1953**	1955	0.032	0.099	0.060	0.060	0.061	0.075	0.092	0.092	0.059	0.155	0.053	0.024	0.112	0.052	0.072	0.034	0.089	0.042	0.072	0.057	0.040	0.249	0.075	0.043	0.093	0.051	0.044	0.041	0.074	0.308	0.040	0.108
trans-2-hexenoic acid	acid	23:39.0	00:00.8	**1967**	1967	0.024	0.094	0.154	0.051	0.136	0.093	0.058	0.037	0.320	0.179	0.161	0.060	0.270	0.147	0.295	0.117	0.043	0.091	0.370	0.120	0.210	0.164	0.091	0.170	0.049	0.084	0.053	0.035	0.101	0.110	0.178	0.509
3,5,5-trimethylhexanoic acid	acid	23:46.0	00:00.8	**1973**		0.314	0.256	0.396	0.333	0.293	0.282	0.372	0.395	0.368	0.345	0.304	0.192	0.568	0.285	0.364	0.348	0.231	0.437	0.502	0.310	0.254	0.504	0.350	0.434	0.489	0.443	0.324	0.414	0.608	0.416	0.426	0.595
nonanoic acid	acid	26:51.5	00:00.8	**>2100**	2119	0.069	0.106	0.138	0.142	0.000	0.088	0.077	0.144	0.128	0.252	0.095	0.066	0.330	0.197	0.118	0.080	0.072	0.102	0.189	0.078	0.066	0.073	0.079	0.093	0.478	0.093	0.074	0.068	0.279	0.362	0.354	0.223
isobutyl acetate	ester	04:23.8	00:04.7	**1015**	1009	0.824	0.495	0.562	0.912	0.542	1.510	0.295	0.665	0.879	1.257	0.575	0.381	0.832	0.858	0.632	0.282	1.456	1.029	0.208	0.612	0.879	0.626	0.699	0.761	0.562	0.465	0.511	0.519	0.220	0.851	0.193	0.932
ethyl butyrate	ester	04:45.7	00:04.9	**1034**	1033	2.326	1.652	1.498	2.244	1.441	2.369	2.539	1.722	1.669	1.757	2.586	1.912	0.148	1.935	2.525	1.602	1.860	1.690	2.247	2.496	1.853	3.005	2.743	1.582	1.575	2.051	1.560	1.577	1.718	2.022	1.233	1.868
ethyl 2-methylbutyrate	ester	05:05.8	00:01.4	**1052**	1050	0.229	0.380	0.735	0.263	0.472	0.251	0.101	0.350	0.888	0.700	0.452	0.221	0.586	1.192	0.738	0.295	0.093	2.824	1.129	0.710	0.098	1.100	1.249	0.861	0.529	2.071	1.166	0.436	3.537	0.045	0.329	0.359
ethyl 3-methylbutyrate	ester	05:19.8	00:05.1	**1064**	1068	0.546	1.437	0.595	0.570	0.558	0.631	1.369	0.578	2.031	1.757	2.627	0.933	0.000	3.099	2.638	3.154	1.414	1.490	0.826	0.473	0.516	1.954	0.570	0.658	0.429	1.126	0.754	0.591	3.427	0.445	0.628	0.000
ethyl *trans*-2-butenoate	ester	07:19.0	00:05.0	**1159**	1161	9.402	8.341	5.316	16.933	1.940	9.370	6.484	10.620	6.340	2.700	0.687	3.768	5.163	7.519	10.252	4.970	12.157	8.958	4.320	8.775	5.012	8.051	0.000	7.291	2.507	4.880	5.595	7.403	2.957	6.568	5.412	0.899
pentyl acetate	ester	07:36.1	00:01.3	**1172**	1161	0.471	0.000	0.105	0.000	0.000	0.000	0.404	0.000	0.000	0.546	0.000	0.000	0.000	0.068	0.000	0.023	0.000	0.000	0.000	0.119	0.000	0.117	0.419	0.152	0.500	0.284	0.000	0.403	0.219	0.113	0.046	0.000
methyl hexanoate	ester	07:51.1	00:05.4	**1183**	1188	7.490	3.956	6.616	10.433	5.019	3.099	5.129	8.180	2.362	3.637	3.069	1.702	0.408	1.535	4.206	1.557	3.408	2.283	2.607	2.836	3.264	5.053	2.856	2.560	2.239	2.022	2.514	1.814	1.507	2.311	1.222	0.133
isoamyl propanoate	ester	07:56.8	00:01.5	**1188**	1188	0.741	0.296	0.467	0.606	0.938	0.594	1.062	0.399	1.333	0.813	0.624	0.826	0.026	0.233	0.883	1.500	0.357	0.867	1.167	0.210	0.259	0.822	0.236	0.716	0.302	0.922	0.241	0.348	1.033	0.306	0.402	0.043
3-methyl-3-buten-1-yl acetate	ester	08:07.8	00:01.2	**1196**	1190	0.047	0.010	0.045	0.114	0.075	0.074	0.100	0.112	0.052	0.092	0.083	0.128	0.031	0.028	0.047	0.000	0.081	0.036	0.042	0.022	0.000	0.045	0.061	0.076	0.060	0.024	0.035	0.062	0.023	0.053	0.023	0.000
trans-penten-1-yl acetate	ester	08:50.0	00:01.2	**1228**		0.033	0.038	0.126	0.028	0.120	0.000	0.051	0.000	0.082	0.123	0.000	0.000	0.016	0.000	0.055	0.000	0.000	0.000	0.069	0.023	0.008	0.066	0.022	0.022	0.019	0.000	0.017	0.000	0.000	0.046	0.025	0.000
ethyl 2-propynoate	ester	09:11.0	00:01.8	**1244**		4.425	4.277	6.420	4.922	3.385	2.984	5.041	4.949	5.877	4.221	4.136	3.587	1.967	4.175	6.305	4.372	4.837	5.317	5.139	2.249	4.187	7.276	3.444	4.188	3.798	5.944	3.879	2.183	6.818	5.081	4.897	0.706
isoamyl butyrate	ester	09:45.8	00:01.7	**1270**	1266	4.282	2.426	2.410	2.996	2.072	1.030	2.456	2.912	2.500	2.719	2.481	2.585	0.000	2.130	2.504	1.771	1.876	1.370	1.230	3.660	2.898	1.581	1.494	1.930	2.178	1.712	1.966	2.522	1.582	2.311	0.956	0.000
ethyl pyruvate	ester	09:53.4	00:01.0	**1276**	1276	0.809	2.030	1.779	0.715	2.580	2.344	2.097	1.335	2.438	4.036	2.841	4.568	2.164	2.533	2.596	3.387	1.182	3.296	1.361	1.179	0.695	2.421	1.597	1.044	1.349	1.393	1.403	0.836	2.853	1.761	1.231	2.064
ethyl 4-hexenoate	ester	10:21.3	00:01.4	**1297**	1292	1.139	1.229	14.070	28.485	13.751	0.092	2.638	0.235	2.893	4.164	0.590	0.513	1.241	1.947	2.961	1.850	1.421	1.973	0.000	1.355	1.038	1.369	0.239	0.352	0.699	1.515	0.880	1.216	0.585	2.938	0.747	0.405
trans-3-hexen-1-yl acetate	ester	10:42.0	00:01.3	**1313**	1316	9.122	14.766	20.276	31.281	15.079	23.053	27.983	21.013	11.091	24.591	17.065	5.659	2.159	3.062	6.883	2.249	5.017	5.737	11.858	8.043	3.556	4.992	14.074	10.971	15.906	8.451	8.497	12.636	3.177	16.753	5.061	0.352
propyl hexanoate	ester	10:56.2	00:01.7	**1324**	1319	0.484	0.428	0.804	1.489	0.576	0.821	0.783	0.693	0.148	0.530	0.351	0.884	0.067	0.138	0.256	0.337	0.718	0.025	0.086	0.250	0.216	0.369	0.433	0.368	0.552	0.354	0.517	0.306	0.125	0.359	0.152	0.000
cis-3-hexen-1-yl acetate	ester	10:56.0	00:01.3	**1324**	1300	7.975	15.477	25.524	17.095	27.998	35.475	12.832	17.953	34.723	45.504	40.017	55.836	29.187	23.632	46.473	3.648	24.763	25.927	47.892	20.732	8.373	28.900	7.656	7.290	10.471	4.380	6.725	6.061	2.022	7.759	4.872	1.096
ethyl heptanoate	ester	11:17.0	00:01.6	**1341**	1342	5.437	2.363	2.711	3.897	2.875	2.105	2.228	2.437	1.274	2.114	1.327	4.386	0.308	0.698	1.026	2.468	2.471	0.787	0.967	1.654	2.252	1.307	1.448	0.872	1.907	1.509	1.728	1.244	1.776	2.198	0.583	0.033
ethyl lactate	ester	11:24.0	00:01.7	**1346**	1349	19.326	11.704	16.666	17.457	75.559	10.182	22.308	32.756	13.430	180.992	15.754	21.819	40.434	21.889	19.625	40.601	151.720	27.948	20.712	86.809	6.623	58.869	13.145	23.141	13.128	25.330	25.029	31.284	82.470	26.410	22.631	79.158
ethyl 2-hexenoate	ester	11:31.7	00:01.5	**1352**	1357	0.739	1.763	3.210	0.966	2.779	1.838	1.347	0.369	2.437	2.607	2.216	1.003	0.598	2.120	2.068	1.862	0.984	1.426	4.737	1.875	1.845	2.020	1.937	2.856	0.817	1.561	0.816	0.514	1.357	1.583	3.327	0.252
methyl 2-hydroxybutanoate	ester	11:45.0	00:01.1	**1362**	1382	0.329	0.222	0.386	0.260	0.246	0.276	0.248	0.226	0.000	0.362	0.000	0.225	0.344	0.302	0.286	0.344	0.175	0.364	0.331	0.000	0.241	0.000	0.251	0.294	0.265	0.231	0.266	0.297	0.354	0.364	0.312	0.000
isobutyl hexanoate	ester	11:45.0	00:01.8	**1362**	1357	1.640	0.635	2.023	0.834	1.077	1.185	0.569	0.351	0.267	0.663	0.316	0.903	0.000	0.316	0.278	0.718	0.556	0.501	0.128	1.624	1.517	0.328	0.438	0.414	0.456	0.688	0.643	0.538	0.986	0.543	0.235	0.000
3-ethoxypropyl acetate	ester	11:52.0	00:01.2	**1368**		0.405	0.636	0.050	0.961	0.293	1.282	0.827	1.576	1.133	3.705	3.170	0.693	3.377	0.216	2.937	0.154	2.467	0.122	0.265	0.056	0.000	1.403	0.189	0.826	0.636	0.179	0.958	0.307	0.000	1.268	0.000	0.093
heptyl acetate	ester	12:13.4	00:01.5	**1385**	1385	0.484	0.136	0.184	0.171	0.184	0.216	0.113	0.402	0.430	0.242	0.511	0.117	0.028	0.105	0.307	0.021	0.191	0.156	0.163	0.094	0.121	0.313	0.082	0.091	0.132	0.097	0.142	0.206	0.089	0.290	0.139	0.000
hexanodibutyrin	ester	12:20.0	00:01.7	**1390**		0.543	0.652	0.157	0.505	0.644	0.366	0.453	0.236	0.516	0.599	0.496	0.479	0.178	0.952	0.778	0.514	0.161	0.159	0.217	0.000	0.000	0.205	0.089	0.128	0.150	0.218	0.116	0.158	0.393	0.597	1.082	0.436
3-methylheptyl acetate	ester	12:26.5	00:01.6	**1395**		0.175	0.197	0.176	0.217	1.145	0.598	0.131	0.298	0.616	0.272	0.131	0.000	0.049	0.177	0.406	0.268	0.208	0.166	0.000	0.193	0.209	0.230	0.000	0.120	0.137	0.125	0.122	0.144	0.134	0.107	0.208	0.056
methyl octanoate	ester	12:34.0	00:01.5	**1401**	1404	65.869	50.267	47.985	63.653	36.377	38.292	34.685	72.809	10.325	21.680	14.824	29.633	4.213	10.125	11.312	17.300	36.526	15.886	6.778	36.191	44.502	20.258	27.000	20.292	22.575	20.389	25.799	20.052	13.739	27.907	7.804	1.071
butyl hexanoate	ester	13:05.2	00:01.7	**1422**	1428	0.023	0.042	0.049	0.096	0.039	0.063	0.092	0.068	0.014	0.026	0.010	0.039	0.000	0.000	0.016	0.021	0.049	0.000	0.012	0.007	0.015	0.014	0.009	0.010	0.031	0.013	0.019	0.011	0.012	0.010	0.014	0.000
ethyl hydroxyacetate	ester	13:09.0	00:00.8	**1424**	1436	0.000	0.000	0.000	0.077	0.000	0.000	0.071	0.105	0.094	0.187	0.000	0.069	0.173	0.000	0.157	0.151	0.166	0.092	0.000	0.000	0.000	0.000	0.000	0.094	0.000	0.107	0.000	0.000	0.392	0.000	0.082	0.407
isoamyl hexanoate	ester	14:05.0	00:01.8	**1462**	1458	8.146	5.514	8.100	8.199	7.569	8.501	6.960	4.048	1.478	2.912	1.304	3.704	0.511	1.370	1.351	3.910	4.256	1.280	1.077	5.794	9.717	1.557	3.614	2.137	3.714	3.365	4.310	2.480	3.644	3.274	1.204	0.132
octyl acetate	ester	14:24.4	00:01.5	**1475**	1475	0.875	0.000	0.000	0.000	0.000	0.000	2.389	0.000	2.235	2.970	2.244	0.000	0.197	1.267	2.293	3.471	0.000	0.000	1.914	2.412	0.000	2.163	2.714	3.251	2.191	2.997	5.815	4.441	0.660	0.760	18.752	0.227
ethyl 4-pyrazolecarboxylate	ester	14:33.0	00:01.1	**1481**		0.017	0.015	0.017	0.013	0.019	0.015	0.015	0.015	0.019	0.020	0.015	0.023	0.024	0.036	0.045	0.022	0.013	0.023	0.021	0.000	0.000	0.021	0.010	0.014	0.009	0.012	0.000	0.012	0.027	0.014	0.016	0.000
ethyl 2,4-hexadienoate I	ester	14:35.0	00:01.3	**1483**	1501	0.008	0.025	0.086	0.085	0.037	0.011	0.007	0.006	0.012	0.036	0.008	0.033	0.011	0.009	0.020	0.007	0.011	0.027	0.016	6.015	0.000	0.009	0.044	0.009	0.033	0.004	2.282	0.023	0.007	0.009	0.007	0.273
ethyl *cis*-4-octenoate	ester	14:39.6	00:01.5	**1486**		0.276	0.137	0.049	0.128	0.039	0.126	0.105	0.159	0.104	0.118	0.068	0.200	0.061	0.069	0.036	0.136	0.135	0.054	0.075	0.146	0.214	0.101	0.116	0.085	0.087	0.100	0.122	0.090	0.085	0.117	0.186	0.000
ethyl 2,4-hexadienoate II	ester	15:08.0	00:01.3	**1506**	1501	0.033	0.063	0.748	0.778	0.490	0.052	0.068	0.091	0.029	0.036	0.024	0.073	0.040	0.032	0.030	0.023	0.071	0.017	0.026	43.141	0.097	0.028	0.564	0.034	0.104	0.032	23.164	0.386	0.029	0.080	0.033	0.947
ethyl *trans*-4-octenoate	ester	15:15.0	00:01.5	**1512**		0.034	0.029	0.026	0.031	0.034	0.027	0.033	0.034	0.018	0.023	0.015	0.030	0.017	0.000	0.018	0.030	0.031	0.014	0.016	0.026	0.026	0.022	0.019	0.017	0.000	0.018	0.020	0.019	0.022	0.021	0.021	0.011
ethyl 3-hydroxybutyrate	ester	15:15.5	00:00.9	**1512**	1512	0.509	0.397	0.305	0.565	0.000	0.009	0.725	0.674	0.362	0.416	0.510	0.392	0.627	0.000	0.540	0.543	0.671	0.462	0.061	0.368	0.370	0.535	0.196	0.373	0.330	0.397	0.288	0.000	0.048	0.323	0.479	0.489
propyl octanoate	ester	15:22.0	00:01.8	**1518**	1504	0.807	0.874	0.548	1.280	0.498	0.920	1.177	1.045	0.189	0.536	0.393	1.350	0.162	0.158	0.205	0.448	0.896	0.111	0.078	0.394	0.395	0.364	0.566	0.454	1.118	0.424	0.819	0.410	0.246	0.566	0.170	0.073
isopropyl lactate	ester	15:36.0	00:01.6	**1530**		0.024	0.022	0.041	0.028	0.036	0.035	0.042	0.030	0.028	0.031	0.023	0.027	0.024	0.024	0.033	0.047	0.000	0.034	0.036	0.029	0.019	0.041	0.020	0.029	0.023	0.034	0.017	0.024	0.038	0.000	0.047	0.022
ethyl 3-ethoxy-*trans*-2-proipenoate	ester	15:43.0	00:01.2	**1536**		1.306	1.398	1.533	1.294	1.460	1.449	1.548	1.356	1.485	1.446	1.448	1.110	1.051	1.391	1.608	1.624	1.334	1.549	1.478	1.412	1.310	1.655	1.478	1.719	1.205	1.604	1.194	1.480	2.112	1.415	1.682	0.983
ethyl nonanoate	ester	15:43.0	00:01.7	**1536**	1535	1.202	2.533	2.305	2.327	1.420	1.651	1.929	1.381	1.084	1.651	0.888	0.767	0.452	0.761	1.282	1.317	1.530	0.617	0.808	0.812	1.056	0.659	0.994	0.664	9.904	1.422	0.927	0.941	1.398	1.438	1.712	0.198
ethyl 2-hydroxy-4-methylpentanoat	ester	15:49.3	00:01.0	**1541**		0.369	0.000	0.483	0.498	0.425	0.388	0.408	0.472	0.550	0.833	0.997	0.450	1.214	1.762	0.000	1.788	2.643	3.591	1.665	1.253	0.466	1.723	0.775	1.319	0.837	2.069	0.971	1.472	3.935	0.399	0.400	0.000
isobutyl octanoate	ester	15:57.7	00:01.9	**1548**	1551	0.270	0.169	0.303	0.146	0.191	0.305	0.227	0.122	0.093	0.164	0.094	0.286	0.032	0.081	0.082	0.223	0.088	0.153	0.047	0.349	0.312	0.123	0.143	0.173	0.166	0.169	0.185	0.198	0.457	0.147	0.064	0.010
acetic formic anhydride	other	15:59.6	00:00.7	**1549**		0.117	0.138	0.000	0.023	0.000	0.000	0.240	0.000	0.269	0.165	0.073	0.095	0.000	0.120	0.393	0.260	0.052	0.193	0.245	0.000	0.094	0.214	0.017	0.023	0.015	0.000	0.021	0.106	0.233	0.020	0.100	0.000
ethyl 2-octenoate	ester	16:04.0	00:01.5	**1553**	1557	0.021	0.033	0.031	0.037	0.029	0.033	0.029	0.034	0.020	0.028	0.020	0.037	0.025	0.019	0.041	0.025	0.021	0.014	0.016	0.035	0.034	0.024	0.017	0.023	0.024	0.017	0.016	0.017	0.020	0.044	0.021	0.016
octyl formate	ester	16:04.2	00:01.0	**1553**	1560	5.761	5.957	6.116	6.742	7.156	5.494	7.310	8.294	7.063	6.258	6.722	4.640	7.821	4.820	6.542	8.414	4.739	4.799	8.269	6.404	6.349	6.746	8.244	9.942	10.229	9.213	7.538	11.170	7.903	7.732	3.455	6.963
2-(1,1-dimethylethyl)-cyclohexen-1-	ester	16:11.0	00:01.8	**1559**		0.076	0.081	0.000	0.047	0.048	0.045	0.049	0.023	0.035	0.000	0.019	0.034	0.016	0.022	0.046	0.051	0.060	0.040	0.040	0.027	0.000	0.100	0.034	0.040	0.038	0.018	0.028	0.013	0.110	0.037	0.035	0.030
trans, *trans*-2,4-octadien-1-yl acetate	ester	16:18.0	00:01.4	**1565**		0.023	0.019	0.030	0.000	0.018	0.000	0.000	0.050	0.037	0.037	0.022	0.024	0.000	0.000	0.045	0.000	0.018	0.000	0.000	0.000	0.000	0.021	0.000	0.000	0.018	0.000	0.012	0.018	0.000	0.034	0.017	0.000
isoamyl lactate	ester	16:18.0	00:01.0	**1565**	1572	0.378	0.192	0.402	0.303	1.146	0.232	0.374	0.565	0.418	3.224	0.386	0.285	0.643	0.356	0.489	0.000	0.998	0.671	0.463	0.927	0.232	0.887	0.281	0.535	0.428	0.584	0.513	0.580	1.998	0.435	0.365	0.988
diethyl malonate	ester	16:32.0	00:01.1	**1577**	1574	0.107	0.113	0.270	0.163	0.178	0.176	0.214	0.169	0.139	0.230	0.119	0.156	0.347	0.141	0.181	0.245	0.138	0.161	0.111	0.113	0.099	0.169	0.083	0.148	0.108	0.162	0.094	0.123	0.165	0.183	0.171	0.236
methyl 2-methyllactate	ester	16:39.0	00:01.2	**1582**		0.107	0.093	0.128	0.115	0.116	0.106	0.141	0.119	0.092	0.098	0.083	0.102	0.082	0.064	0.095	0.100	0.080	0.124	0.114	0.101	0.080	0.116	0.100	0.116	0.095	0.110	0.087	0.094	0.159	0.103	0.100	0.000
ethyl 3-nonenoate	ester	16:44.2	00:01.6	**1587**		0.112	0.019	0.064	0.071	0.016	0.122	0.089	0.044	0.010	0.059	0.010	0.041	0.007	0.007	0.014	0.017	0.016	0.010	0.013	0.010	0.001	0.008	0.014	0.005	0.047	0.010	0.011	0.028	0.008	0.014	0.005	0.000
methyl decanoate	ester	16:53.0	00:01.6	**1594**	1593	1.247	1.814	1.336	1.314	0.753	0.997	0.893	2.643	0.496	0.924	0.623	0.468	0.166	0.595	0.590	0.527	0.900	0.633	0.390	1.044	1.000	0.691	0.657	0.654	0.849	0.479	0.543	0.502	0.509	0.771	0.425	0.082
ethyl methyl succinate	ester	17:36.4	00:01.1	**1638**	1631	0.237	0.253	0.201	0.461	0.249	0.285	0.437	0.654	0.198	0.547	0.172	0.332	0.339	0.230	0.326	0.335	0.277	0.221	0.131	0.212	0.179	0.336	0.000	0.000	0.073	0.196	0.106	0.076	0.099	0.179	0.318	0.266
hydroxyl acid ester (n.i.)	ester	17:49.0	00:01.3	**1651**		0.000	0.000	0.013	0.006	0.000	0.006	0.008	0.008	0.005	0.012	0.006	0.016	0.009	0.014	0.018	0.017	0.008	0.008	0.005	0.007	0.000	0.016	0.000	0.007	0.005	0.011	0.004	0.007	0.012	0.010	0.019	0.011
diethyl fumarate	ester	17:56.0	00:01.2	**1659**	1660	0.024	0.033	0.073	0.100	0.041	0.032	0.082	0.053	0.029	0.024	0.035	0.060	0.066	0.048	0.069	0.113	0.023	0.058	0.041	0.033	0.025	0.044	0.034	0.050	0.043	0.058	0.041	0.046	0.077	0.061	0.033	0.040
isoamyl octanoate	ester	18:06.1	00:01.9	**1669**	1657	4.324	4.941	3.822	3.707	3.198	5.079	4.746	3.244	2.068	3.156	2.301	3.584	1.094	1.884	2.296	3.024	3.459	1.940	1.598	4.359	5.384	2.322	3.451	3.049	4.385	3.484	3.809	2.756	5.589	3.867	1.937	0.572
ethyl *cis*-4-decenoate	ester	18:17.4	00:01.6	**1681**	1680	0.035	0.035	0.040	0.000	0.000	0.000	0.000	0.027	0.010	0.019	0.019	0.025	0.000	0.012	0.000	0.000	0.000	0.000	0.000	0.021	0.034	0.000	0.037	0.013	0.016	0.000	0.009	0.016	0.016	0.018	0.014	0.000
diethyl succinate	ester	18:24.2	00:01.2	**1688**	1688	42.175	43.378	27.699	50.169	52.178	41.882	52.793	26.960	22.286	185.280	25.079	54.701	50.340	44.972	27.374	41.690	45.422	27.567	24.495	32.968	42.875	32.878	17.825	22.872	21.986	22.900	22.032	22.205	24.258	26.223	22.997	47.368
ethyl 9-decenoate	ester	18:45.0	00:01.6	**1708**	1708	0.139	0.036	0.592	0.053	0.042	0.114	0.031	0.060	0.079	0.674	0.097	0.198	0.011	0.131	0.050	0.042	0.406	0.161	0.034	0.074	1.010	0.232	0.139	0.046	0.041	0.055	0.061	0.086	0.280	0.060	0.129	0.013
ethyl undecanoate	ester	18:45.2	00:01.0	**1709**	1725	2.290	0.204	0.000	0.000	0.266	0.325	0.472	0.755	0.174	0.310	0.127	0.001	0.322	0.233	0.216	0.002	0.816	0.000	0.243	0.918	0.002	0.251	0.220	2.210	0.705	1.265	1.011	1.629	0.000	0.357	0.105	0.037
phenylmethyl acetate	ester	19:27.0	00:01.2	**1745**	1747	0.049	0.017	0.041	0.049	0.016	0.039	0.025	0.097	1.102	0.109	0.082	0.024	0.051	0.021	0.089	0.011	0.050	0.053	0.034	0.029	0.013	0.055	0.067	0.051	0.062	0.024	0.035	0.059	0.026	0.062	0.031	0.070
vinyl decanoate	ester	19:27.2	00:01.4	**1745**		0.000	0.076	1.067	0.150	0.018	0.096	0.045	0.338	0.082	0.033	0.040	0.123	0.216	0.042	0.063	0.054	0.125	0.026	0.025	0.041	0.016	0.022	0.022	0.031	0.111	0.023	0.044	0.020	0.013	0.153	0.178	0.060
di-isobutyl acetate	ester	20:02.0	00:01.2	**1775**		0.154	0.145	0.223	0.329	0.189	0.200	0.266	0.145	0.089	0.651	0.138	0.342	0.314	0.087	0.080	0.340	0.215	0.072	0.050	0.066	0.078	0.150	0.067	0.120	0.126	0.182	0.100	0.098	0.088	0.128	0.064	0.091
diethyl glutarate	ester	20:23.0	00:01.2	**1793**	1780	0.020	0.017	0.040	0.041	0.011	0.024	0.044	0.024	0.063	0.085	0.061	0.094	0.101	0.095	0.239	0.067	0.048	0.081	0.065	0.032	0.011	0.144	0.044	0.056	0.061	0.077	0.043	0.064	0.068	0.077	0.084	0.115
phenethyl formate	ester	20:29.8	00:01.1	**1799**	1806	0.110	0.099	0.082	0.071	0.018	0.123	0.123	0.082	0.316	0.156	0.212	0.141	0.135	0.272	0.244	0.136	0.084	0.208	0.193	0.143	0.199	0.129	0.108	0.113	0.090	0.116	0.085	0.111	0.213	0.097	0.090	0.150
methyl 2-isopropoxypropanoate	ester	20:30.0	00:01.3	**1799**		0.000	0.089	0.000	0.000	0.000	0.000	0.061	0.090	0.090	0.092	0.104	0.000	0.081	0.098	0.070	0.075	0.000	0.083	0.075	0.100	0.000	0.096	0.103	0.092	0.000	0.000	0.092	0.071	0.095	0.091	0.067	0.000
butyl ethyl succinate	ester	20:37.0	00:01.3	**1806**	1820	0.317	0.114	0.399	0.197	0.341	0.191	0.307	0.091	0.281	0.703	0.271	0.304	0.251	0.173	0.084	0.561	0.160	0.363	0.066	0.281	0.473	0.154	0.106	0.171	0.111	0.297	0.157	0.196	0.499	0.121	0.083	0.205
ethyl 4-hydroxybutyrate	ester	20:47.7	00:00.9	**1815**	1819	0.622	0.982	0.357	0.482	0.932	2.587	1.163	0.513	1.804	0.682	1.207	0.292	1.604	0.412	1.935	0.256	0.762	0.901	1.386	1.382	2.450	0.852	0.267	0.355	0.278	0.305	0.142	0.192	0.667	0.525	0.345	0.739
2-phenethyl acetate	ester	20:58.0	00:01.2	**1825**	1828	19.917	16.361	13.892	16.605	47.814	21.399	43.114	20.548	27.738	19.390	21.693	22.817	21.487	17.016	15.769	47.572	24.698	15.190	17.744	14.387	9.539	14.722	16.754	40.883	15.620	15.791	14.163	14.690	15.512	15.350	11.980	14.663
3-hydroxy-2,4,4-trimethylpentyl isol	ester	21:54.7	00:01.2	**1876**		0.121	0.072	0.080	0.062	0.109	0.094	0.089	0.082	0.248	0.221	0.119	0.148	0.254	0.167	0.183	0.070	0.038	0.332	0.122	0.000	0.153	0.150	0.126	0.151	0.112	0.056	0.097	0.063	0.127	0.146	0.063	0.126
isoamyl decanoate	ester	21:58.0	00:01.9	**1879**	1871	0.000	0.265	0.000	0.000	0.000	0.000	0.000	0.045	0.332	0.188	0.269	0.000	0.091	0.268	0.192	0.000	0.000	0.338	0.186	0.382	0.107	0.247	0.266	0.243	0.205	0.209	0.219	0.213	0.321	0.253	0.237	0.046
ethyl 3-methylbutylbutanedioate	ester	22:29.7	00:01.3	**1907**	1907	2.585	2.051	2.178	2.312	4.111	1.669	2.147	1.467	3.040	8.172	3.086	3.143	3.419	1.852	0.761	2.847	1.536	2.366	0.981	3.202	6.473	1.799	1.972	2.476	2.141	3.081	2.716	2.802	4.711	2.135	1.055	1.726
2-phenylethyl isobutyrate	ester	22:45.7	00:01.1	**1921**	1916	8.227	2.286	3.233	3.189	2.895	3.550	4.187	2.735	0.749	0.000	1.044	4.092	4.378	1.034	1.474	0.580	3.950	1.528	1.444	0.057	1.501	0.890	0.398	1.029	0.489	1.575	0.727	0.857	0.640	0.959	0.855	0.492
2-phenylethyl isovalerate	ester	24:00.0	00:01.4	**1985**	1988	0.012	0.034	0.013	0.000	0.000	0.015	0.027	0.017	0.033	0.026	0.015	0.000	0.000	0.000	0.022	0.000	0.008	0.021	0.026	0.016	0.000	0.019	0.012	0.020	0.018	0.017	0.020	0.020	0.031	0.018	0.014	0.000
diethyl malate	ester	25:29.4	00:00.9	**2063**	2065	0.028	0.268	0.342	0.372	0.139	0.019	0.320	0.328	0.194	0.133	0.299	0.478	0.640	0.000	1.491	0.570	0.441	0.681	0.334	0.147	0.091	0.527	0.170	0.517	0.249	0.633	0.123	0.181	0.650	0.822	0.698	0.367
trans,*trans*-2,4-hexadienoic acid	ester	26:51.6	00:00.8	**>2100**	2150	0.209	0.408	0.000	0.305	0.061	0.236	0.258	0.029	0.000	0.021	0.008	0.162	0.129	0.050	0.000	0.026	0.343	0.000	0.000	127.603	0.461	0.091	0.972	0.008	0.705	0.008	28.288	0.465	0.000	0.150	0.000	6.558
ethyl 2-hydroxy-3-phenylpropanoat	ester	29:11.1	00:01.0	**>2100**	2273	0.000	0.002	0.002	0.001	0.001	0.001	0.000	0.001	0.000	0.000	0.001	0.002	0.002	0.002	0.002	0.001	0.001	0.051	0.002	0.001	0.002	0.007	0.001	0.001	0.001	0.004	0.001	0.001	0.039	0.001	0.001	0.001
ethyl hexadecanoate	ester	30:13.7	00:01.5	**>2100**	2261	0.506	0.597	0.905	0.499	0.000	1.187	0.000	0.789	0.015	0.184	0.000	0.000	0.155	0.000	0.000	0.000	0.222	0.008	0.000	0.017	0.589	0.000	0.014	0.204	0.009	0.000	0.000	0.386	0.023	0.020	0.125	0.010
ethyl hydrogen succinate	ester	30:16.2	00:00.8	**>2100**	2350	3.231	3.791	2.128	5.036	3.010	3.033	3.697	2.308	6.138	21.831	4.694	3.104	4.505	3.529	7.903	4.911	8.030	8.050	5.735	3.973	3.748	6.679	2.466	4.107	1.952	4.163	7.120	8.533	6.557	5.515	4.143	3.069
2-phenylethyl octanoate	ester	32:17.5	00:01.3	**>2100**	2373	0.011	0.000	0.048	0.068	0.013	0.100	0.014	0.000	0.012	0.011	0.008	0.008	0.000	0.004	0.000	0.011	0.000	0.010	0.011	0.008	0.009	0.011	0.006	0.008	0.008	0.011	0.084	0.007	0.043	0.000	0.009	0.005
2-methoxyphenol	phenol	21:47.0	00:00.9	**1869**	1869	0.013	0.009	0.011	0.019	0.058	0.017	0.020	0.035	0.024	0.024	0.054	0.123	0.030	0.057	0.050	0.012	0.014	0.033	0.036	0.014	0.012	0.015	0.009	0.010	0.010	0.011	0.009	0.011	0.012	0.012	0.008	0.014
phenol	phenol	24:14.0	00:00.8	**1998**	1995	0.673	0.733	0.675	0.632	0.679	0.731	0.790	0.694	0.897	0.700	0.769	1.011	1.035	0.739	0.843	0.720	0.689	0.647	0.779	1.496	0.648	0.647	0.485	0.543	0.550	0.550	0.816	0.506	0.758	0.618	0.532	1.509
4-ethylguaiacol	phenol	24:44.7	00:01.0	**2024**	2024	0.000	0.000	0.058	0.020	0.010	0.004	0.000	0.000	0.099	0.030	0.004	0.009	0.018	0.011	0.000	0.009	0.002	0.000	0.000	0.006	0.000	0.009	0.013	0.000	0.002	0.000	0.000	0.000	0.000	0.000	0.006	0.004
4-vinylguaiacol	phenol	27:33.5	00:00.9	**>2100**	2168	0.728	0.456	0.502	0.080	0.716	0.975	0.627	0.418	0.948	1.021	0.576	0.174	1.421	0.355	0.838	0.329	0.290	0.571	0.232	0.531	0.133	0.525	0.137	0.328	0.158	0.127	0.311	0.522	1.055	0.227	0.090	0.173
2,4-bis(1,1-dimethylethyl)phenol	phenol	29:01.0	00:01.0	**>2100**	2270	0.676	0.601	0.729	1.591	0.733	0.623	0.845	1.584	1.181	0.812	0.990	0.494	0.904	0.516	0.851	0.782	0.695	2.198	1.125	1.328	0.431	1.376	0.529	0.847	0.121	0.699	0.732	0.745	0.999	0.924	0.913	0.592
furfuryl ether	furanoids an	10:14.0	00:01.1	**1292**		0.073	0.066	0.147	0.092	0.613	0.053	0.078	0.154	0.123	0.382	0.227	0.137	0.158	0.253	0.415	0.266	0.138	0.360	0.293	0.201	0.108	0.353	0.057	0.101	0.054	0.137	0.066	0.095	0.127	0.089	0.197	0.063
furfural	furanoids an	14:05.2	00:00.9	**1462**	1460	0.759	1.047	0.852	1.859	0.714	1.261	1.084	1.397	2.300	2.615	3.219	3.996	4.051	73.911	28.567	2.904	0.963	3.944	2.454	1.752	1.171	2.618	0.816	0.920	0.635	0.997	0.746	0.758	1.125	0.822	0.843	1.363
2-(5-methyl-5-vinyltetrahydro-2-fura	furanoids an	14:16.1	00:01.2	**1470**		0.123	0.000	0.244	0.000	0.000	0.000	0.000	0.031	0.104	0.141	0.078	0.072	0.166	0.000	0.184	0.073	0.013	0.140	0.000	0.011	0.000	0.136	0.063	0.088	0.185	0.000	0.134	0.010	0.218	0.172	0.573	0.169
acetylfuran	furanoids an	15:01.2	00:01.0	**1501**	1501	0.054	0.068	0.106	0.095	0.200	0.016	0.112	0.082	0.197	0.241	0.169	0.244	0.430	1.089	0.541	0.319	0.149	0.184	0.210	0.113	0.075	0.358	0.047	0.104	0.051	0.114	0.050	0.082	0.153	0.065	0.086	0.126
5-methyl-2-furfural	furanoids an	16:25.8	00:01.0	**1571**	1570	0.000	0.015	0.013	0.000	0.021	0.041	0.000	0.023	0.056	0.054	0.085	0.154	0.116	11.570	1.762	0.137	0.039	0.000	0.000	0.042	0.054	0.115	0.023	0.029	0.025	0.048	0.024	0.029	0.055	0.017	0.064	0.000
ethyl 2-furoate	furanoids an	17:21.0	00:01.1	**1622**	1624	2.418	3.638	4.907	5.580	4.614	5.643	6.577	5.061	7.388	7.822	3.442	4.381	10.534	6.804	9.250	6.346	5.176	5.289	7.032	2.370	1.640	6.646	4.452	7.519	5.630	6.713	4.119	6.031	3.924	3.188	4.527	6.643
γ-butyrolactone	furanoids an	17:28.0	00:01.0	**1629**	1626	3.166	4.102	2.422	3.309	3.838	6.581	4.216	3.081	5.134	3.054	3.797	2.718	5.128	3.195	6.961	3.223	3.819	3.454	4.059	3.812	5.835	4.187	1.408	1.733	1.463	1.693	1.120	1.250	4.756	2.246	2.105	3.633
3-methyl-2(5H)-furanone	furanoids an	19:13.3	00:01.0	**1733**	1726	0.019	0.020	0.015	0.032	0.049	0.036	0.041	0.021	0.023	0.025	0.005	0.038	0.028	0.017	0.030	0.021	0.024	0.015	0.026	0.015	0.015	0.000	0.000	0.009	0.000	0.009	0.007	0.000	0.000	0.014	0.014	0.024
5-ethoxydihydro-2(3H)-furanone	furanoids an	19:20.0	00:01.0	**1739**	1728	0.038	0.034	0.039	0.046	0.100	0.048	0.081	0.043	0.051	0.077	0.081	0.000	0.094	0.045	0.066	0.119	0.049	0.105	0.071	0.044	0.035	0.074	0.031	0.031	0.027	0.039	0.054	0.039	0.105	0.049	0.030	0.106
2(5H)-furanone	furanoids an	19:59.4	00:00.9	**1773**	1787	0.078	0.079	0.053	0.075	0.087	0.097	0.091	0.074	0.087	0.062	0.083	0.538	0.110	0.073	0.101	0.091	0.067	0.075	0.079	0.098	0.069	0.064	0.056	0.077	0.046	0.063	0.051	0.063	0.061	0.051	0.054	0.133
δ-caprolactone	furanoids an	20:37.4	00:01.1	**1806**	1818	0.125	0.621	0.160	0.290	0.165	0.440	0.718	0.232	0.223	0.320	0.198	0.688	0.333	0.147	0.412	0.488	0.223	0.521	0.388	0.206	0.154	0.520	0.155	0.314	0.305	0.300	0.209	0.183	0.671	0.186	0.401	0.278
γ-octalactone	furanoids an	22:49.8	00:01.1	**1924**	1923	0.255	0.689	0.444	0.651	0.362	0.360	0.864	0.642	0.872	0.860	0.746	1.536	0.755	0.569	1.009	0.458	0.307	1.025	1.161	0.594	0.493	0.919	0.451	0.872	0.687	0.750	0.393	0.623	1.287	0.677	0.768	0.746
lactone (n.i.)	furanoids an	23:39.0	00:01.1	**1967**		0.023	0.015	0.090	0.052	0.113	0.160	0.000	0.000	0.010	0.009	0.000	0.000	0.032	0.006	0.033	0.103	0.029	0.018	0.017	0.000	0.027	0.076	0.007	0.008	0.007	0.014	0.006	0.006	0.022	0.007	0.009	0.010
lactone (n.i.)	furanoids an	23:41.8	00:01.2	**1970**		0.003	0.011	0.000	0.000	0.020	0.000	0.000	0.063	0.070	0.056	0.035	0.000	0.000	0.621	0.066	0.000	0.006	0.042	0.096	0.000	0.009	0.000	0.000	0.005	0.000	0.052	0.040	0.038	0.037	0.061	0.000	0.058
pantolactone	furanoids an	24:42.0	00:00.8	**2022**	2029	0.083	0.110	0.068	0.113	0.072	0.084	0.090	0.110	0.156	0.162	0.152	0.146	0.259	0.099	0.314	0.145	0.164	0.395	0.121	0.161	0.090	0.144	0.092	0.229	0.112	0.136	0.140	0.115	0.271	0.168	0.075	0.127
γ-nonalactone	furanoids an	24:42.6	00:01.2	**2022**	2018	0.021	0.075	0.000	0.000	0.000	0.000	0.000	0.202	0.421	0.449	0.208	0.000	0.665	0.133	0.299	0.000	0.072	0.171	0.313	0.228	0.018	0.260	0.126	0.079	0.323	0.102	0.268	0.194	0.305	0.294	0.136	0.144
γ-hydroxymethyl-γ-butyrolactone	furanoids an	28:33.0	00:00.9	**>2100**		0.827	0.591	1.083	3.796	1.030	1.257	1.949	2.217	1.234	0.000	1.756	1.598	2.919	1.856	3.637	3.802	3.561	3.737	2.598	0.000	0.620	4.956	2.007	3.012	2.949	4.903	0.000	0.000	4.225	0.000	2.832	2.372
methional	sulfur contai	14:02.7	00:01.0	**1461**	1461	0.015	0.049	0.010	0.009	0.016	0.012	0.007	0.023	0.097	0.078	0.134	0.063	0.088	0.189	0.161	0.021	0.057	0.095	0.068	0.024	0.134	0.021	0.016	0.011	0.026	0.015	0.016	0.016	0.022	0.012	0.007	0.062
3-ethoxythiophene	sulfur contai	14:19.0	00:01.2	**1472**		0.016	0.031	0.019	0.057	0.019	0.024	0.028	0.050	0.022	0.028	0.021	0.045	0.013	0.116	0.052	0.073	0.047	0.089	0.037	0.021	0.000	0.111	0.018	0.026	0.018	0.029	0.017	0.029	0.027	0.030	0.030	0.000
S-(3-hydroxypropyl) thioacetate	sulfur contai	14:47.0	00:01.1	**1491**		0.053	0.077	0.032	0.063	0.039	0.061	0.049	0.041	0.076	0.141	0.101	0.075	0.080	0.038	0.072	0.049	0.094	0.052	0.056	0.048	0.067	0.066	0.048	0.055	0.049	0.056	0.042	0.042	0.043	0.045	0.020	0.059
2-methyldihydro-3(2H)-thiophenon	sulfur contai	15:28.6	00:01.1	**1523**	1538	0.930	1.147	1.573	1.615	2.477	1.121	2.227	1.951	1.583	2.365	0.505	1.305	2.087	0.711	1.370	1.843	1.389	2.749	0.681	0.356	0.285	0.608	0.853	1.576	0.893	1.371	0.797	1.718	2.936	0.880	1.068	1.757
2-(methylthio)ethanol	sulfur contai	15:29.0	00:00.8	**1524**	1531	0.206	0.263	0.235	0.191	0.260	0.277	0.256	0.398	0.356	0.423	0.450	0.346	0.378	0.280	0.262	0.444	0.371	0.190	0.191	0.247	0.278	0.306	0.143	0.123	0.125	0.172	0.123	0.110	0.132	0.169	0.188	0.355
diethyl sulfate	sulfur contai	17:21.0	00:01.0	**1622**		0.012	0.015	0.007	0.014	0.014	0.016	0.011	0.014	0.010	0.011	0.006	0.006	0.010	0.010	0.016	0.015	0.014	0.009	0.011	0.007	0.006	0.011	0.007	0.009	0.008	0.008	0.007	0.008	0.012	0.013	0.012	0.009
3-(methylthio)propyl acetate	sulfur contai	17:28.7	00:01.2	**1630**	1627	0.430	0.529	0.190	0.603	0.524	0.368	0.836	1.229	0.717	1.394	0.737	0.374	0.698	0.404	0.796	0.045	0.624	0.916	0.465	0.196	0.160	0.289	0.422	0.427	0.524	0.444	0.317	0.417	0.436	0.262	0.103	0.243
cyclohexyl isothiocyanate	sulfur contai	18:17.0	00:01.6	**1681**	1667	0.016	0.009	0.013	0.013	0.039	0.010	0.000	0.013	0.018	0.022	0.036	0.012	0.000	0.011	0.019	0.012	0.007	0.029	0.024	0.014	0.009	0.026	0.007	0.015	0.006	0.031	0.009	0.013	0.017	0.009	0.009	0.000
ethyl methanesulfonate	sulfur contai	18:31.0	00:00.9	**1695**		0.178	0.198	0.112	0.186	0.190	0.202	0.175	0.177	0.142	0.158	0.150	0.089	0.164	0.204	0.189	0.209	0.182	0.131	0.144	0.139	0.174	0.134	0.130	0.120	0.117	0.106	0.102	0.101	0.166	0.158	0.138	0.194
2-thiophenecarboxaldehyde	sulfur contai	18:45.0	00:01.0	**1708**	1701	0.016	0.042	0.020	0.061	0.026	0.045	0.050	0.054	0.054	0.037	0.033	0.133	0.084	0.125	0.139	0.121	0.059	0.081	0.069	0.025	0.013	0.074	0.011	0.049	0.033	0.027	0.020	0.035	0.046	0.047	0.078	0.083
methionol	sulfur contai	19:08.8	00:00.9	**1729**	1733	1.905	2.759	1.307	1.651	2.549	2.544	2.755	3.280	6.055	3.990	4.062	1.442	3.912	3.044	5.650	3.577	1.259	4.069	3.753	1.903	3.834	2.993	1.150	1.591	1.465	2.368	0.801	1.396	3.418	1.444	0.800	2.152
1-(*tert*-butylsulfonyl)-2-octanol	sulfur contai	19:14.9	00:02.2	**1734**		0.000	0.127	0.307	0.147	0.297	0.085	0.360	0.138	0.257	0.319	0.264	0.123	0.000	0.113	0.276	0.158	0.135	0.348	0.161	0.285	0.255	0.201	0.113	0.269	0.062	0.268	0.208	0.101	0.099	0.106	0.152	0.000
ethyl 3-(methylthio)-*trans*-2-propen	sulfur contai	19:41.0	00:01.2	**1757**	1733	0.012	0.009	0.016	0.016	0.006	0.010	0.011	0.028	0.021	0.032	0.026	0.018	0.010	0.013	0.024	0.009	0.009	0.018	0.016	0.015	0.007	0.012	0.014	0.016	0.017	0.017	0.017	0.024	0.018	0.017	0.009	0.004
ethyl thiophene-2-carboxylate	sulfur contai	20:02.0	00:01.2	**1775**		0.028	0.023	0.018	0.024	0.032	0.024	0.029	0.013	0.019	0.026	0.020	0.021	0.020	0.017	0.017	0.021	0.011	0.017	0.023	0.012	0.021	0.018	0.014	0.022	0.016	0.021	0.012	0.017	0.019	0.014	0.011	0.010
3-[(2-hydroxyethyl)thio]-1-propanol	sulfur contai	20:58.0	00:00.9	**1825**		0.020	0.022	0.015	0.015	0.026	0.018	0.022	0.021	0.022	0.025	0.009	0.013	0.022	0.024	0.015	0.040	0.016	0.067	0.009	0.042	0.006	0.010	0.012	0.023	0.013	0.035	0.009	0.027	0.084	0.012	0.026	0.029
ethyl thiocyanate	sulfur contai	21:05.2	00:01.2	**1831**		0.054	0.350	0.215	0.271	0.300	0.287	0.298	0.225	0.294	0.463	0.433	0.241	0.147	0.184	0.329	0.397	0.336	0.218	0.324	0.132	0.281	0.270	0.217	0.249	0.216	0.300	0.177	0.208	0.393	0.158	0.108	0.069
4-(methylthio)-1-butanol	sulfur contai	21:26.0	00:00.9	**1850**		0.007	0.010	0.007	0.005	0.012	0.013	0.013	0.020	0.033	0.023	0.018	0.008	0.032	0.010	0.031	0.029	0.010	0.016	0.030	0.011	0.009	0.023	0.006	0.012	0.010	0.012	0.003	0.007	0.013	0.010	0.005	0.008
ethyl 3-(methylthio)-*trans*-2-propen	sulfur contai	21:33.0	00:01.2	**1856**	1837	0.000	0.000	0.006	0.003	0.000	0.000	0.000	0.011	0.015	0.016	0.012	0.006	0.005	0.003	0.016	0.000	0.000	0.010	0.013	0.007	0.000	0.010	0.007	0.009	0.013	0.000	0.011	0.020	0.008	0.012	0.004	0.000
S-[(2,5-dihydro-4-hydroxy-5-oxo-3-f	sulfur contai	23:09.7	00:00.8	**1942**		0.128	0.000	0.023	0.198	0.123	0.000	0.431	0.000	0.000	0.011	0.038	0.152	0.000	0.000	0.020	0.084	0.000	0.000	0.104	0.016	0.131	0.000	0.028	0.056	0.014	0.070	0.012	0.010	0.017	0.039	0.010	0.000
crotonic anhydride	other	21:25.8	00:01.5	**1850**		0.087	0.000	0.180	0.064	0.000	0.067	0.117	0.101	0.057	0.063	0.043	0.040	0.155	0.036	0.046	0.049	0.028	0.015	0.088	0.035	0.036	0.047	0.058	0.067	0.052	0.073	0.048	0.060	0.100	0.026	0.000	0.033
methylsuccinic anhydride	other	18:59.0	00:00.9	**1720**		0.013	0.045	0.000	0.000	0.000	0.000	0.048	0.000	0.000	0.020	0.018	0.305	0.000	0.012	0.000	0.056	0.075	0.000	0.024	0.068	0.016	0.029	0.010	0.046	0.000	0.047	0.000	0.066	0.032	0.048	0.063	0.047
ethylene diglycol monoethyl ether	other	17:19.0	00:00.9	**1620**	1622	0.054	0.074	0.070	0.062	0.078	0.052	0.127	0.130	0.359	0.357	0.259	0.036	0.279	0.157	0.222	0.062	0.103	0.178	0.266	0.324	0.103	0.188	0.200	0.246	0.167	0.144	0.254	0.250	0.327	0.250	0.251	0.280
1H-indole	other	31:00.2	00:00.9	**>2100**	2420	0.035	0.029	0.029	0.046	0.049	0.047	0.055	0.062	0.062	0.042	0.036	0.038	0.058	0.003	0.046	0.008	0.043	0.029	0.070	0.023	0.027	0.056	0.024	0.035	0.031	0.025	0.020	0.027	0.031	0.059	0.007	0.038
